# Well-defined nanomagnetic nitrilotriacetic acid complex of Cu(ii) supported on silica-coated nanosized magnetite: a new highly efficient and magnetically separable catalyst for C–N bond formation†

**DOI:** 10.1039/d4ra03675a

**Published:** 2024-07-11

**Authors:** Kimiya Rajabzadeh, Ali Reza Sardarian

**Affiliations:** a Department of Chemistry, Shiraz University Shiraz 71946-84795 Iran sardarian@shirazu.ac.ir

## Abstract

A nitrilotriacetic acid (NTA) complex of Cu(ii) supported on silica-coated nanosized magnetite Fe_3_O_4_@SiO_2_-Pr-DEA-[NTA-Cu(ii)]_2_ was prepared as a new well-defined magnetically separable nanomaterial and fully characterized *via* IR, XRD, FESEM, TEM, TGA, DLS, BET, VSM, solid-state UV-vis spectroscopy, EDX, ICP-OES, and FESEM-EDX map analyses. Thereafter, it was successfully applied as a new easily magnetically separable and reusable heterogeneous nanocatalyst for the Buchwald–Hartwig C–N bond formation reaction in DMF at 110 °C. Using this method, various kinds of nitrogen heterocycles, such as imidazoles, 2-methyl-1*H*-imidazole, benzimidazole, indole, and 10*H*-phenothiazine as well as aliphatic secondary amines such as piperidine, piperazine, morpholine, dimethylamine, and diethylamine, were reacted with aryl halide compounds, and the desired products were obtained with good to excellent yields. In all cases, the applied catalyst could be recovered easily and rapidly using an external magnet and reused 7 times without significant loss of catalytic activity.

## Abstract 2

Highly efficient catalyzed C–N bond formation by well-defined nitrilotriacetic acid Cu(ii) complex supported on silica coated nanosized magnetite as a novel magnetically separable catalyst.

## Graphical abstract

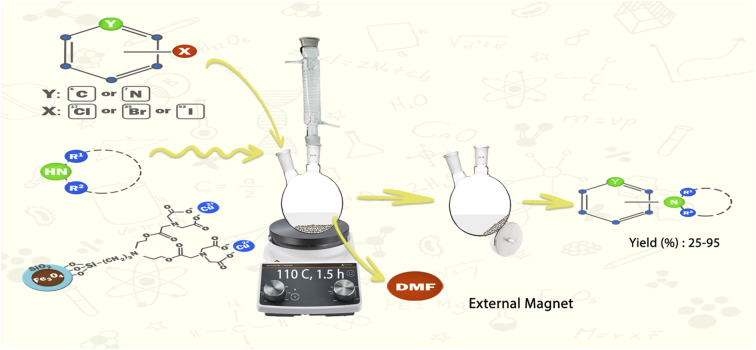

## Introduction

Considering the basic principles of green chemistry,^1^ a suitable catalyst exhibits the highest efficiency even at the lowest concentration and can be easily separated and reused.^2^ Because most of the chemical reactions take place on the surface of catalysts, catalysts with a higher surface-to-volume ratio will be more efficient at accelerating reactions. Accordingly, nanocatalysts that provide high surface-to-volume ratios are highly suitable as green catalysts because using them in very small quantities increases economic efficiency and prevents the release of hazardous substances into the environment. Moreover, there are other benefits, such as the manipulation of size, shape, composition, and morphology.^3^ All these advantages lead to the widespread application of nanocatalysts in several prominent industrial chemical processes, such as water purification,^4^ biodiesel production,^5^ photocatalysis,^6^ and drug delivery.^7^ Nevertheless, one of the most important problems in using nanocatalysts is the difficulty in separation due to the very small size of nanoparticles (NPs) and the need for special methods, such as ultrafiltration and high-speed centrifugation. Unfortunately, these methods are costly, involve time and energy, and are mostly inefficient, thus contradicting the green chemistry principles. One of the preferred strategies to overcome this problem is to magnetize nanocatalysts. Magnetic nanocatalysts can be easily and efficiently removed from reaction mixtures using an external magnetic field. Due to this special property, magnetic nanoparticles (MNPs), such as magnetite (δ-Fe_3_O_4_) and maghemite (ϒ-Fe_2_O_3_), have attracted much attention for use as substrates and catalysts among other iron oxide nanomaterials, leading to interesting findings.^8^ Magnetite (δ-Fe_3_O_4_) NPs with a wide range of advantages, such as tunable and very small particle sizes, high magnetic permeability, good stability, low cost, ease of preparation, and reasonably large specific surfaces, are the most important and popularly used MNPs in the design and preparation of novel magnetically separable nanocatalysts.^9^ Nevertheless, their high free surface energy and anisotropic dipolar attraction lead to the facile and rapid aggregation of δ-Fe_3_O_4_ NPs, resulting in the reduction of surface area and catalytic activity. A frequently used strategy to prevent the aggregation of δ-Fe_3_O_4_ NPs is coating them with SiO_2_, which provides a core–shell system.^10^ The Fe_3_O_4_@SiO_2_ core–shell system simultaneously provides the magnetic behaviour of δ-Fe_3_O_4_ and the tunable surface of SiO_2_ NPs, thus efficiently supporting the design and preparation of various types of magnetically separable heterogeneous nanocatalysts.

Arylamines and heteroarylamines are important precursors used for the synthesis of drugs, agrochemicals, and a wide range of natural products,^11^ complex molecules, such as dendrimers^12^ and polymers,^13^ dyes and pigments,^14^ and molecules with nonlinear optical features.^15^

Ever since Buchwald and Hartwig introduced palladium-mediated amination of aryl halides for the preparation of arylamine derivatives,^16^ many of the conditions have been improved, making the Buchwald–Hartwig method an extremely useful and synthetically vital technique. The lower reaction temperature, a wide range of available substrates, greater selectivity toward amines, better functional group compatibility, and the lack of formation of highly reactive species^17^ are the main advantages of the Buchwald–Hartwig method over other C–N bond formation strategies, such as nucleophilic aromatic substitution, Ullmann coupling, and nitration followed by reduction.^18^ However, the application of palladium, which is a very expensive and toxic catalyst, is an important drawback that overshadows the general use of the Buchwald–Hartwig method. Efforts to solve this problem have led to the use of copper as a more economical metal with vast abundance and consequently, low cost.^19^ Nevertheless, the currently reported methodologies suffer from numerous drawbacks, such as long reaction times, high reaction temperatures, the need for stoichiometric amounts of copper reagents, the use of toxic and air-sensitive ligands, and also very low yields; excessive amounts of aryl halides or amines are required to achieve reasonable product yields.^20^ Thus, the design and preparation of novel Cu-based catalytic systems to overcome these drawbacks are of great interest.

**Scheme 1:**
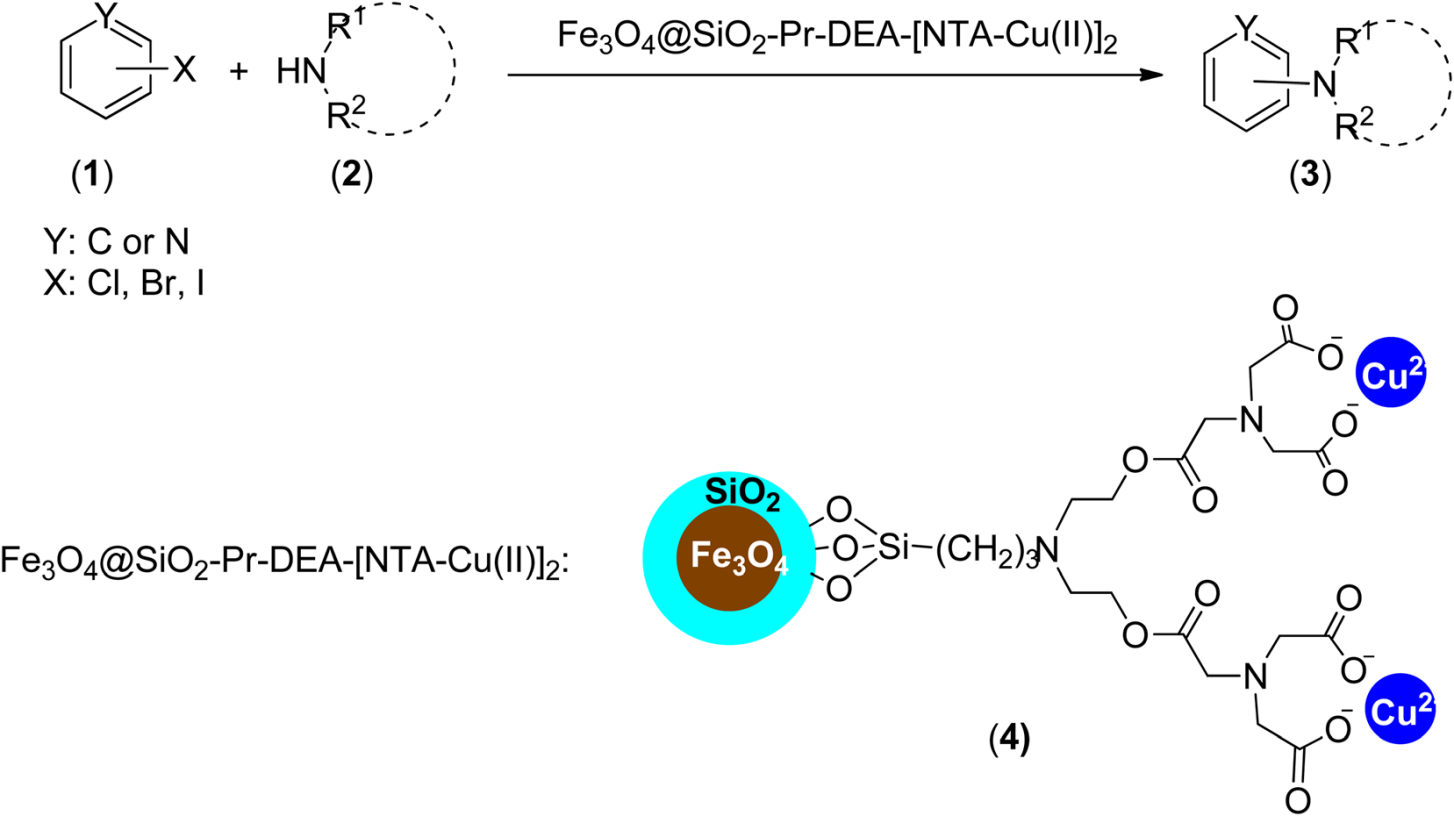
Fe_3_O_4_@SiO_2_-Pr-DEA-[NTA-Cu(ii)] as a new nanomagnetic catalyst for C–N bond formation.

Considering all the advantages of magnetically separable nanocatalysts and also the interesting features of single-atom catalysts, which provide unique opportunities for the design of effective, selective, and stable heterogeneous catalysts with well-defined active centres for a wide variety of chemical reactions,^21^ we introduce the NTA complex of Cu(ii) supported on silica-coated nanosized magnetite (Fe_3_O_4_@SiO_2_-Pr-DEA-[NTA-Cu(ii)]_2_) (4) as a novel well-defined nanomagnetic catalyst for the Buchwald–Hartwig C–N bond formation reaction (Scheme 1).

## Experimental methods

All chemicals were obtained from Sigma and Fluka and used as received without further purification. All the solvents were distilled and dried before use. The progress of the reactions was followed by TLC using silica gel polygrams SIL G/UV 254 plates. The eluent solvent used was petroleum ether, ethyl acetate, or a mixture of both. All yields refer to isolated products after indicated purification methods. The products were characterized by spectral data analysis. Their melting points were determined in open capillary tubes using a Büchi B-545 melting point apparatus. The inductively coupled plasma (ICP) analysis was carried out using an ICP analyzer (Varian, Vista-Pro). Elemental analyses were performed on a Thermo Finnigan CHNS analyzer (1112 series). The X-ray diffraction (XRD) patterns of the catalysts were recorded by a Bruker AXS D8-advance X-ray diffractometer using CuKα radiation (*λ* = 1.54178 Å) in 2θ scanning range of 20° to 90°. The samples were characterized by Field-Emission Scanning Electron Microscopy (FESEM-FEI, TESCAN, model MAIA3). Transmission electron microscopy (TEM) images were recorded using a Zeiss instrument operated at an accelerating voltage of 100 kV. The Brunauer–Emmett–Teller (BET) surface area analysis was adopted at 77 K to obtain the surface areas using a Belsorp mini II apparatus (Microtrac Bel Corp, Japan) after the samples were degassed using a flow of N_2_. To evaluate the thermal decomposition of the individual components and their mixture, Thermogravimetric Analysis (TGA) was performed on a PerkinElmer device manufactured by Thermal Sciences. UV-vis absorption spectra were measured on a UV-vis spectrophotometer (V_670-JASCO, Japan) with an integrating sphere attachment. Moreover, the synthesized samples were fully characterized by Dynamic Light Scattering (DLS) using a VASCO apparatus (CORDOUAN TECHNOLOGIES, France), and VSM curves were recorded on a Vibrating Sample Magnetometer Model VSM LBKFB Kashan Co. Shimadzu FT-IR 8300 and PerkinElmer FT-IR RX 1 spectrophotometers were applied for FT-IR measurements using KBr pellets in the range of 500 to 4000 wavenumbers (cm^−1^). NMR spectra were recorded on Bruker Avance DPX-250 (^1^H NMR 250 MHz), DPX-300 (^1^H NMR 300 MHz, ^13^C NMR 75 MHz), and DPX-400 (^1^H NMR 400 MHz, ^13^C NMR 101 MHz) spectrometers in pure deuterated chloroform (CDCl_3_) or deuterated dimethyl sulfoxide (DMSO-*d*_6_). The chemical shifts are given in parts per million (ppm) downfield from tetramethylsilane (TMS), which was used as an internal reference, and the coupling constants (J-values) are presented in hertz (Hz). Abbreviations of the ^1^H NMR assignment are as follows: singlet (s), doublet (d), triplet (t), quartet (q), doublet of doublets (dd), and multiplets (m).

### Preparation of Fe_3_O_4_ NPs

Magnetic Fe_3_O_4_ NPs were synthesized based on a modified co-precipitation method.^22^ Briefly, FeCl_3_·6H_2_O (1.3 g, 4.8 mmol) and FeCl_2_·4H_2_O (0.9 g, 4.5 mmol) were added to a surfactant solution of polyvinyl alcohol with a mean molecular weight of 15 000 Da (1 g) in water (30 mL), and the obtained mixture was stirred vigorously with a mechanical stirrer. The mixture was heated to 80 °C and then an appropriate amount (1.0 mole per liter of solution) of hexamethylenetetramine (HMTA) was added dropwise until the reaction media reached pH 10. The black magnetic particles of Fe_3_O_4_ formed were collected using an external magnet, washed with ethanol (50 mL, 3 times) and deionized water (50 mL, 3 times), and dried at 80 °C for 10 h.

### Preparation of Fe_3_O_4_@SiO_2_ NPs (5)

The Fe_3_O_4_@SiO_2_ NPs were prepared by a modified Stober method.^22^ In a typical procedure, the prepared Fe_3_O_4_ particles (0.5 g) were dispersed in a mixture of ethanol (50 mL), deionized water (5 mL), and tetraethoxysilane (TEOS) (0.188 g, 0.20 mL). Afterward, 5 mL of NaOH (10 wt%) was added dropwise. After stirring for 30 min at room temperature, the magnetic nanoparticles were collected using an external magnet and washed with ethanol (50 mL, 3 times) and deionized water (50 mL, 3 times) and dried at 80 °C for further uses.

### Preparation of *N*-propylchloride-functionalized silica-coated magnetite nanoparticles (Fe_3_O_4_@SiO_2_-Pr-Cl) (7)

To a 50 mL round-bottom flask containing 20 mL of ethanol, Fe_3_O_4_@SiO_2_ (0.6 g) was added and thoroughly dispersed under ultrasonic irradiation. After this, 3-chloromethoxypropylsilane (3 mmol, 0.4 g, 0.5 mL) was added, and the resulting mixture was refluxed for 12 h. After that, all the insoluble species were collected using an external magnet, washed with ethanol (50 mL, 3 times) and water (50 mL, 3 times), and dried at 80 °C for 12 h, and Fe_3_O_4_@SiO_2_-Pr-Cl nanoparticles were obtained as a brown powder.^23^

### Preparation of 2,2′-((3-propyl)azanediyl)bis(ethan-1-ol)-functionalized silica-coated MNPs (Fe_3_O_4_@SiO_2_-Pr-DEA) (9)

To a 50 mL round-bottom flask containing a solution of *N*,*N*-diisopropylethylamine (3 mmol, 0.38 g, 0.4 mL) in absolute ethanol (20 mL), diethanolamine (DEA) (3 mmol, 0.315 g, 0.3 mL) was added, and the solution was stirred at ambient temperature for 2 h. After this, Fe_3_O_4_@SiO_2_-Pr-Cl nanoparticles (1 g) were carefully added to the solution, and the obtained mixture was stirred vigorously for 12 h under reflux conditions. The Fe_3_O_4_@SiO_2_-Pr-DEA MNPs formed a brown powder, which was magnetically isolated from the crude using an external magnet. The dried magnetic nanoparticles were obtained after washing the obtained powder with ethanol (50 mL, 3 times) and water (50 mL, 3 times) and heating in an oven at 80 °C for 6 h.

### Synthesis of 2-(2,6-dioxomorpholino)acetic acid (10)

To a 10 mL round-bottom flask containing pyridine (1.5 mL), nitrilotriacetic acid (NTA) (1 g) was added, and the mixture was stirred for 30 minutes at ambient temperature to form a white precipitate. Afterward, acetic anhydride (1.5 mL) was added drop by drop (one drop every 20 seconds), and the flask was sealed and heated at 65 °C and stirred for 24 hours. After that, the mixture was concentrated under reduced pressure, and the obtained dark brown gummy solid was triturated with diethyl ether (3 times, 20 mL) until a chocolate-brown powder was formed. Pure 2-(2,6-dioxomorpholino)acetic acid (10) was obtained by drying the chocolate-brown powder at reduced pressure and used immediately after production.^24,25^

### Preparation of 3,15-bis(carboxymethyl)-5,13-dioxo-9-(3-propyl)-6,12-dioxa-3,9,15-triazaheptadecanedioic acid-functionalized silica-coated NMPs (Fe_3_O_4_@SiO_2_-Pr-DEA-[NTA]_2_) (11)

Fe_3_O_4_@SiO_2_-Pr-DEA MNPs (0.14 g) were added to a 10 mL round-bottom flask containing DMF (5 mL), and the mixture was sonicated for 20 min. After the mixture was completely dispersed, 2-(2,6-dioxomorpholino)acetic acid (10) (6 mmol, 0.57 g) was added, and the mixture was stirred at 75 °C for 20 h. After cooling down the reaction mixture to room temperature, the insoluble nanomagnetic particles (NMPs) (11) were collected and separated using an external magnet, washed with DMF (5 mL, 2 times) and deionized water (5 mL, 2 times), and dried under a vacuum at 65 °C for 8 h.

### Preparation of NTA complex of Cu(ii) supported on silica-coated nanosized magnetite (Fe_3_O_4_@SiO_2_-Pr-DEA-[NTA-Cu(ii)]_2_) (4)

Freshly synthesized Fe_3_O_4_@SiO_2_-Pr-DEA-[NTA]_2_ NMPs (0.13 g) were added to a 25 mL round-bottom flask containing 13 mL of ethanol, and the mixture was sonicated for 15 min. Afterward, copper(ii) acetate monohydrate (1 g) was added to the solution. The resulting mixture was refluxed for 6 h. Then, the reaction mixture was cooled to room temperature, and the insoluble materials were separated using an external magnet, washed with water (5 mL, 2 times) and ethanol (5 mL, 2 times), and Fe_3_O_4_@SiO_2_-Pr-DEA-[NTA-Cu(ii)]_2_ was obtained as a grey powder after drying at 60 °C under reduced pressure.

### General procedure of the Buchwald–Hartwig C–N bond formation reaction between aryl halides (1) and *N*-containing compounds (2) in the presence of Fe_3_O_4_@SiO_2_-Pr-DEA-[NTA-Cu(ii)]_2_ (4)

A mixture of *N*-containing compound 2 (1.2 mmol), aryl halide 1 (1 mmol), Cs_2_CO_3_ (2 mmol, 0.65 g), and DMF (3 mL) was stirred in the presence of nanomagnetic catalyst 4 (0.05 g) at 110 °C, and the progress of the reaction was monitored by TLC. After completion of the reaction, the catalyst was separated using an external magnet, washed with EtOH (5 mL, 2 times), dried at 70 °C for 12 h, and reused. The residual solution was immediately poured into saturated brine (10 mL) and extracted with EtOAc (10 mL, 3 times). The combined organic layer was dried over anhydrous Na_2_SO_4_ and concentrated under reduced pressure. The residue was purified by column chromatography on silica gel using a petroleum ether/ethyl acetate (5 : 1 v/v) mixture as the eluent to afford the pure product.

## Results and discussion

**Scheme 2:**
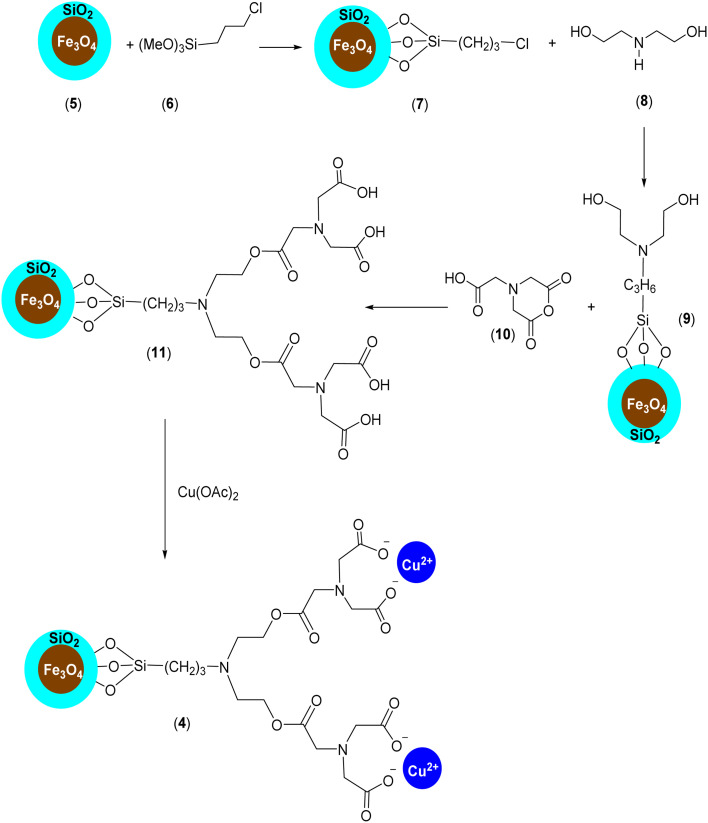
The preparation of Fe_3_O_4_@SiO_2_-Pr-DEA-[NTA-Cu(ii)]_2_ (4) as a new magnetically separable nanocatalyst.

Firstly, our study is focused on the synthesis of an NTA complex of Cu(ii) supported on silica-coated nanosized magnetite 4 (Fe_3_O_4_@SiO_2_-Pr-DEA-[NTA-Cu(ii)]_2_) As demonstrated in Scheme 2, in the first step, the *n*-propylchloride-functionalized silica-coated MNPs 7 (Fe_3_O_4_@SiO_2_-Pr-Cl) were prepared by the treatment of silica-coated nanosized magnetite 5 (Fe_3_O_4_@SiO_2_) with 3-chloropropyl) trimethoxysilane (6). Subsequently, the treatment of freshly synthesized NPs 7 with diethanolamine (DEA) (8) led to the formation of 2,2′-((3-propyl)azanediyl)bis(ethan-1-ol)-functionalized silica-coated MNPs (Fe_3_O_4_@SiO_2_-Pr-DEA) (9), which were reacted with 2-(2,6-dioxomorpholino)acetic acid (10) to prepare 3,15-bis(carboxymethyl)-5,13-dioxo-9-(3-propyl)-6,12-dioxa-3,9,15-triazaheptadecanedioic acid-functionalized silica-coated MNPs (Fe_3_O_4_@SiO_2_-Pr-DEA-[NTA]_2_) (11). Finally, the desired catalyst was obtained by the treatment of 11 with cupper acetate (Scheme 2).

The chemical properties and physical structure of the synthesized nanocatalyst were studied by Fourier transform infrared spectroscopy (FT-IR), X-ray diffraction analysis (XRD), dynamic light scattering (DLS), field-emission scanning electron microscopy (FE-SEM), transmission electron microscopy (TEM), thermogravimetric analysis (TGA), inductively coupled plasma (ICP), vibrating sample magnetometer (VSM), energy dispersive X-ray analysis (EDX) and ultraviolet-visible spectroscopy (UV-vis).

**Fig. 1:**
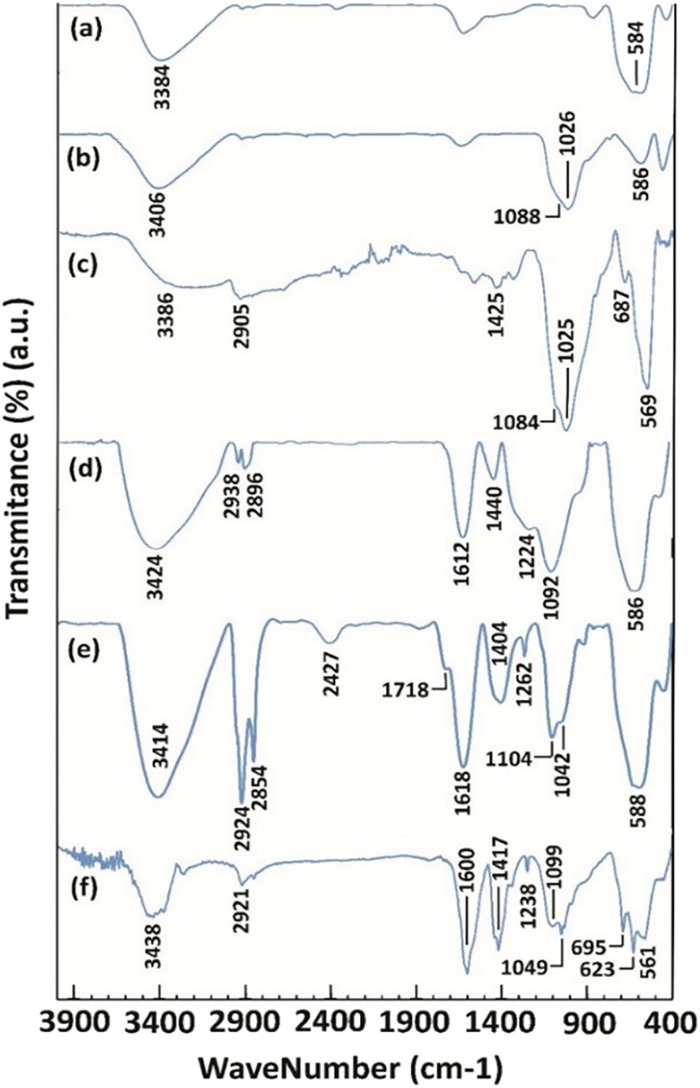
The FT-IR spectra of freshly synthesized (a) Fe_3_O_4_ NPs, (b) Fe_3_O_4_@SiO_2_ NPs (5), (c) Fe_3_O_4_@SiO_2_-Pr-Cl NPs (7), (d) Fe_3_O_4_@SiO_2_-Pr-DEA NPs (9), (e) Fe_3_O_4_@SiO_2_-Pr-DEA-[NTA]_2_ NPs (11) and (f) Fe_3_O_4_@SiO_2_-Pr-DEA-[NTA-Cu(ii)]_2_ NPs (4).

The FT-IR spectra of the synthesized magnetite NPs are presented in Fig. 1. In the FT-IR spectrum of magnetite NPs (Fig. 1, curve a), peaks were found at 584 and 3384 cm^−1^ corresponding to the stretching vibrations of Fe–O and the hydroxyl groups of Fe_3_O_4_ NPs, respectively. The presence of Fe–O stretching vibration in all the other spectra clarifies the existence and stability of the magnetite NPs in all the other NMPs prepared (Fig. 1, curves b, c, d, e, and f). In the FT-IR spectrum of Fe_3_O_4_@SiO_2_ (5), the peaks at 1026 and 1088 cm^−1^ belong to the Si–O stretching vibrations (Fig. 1, curve b). The stretching vibrations of Si–O bonds were also observed in the FT-IR spectra of 7, 9, 11 and 4 NPs at 1025 and 1084, 1092, 1104, and 1049 cm^−1^, respectively (Fig. 1, curves c, d, e, and f). The FT-IR spectrum of 7 contained the C–Cl stretching, CH_2_ bending and sp^3^ C–H stretching vibration peaks at 687, 1425, and 2905 cm^−1^, respectively, as expected (Fig. 1, curve c). The disappearance of the C–Cl stretching peak and the presence of the C–N stretching, CH_2_ bending and O–H bending peaks at 1224, 1440, and 1612 cm^−1^, respectively (Fig. 1, curve d) prove the successful synthesis of NPs 9. The FT-IR spectrum of 11 contained the O=C–O–C̲H_2_ stretching, C–N stretching, CH_2_ bending, carboxylic acid C=O stretching, carboxylic acid O–H stretching and sp^3^ C–H stretching peaks at 1042, 1262, 1404, 1718, 2427 (broad peak), 2854 and 2924 cm^−1^, respectively (Fig. 1, curve e). The disappearance of carboxylic acid O–H stretching and the presence of the O=C–O^−1^ twisting, O=C–O^−1^ scissoring, C–N stretching, O=C–O^−1^ symmetric stretching, O=C–O^−1^ asymmetric stretching, and sp^3^ C–H stretching signals at 623,^25^ 695,^26^ 1238, 1417, 1600, and 2921 cm^−1^, respectively (Fig. 1, curve f), prove the successful formation of the NTA complex of Cu(ii) supported on silica-coated nanosized magnetite 4. In the obtained spectra, peaks at 3384 (Fig. 1, curve a), 3406 (Fig. 1, curve b), 3386 (Fig. 1, curve c), 3424 (Fig. 1, curve d), 3414 (Fig. 1, curve e) and 3438 cm^−1^ (Fig. 1, curve f) belonging to O–H stretching vibrations were observed.

**Fig. 2:**
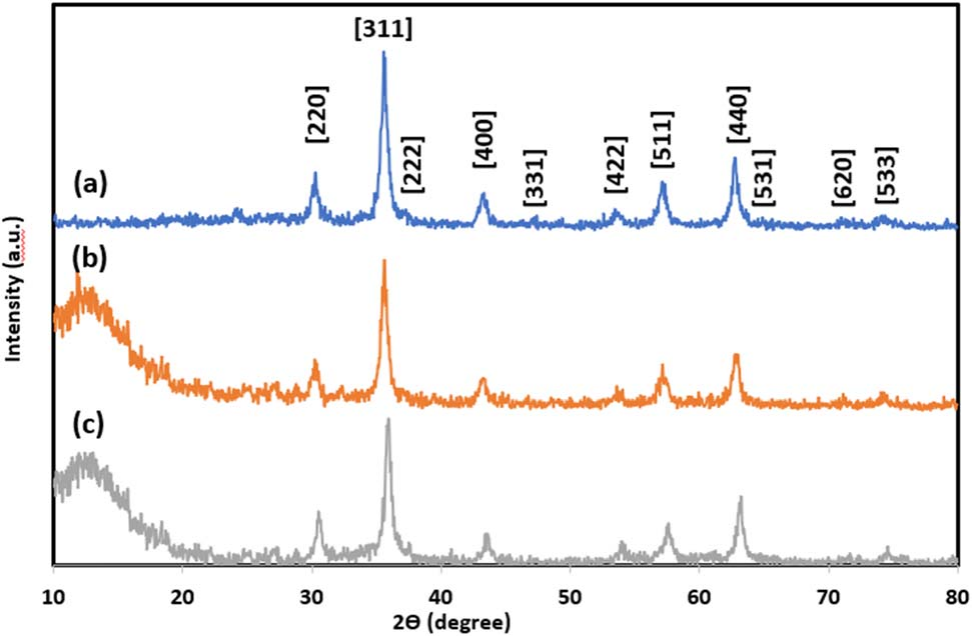
The XRD patterns of (a) Fe_3_O_4_, (b) Fe_3_O_4_@SiO_2_, and (c) Fe_3_O_4_@SiO_2_-Pr-DEA-[NTA-Cu(ii)]_2_ NPs.

The XRD patterns of magnetite NPs, silica-coated nanosized magnetite (5), and the NTA complex of Cu(ii) supported on silica-coated nanosized magnetite (4) are presented in Fig. 2a–2c, respectively. The peaks at 2*θ* = 30.3°, 35.6°, 37.3°, 43.5°, 47.2°, 53.8°, 57.2°, 62.7°, 64.9°, 71°, and 75.5° corresponding to the Bragg reflections of [220], [311], [222], [400], [331], [422], [511], [440], [531], [620], and [533], respectively, indicate the cubic spinel structure of magnetite NPs and comply with the XRD spectrum of standard magnetite (JCPDS Card No. 19-629) (Fig. 2a). A broad peak was found between 2*θ* = 10–20° in the XRD pattern of silica-coated nanosized magnetite (Fe_3_O_4_@SiO_2_) corresponding to the amorphous silica shell around the Fe_3_O_4_ NPs (Fig. 2b).

**Fig. 3:**
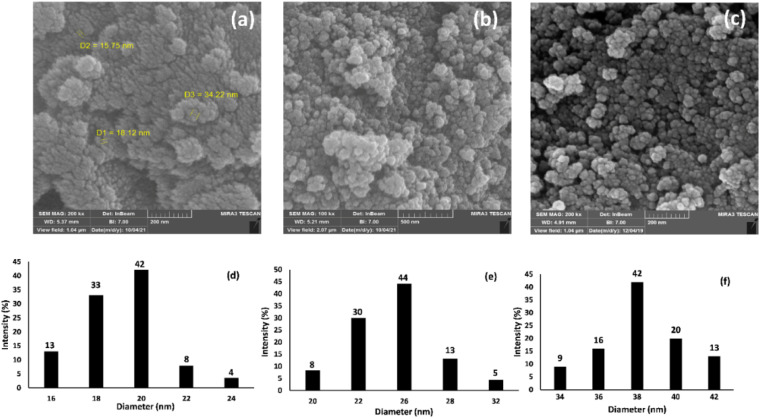
The FE-SEM images of (a) Fe_3_O_4_ NPs, (b) Fe_3_O_4_@SiO_2_ NPs, and (c) Fe_3_O_4_@SiO_2_-Pr-DEA-[NTA-Cu(ii)]_2_ NPs, and the dynamic light scattering (DLS) analysis of (d) Fe_3_O_4_ NPs, (e) Fe_3_O_4_@SiO_2_ NPs, and (f) Fe_3_O_4_@SiO_2_-Pr-DEA-[NTA-Cu(ii)]_2_ NPs.

The same reflections were observed at lower intensities in the XRD pattern of the Fe_3_O_4_@SiO_2_-Pr-DEA-[NTA-Cu(ii)]_2_ NPs due to the presence of organic compounds chemically bonded to the surface of amorphous silica (Fig. 3c). All these data confirm the successful preparation of a core–shell system with the Fe_3_O_4_ NPs core coated by the SiO_2_ shell. Using the Debye–Scherrer equation, the crystal size of the magnetite nanocore (D) was calculated to be around 16 nm (when *K* = 0.94, *λ* = 1.5406 Å).

**Fig. 4:**
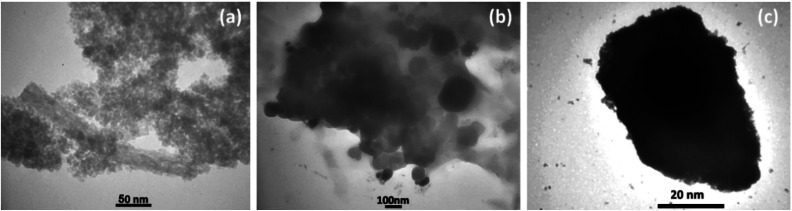
TEM images of (a) Fe_3_O_4_ NPs, (b) Fe_3_O_4_@SiO_2_ NPs, and (c) Fe_3_O_4_@SiO_2_-Pr-DEA-[NTA-Cu(ii)]_2_ NPs.

The morphology and particle sizes of the synthesized Fe_3_O_4_ NPs, Fe_3_O_4_@SiO_2_ NPs, and Fe_3_O_4_@SiO_2_-Pr-DEA-[NTA-Cu(ii)]_2_ NPs were studied by FE-SEM and DLS analyses (Fig. 3a–c and 3d-f, respectively). As shown in Fig. 3a–c, all synthesized NPs showed nearly spherical shapes. The particle sizes of Fe_3_O_4_ NPs were highly distributed around 20 nm (Fig. 3d), which corresponds with the value obtained from the XRD pattern. The particle sizes of Fe_3_O_4_@SiO_2_ NPs and Fe_3_O_4_@SiO_2_-Pr-DEA-[NTA-Cu(ii)]_2_ NPs were distributed around 26 and 38 nm, respectively (Fig. 3e and f). Moreover, according to the DLS analysis, the size of the NPs increased with each step of synthesis of the catalyst, as expected. The TEM images of Fe_3_O_4_ NPs, Fe_3_O_4_@SiO_2_ NPs, and Fe_3_O_4_@SiO_2_-Pr-DEA-[NTA-Cu(ii)]_2_ NPs shown respectively in Fig. 4a–c exhibit the nearly spherical shapes of all synthesized NPs and the core–shell pattern of the synthesized catalyst.

**Fig. 5:**
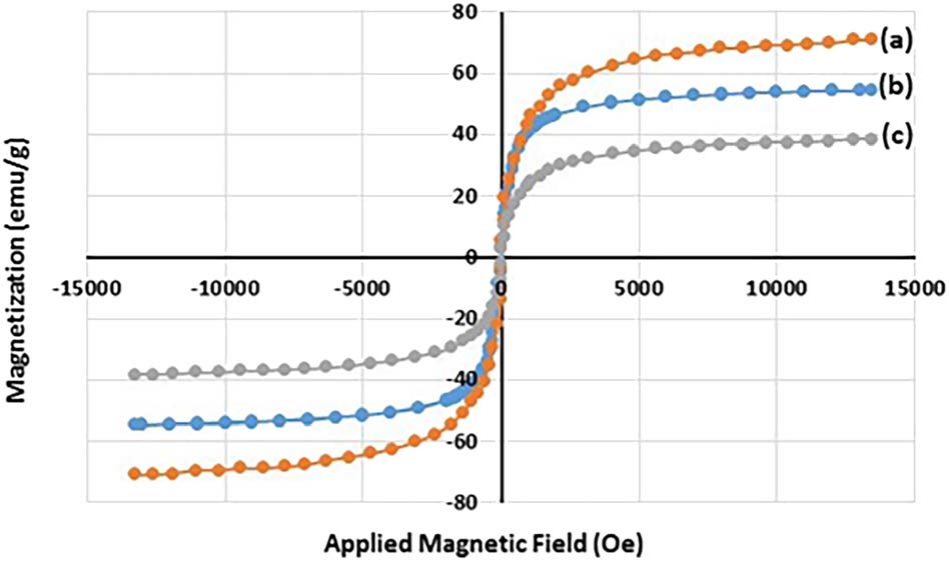
The VSM curves of (a) Fe_3_O_4_ NPs, (b) Fe_3_O_4_@SiO_2_ NPs, and (c) Fe_3_O_4_@SiO_2_-Pr-DEA-[NTA-Cu(ii)]_2_ NPs at 300 K.

The magnetization properties of the synthesized nanocatalyst were studied using a vibrating sample magnetometer (VSM). The VSM curves of Fe_3_O_4_ NPs, Fe_3_O_4_@SiO_2_ NPs, and freshly synthesized Fe_3_O_4_@SiO_2_-Pr-DEA-[NTA-Cu(ii)]_2_ NPs are shown in Fig. 5a–c, respectively. Notably, in a superparamagnetic material, without any external magnetic field (*H* = 0), the magnetic vectors of each magnetic particle are randomly placed in different directions and their total result is zero.^27^ As shown in Fig. 5, in the VSM curves of all three samples examined, no hysteresis loop or remanence (*M*_r300K_ = 0) was detected at 300 K. Moreover, the coercivity value was zero (*H*_C300K_ = 0) for all samples; these data suggest the superparamagnetic behaviour of the studied samples. The saturation magnetization values were found to be 70.8, 54.4, and 38.3 emu g^−1^ for the Fe_3_O_4_ NPs, Fe_3_O_4_@SiO_2_ NPs, and Fe_3_O_4_@SiO_2_-Pr-DEA-[NTA-Cu(ii)]_2_ NPs, respectively. The reduction in the saturation magnetization value of the Fe_3_O_4_@SiO_2_ NPs and Fe_3_O_4_@SiO_2_-Pr-DEA-[NTA-Cu(ii)]_2_ NPs compared with the Fe_3_O_4_ NPs is due to relatively less magnetization per unit mass, which stems from the addition of a silica shell and organic parts to the central Fe_3_O_4_ nanocores.

**Fig. 6:**
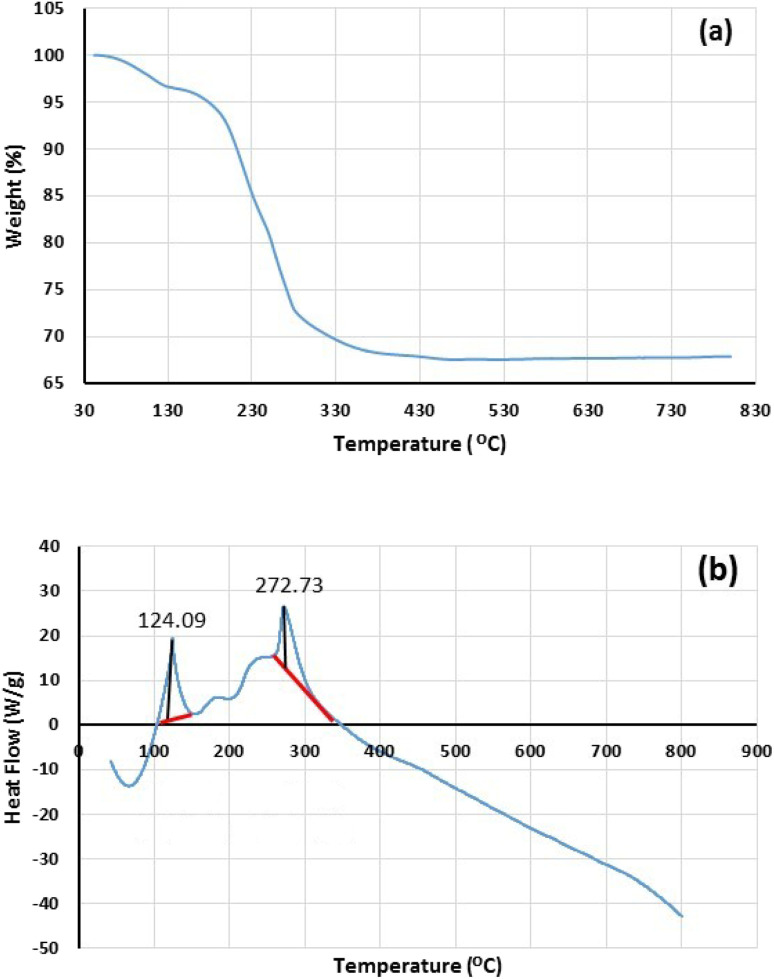
The (a) TG and (b) DSC analyses of freshly synthesized Fe_3_O_4_@SiO_2_-Pr-DEA-[NTA-Cu(ii)]_2_ NPs.

The thermal stability of freshly synthesized Fe_3_O_4_@SiO_2_-Pr-DEA-[NTA-Cu(ii)]_2_ NPs was also investigated by TGA-DSC analysis, and the obtained results are demonstrated in Fig. 6. Two major weight loss stages were found in the thermogram of Fe_3_O_4_@SiO_2_-Pr-DEA-[NTA-Cu(ii)]_2_ NPs at 90–115 °C (3.4%) and 180–450 °C (28.7%), which can be related to the desorption of water vapor and other volatile organic compounds adsorbed on the catalyst and the loss of covalently bonded organic groups, respectively (Fig. 6a). Based on the results obtained from the thermogram, the content of organic moieties was about 28.7% against the solid support and other inorganic materials. Considering the data obtained from TGA, the loading organic ligand was calculated to be approximately 0.59 mmol per gram of synthesized catalyst. Moreover, DSC analysis was carried out in the range of 50–800 °C under an N_2_ atmosphere at 10 °C min^−1^, which showed two endothermic peaks at 124.09 and 272.73 °C (Fig. 6b). The results of DSC confirm the TGA results of Fe_3_O_4_@SiO_2_-Pr-DEA-[NTA-Cu(ii)]_2_ NPs.

**Fig. 7:**
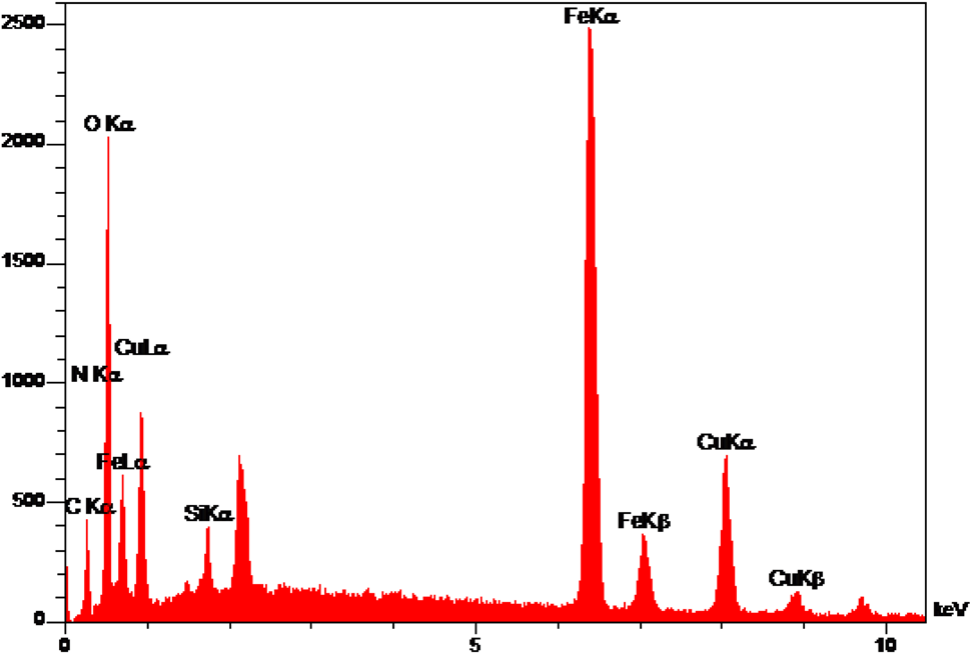
The EDX analysis of Fe_3_O_4_@SiO_2_-Pr-DEA-[NTA-Cu(ii)]_2_ NPs.

**Fig. 8:**
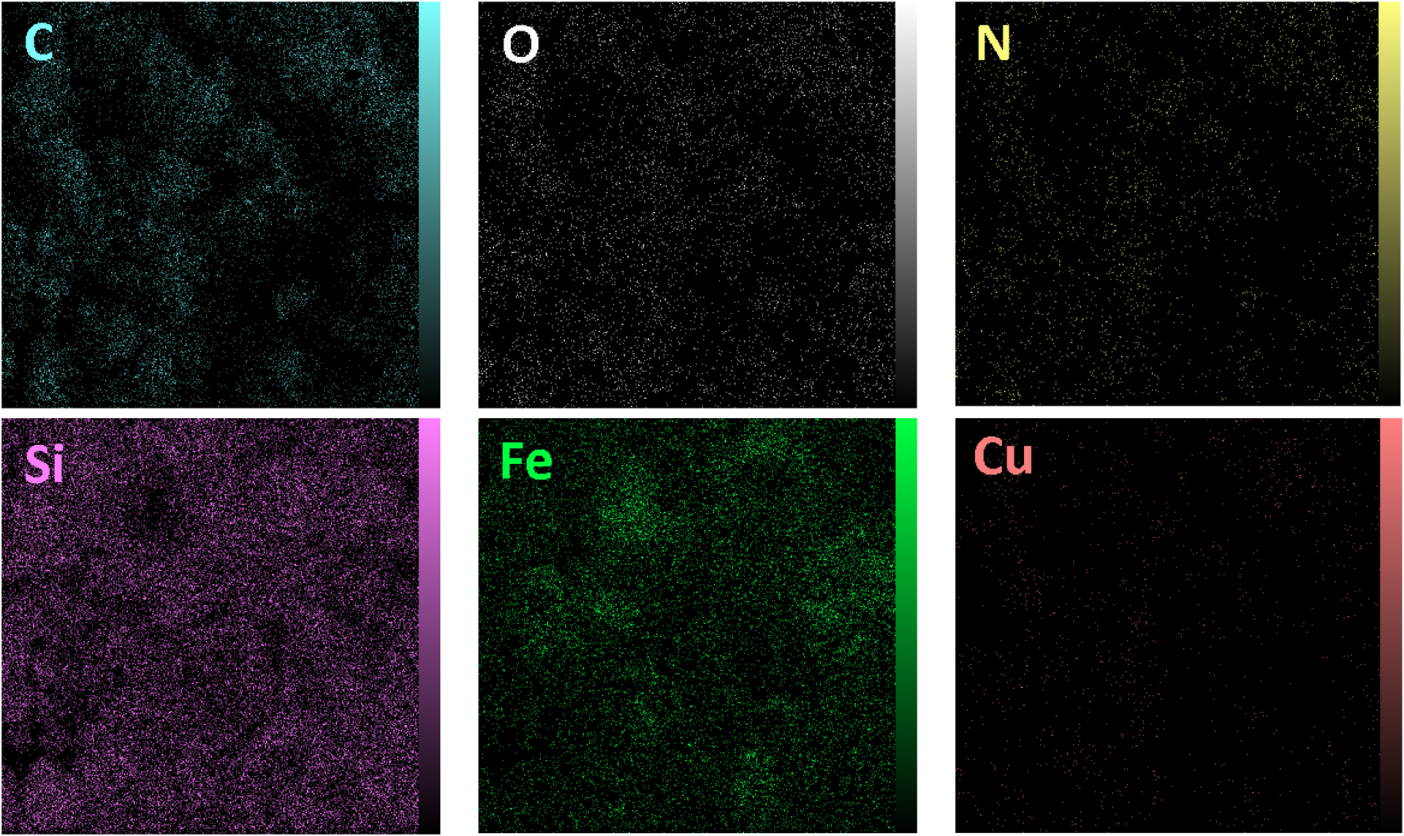
The FESEM-EDX map analysis of Fe_3_O_4_@SiO_2_-Pr-DEA-[NTA-Cu(ii)]_2_ NPs.

The elemental analysis of freshly synthesized Fe_3_O_4_@SiO_2_-Pr-DEA-[NTA-Cu(ii)]_2_ NPs was conducted by energy dispersive spectroscopy (EDX), FESEM, EDX elemental mapping, and ICP-OES analyses. Based on the EDX spectra (Fig. 7), the Fe_3_O_4_@SiO_2_-Pr-DEA-[NTA-Cu(ii)]_2_ NPs consisted of C, N, O, Fe, Cu, and Si. Moreover, the FESEM-EDX mapping analysis determined the uniform distribution of elements in the catalyst structure (Fig. 8).

ICP-OES was used for the determination of copper content in the freshly synthesized catalyst. Using this method, the actual Cu content of Fe_3_O_4_@SiO_2_-Pr-DEA-[NTA-Cu(ii)]_2_ NPs was measured to be 1.28 mmol of Cu per gram of the catalyst, which is in good agreement with the data obtained from the TG analysis and the organic ligand content measured in the catalyst.

**Fig. 9:**
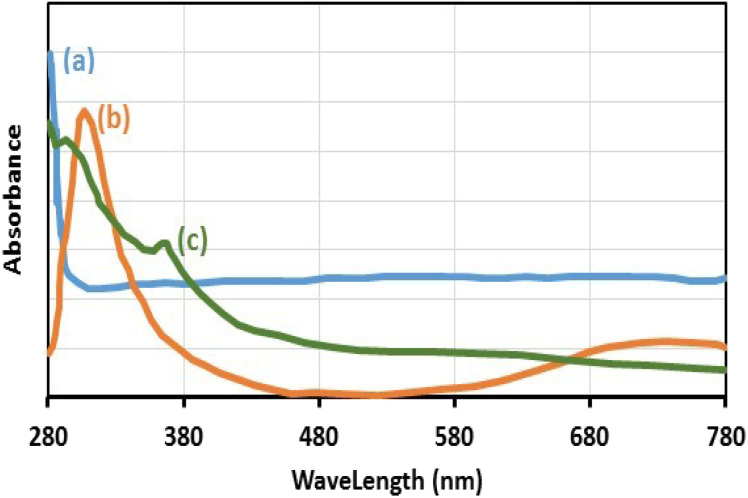
The solid-state UV-vis spectra of (a) Fe_3_O_4_ NPs, (b) Cu(OAc)_2_, and (c) Fe_3_O_4_@SiO_2_-Pr-DEA-[NTA-Cu(ii)]_2_ NPs.

The existence of Cu(ii) in the chemical structure of Fe_3_O_4_@SiO_2_-Pr-DEA-[NTA-Cu(ii)]_2_ NPs was also investigated by solid-state UV-vis spectroscopy, and the obtained spectra of Fe_3_O_4_ NPs, Cu(OAc)_2,_ and freshly synthesized Fe_3_O_4_@SiO_2_-Pr-DEA-[NTA-Cu(ii)]_2_ NPs are presented in Fig. 9a–c, respectively. In the UV-vis spectrum of Fe_3_O_4_@SiO_2_-Pr-DEA-[NTA-Cu(ii)]_2_ NPs, the absorption bands at around 295 and 367 nm are representative of the Cu^2+^ species.^28^

**Fig. 10:**
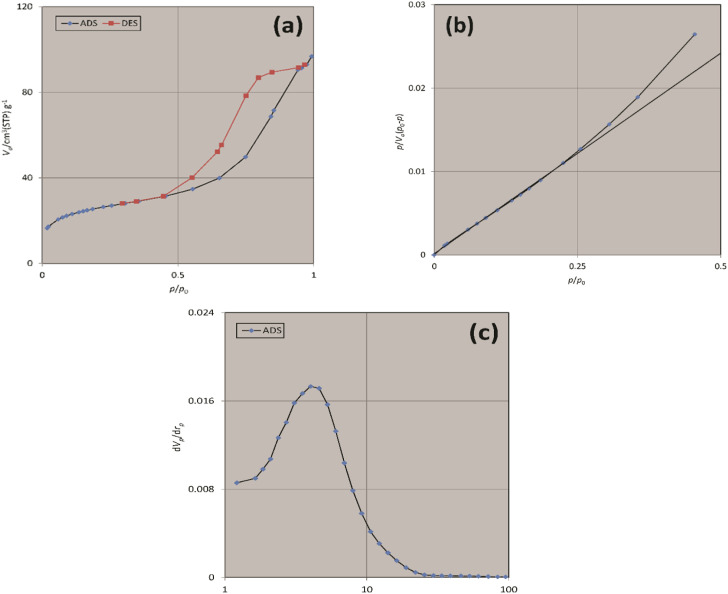
The (a) N_2_ adsorption–desorption, (b) BET, and (c) BJH isotherms of freshly synthesized Fe_3_O_4_@SiO_2_-Pr-DEA-[NTA-Cu(ii)]_2_ NPs.

The Brunauer–Emmett–Teller (BET) analysis was used to study the porous structure and surface area of freshly synthesized Fe_3_O_4_@SiO_2_-Pr-DEA-[NTA-Cu(ii)]_2_ NPs, and the obtained results are summarized in Table 1 and Fig. 10. The measured specific surface area was 90.21 m^2^ g^−1^ with a total pore volume of 0.1488 cm^3^ g^−1^ and a mean pore diameter of 6.5994 nm.

**Table 1:** The BET analysis results of freshly synthesized NTAC-Cu(ii)-SSCNM NPs

Sample	BET surface area (m^2^ g^−1^)	Total pore volume (cm^3^ g^−1^)	Mean pore diameters (nm)
Fe_3_O_4_@SiO_2_-Pr-DEA-[NTA-Cu(ii)]_2_ NPs	90.21	0.1488	6.5994

After the successful preparation and characterization of Fe_3_O_4_@SiO_2_-Pr-DEA-[NTA-Cu(ii)]_2_ NPs as a new nanomagnetic catalyst, its catalytic activity in the Buchwald–Hartwig C–N bond formation reaction was studied (Scheme 1). For this, the reaction of imidazole (2a, 1.2 mmol) and iodobenzene (1a, 1 mmol) was selected as the model reaction (Scheme 1), and the time and the yield of the reaction were monitored under different conditions, such as solvent, temperature, base and the amount of catalyst, and the obtained results are summarized in Table 2. Based on the obtained results, it is obvious that the base, solvent, and catalyst play crucial roles in promoting the studied reaction. As seen in Table 2, moderate to good yields were obtained in DMF, DMSO, NMP, MeCN, and toluene (Table 2, entries 1, 2, 4, 8, and 9), while the reactions carried out in DME, H_2_O, EtOH, and MeOH were not effective, and very low yields were obtained after a long time (12 h) (Table 2, entries 3, 5, 6 and 7). The best results were obtained when DMF was used as the solvent (Table 2, entry 1). In addition, the reaction temperature directly affected the yield and time of the reaction. The shortest reaction time (1.5 h) and the best reaction yield (93%) were obtained at 110 °C (Table 1, entry 1).

**Table 2:** Optimization of the reaction parameters for the production of 1-phenylimidazole (3a) in the presence of Fe_3_O_4_@SiO_2_-Pr-DEA-[NTA-Cu(ii)]_2_ NPs^a^

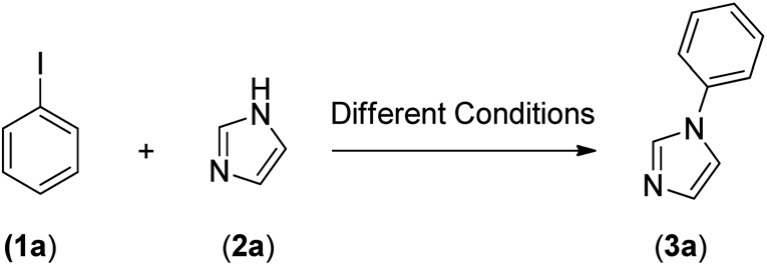						
Entry	Catalyst amount (g)	Solvent (mL)	Base (mmol)	Temperature (°C)	Time (h)	Yield^b^ (%)
1	0.05	DMF	Cs_2_CO_3_	110	1.5	93
2	0.05	DMSO	Cs_2_CO_3_	110	4	92
3	0.05	DME	Cs_2_CO_3_	Reflux	12	40
4	0.05	NMP	Cs_2_CO_3_	110	4	93
5	0.05	H_2_O	Cs_2_CO_3_	Reflux	12	Trace
6	0.05	EtOH	Cs_2_CO_3_	Reflux	12	Trace
7	0.05	MeOH	Cs_2_CO_3_	Reflux	12	Trace
8	0.05	MeCN	Cs_2_CO_3_	Reflux	9	85
9	0.05	Toluene	Cs_2_CO_3_	Reflux	9	89
10	0.05	DMF	Cs_2_CO_3_	140	1.5	93
11	0.05	DMF	Cs_2_CO_3_	130	1.5	92
12	0.05	DMF	Cs_2_CO_3_	120	1.5	92
13	0.05	DMF	Cs_2_CO_3_	100	2.5	92
14	0.05	DMF	Cs_2_CO_3_	90	4.5	90
15	0.07	DMF	Cs_2_CO_3_	110	1.5	93
16	0.09	DMF	Cs_2_CO_3_	110	1.5	93
17	0.03	DMF	Cs_2_CO_3_	110	4.5	92
18	0.01	DMF	Cs_2_CO_3_	110	12	83
19	0.05	DMF	NaOH	110	12	54
20	0.05	DMF	K_3_PO_4_	110	12	72
21	0.05	DMF	NaOAc	110	12	48
22	0.05	DMF	K_2_CO_3_	110	12	66
23	—	DMF	Cs_2_CO_3_	110	12	N.R.

a Reaction conditions: iodobenzene (1 mmol), imidazole (1.2 mmol), catalyst (g), solvent (3 mL), Base (2 mmol), temperature (^o^C), time (h).

b Isolated yields.

Increasing the temperature up to 140 °C had no significant effect on the time and yield of the reaction (Table 2, entries 10, 11, and 12). However, lowering the temperature significantly increased the reaction time and decreased the yield (Table 2, entries 13 and 14).

As the results indicate, the reaction was highly sensitive to the presence of the catalyst and did not proceed without the catalyst even after a long time (12 h) (Table 2, entry 23). The best results were obtained using 0.05 g of the catalyst (Table 2, entry 1), and increasing the catalyst quantity did not have a significant effect on the reaction time and yield (Table 2, entries 15 and 16), whereas, a decrease in the quantity of catalyst led to a significant increase in reaction time and decrease in yield (Table 2, entries 17 and 18). The effect of different bases, including Cs_2_CO_3_ (Table 2, entry 1), NaOH (Table 2, entry 19), K_3_PO_4_ (Table 2, entry 20), NaOAc (Table 2, entry 21), and K_2_CO_3_ (Table 2, entry 22), on the reaction was also studied, and the best results were obtained with Cs_2_CO_3_ (Table 2, entry 1). Therefore, considering all these results, the best reaction conditions for the reaction of iodobenzene (1a, 1 mmol) and imidazole (2a, 1.2 mmol) in the presence of Fe_3_O_4_@SiO_2_-Pr-DEA-[NTA-Cu(ii)]_2_ were: DMF (3 mL) as the solvent, 0.05 gram of the catalyst, Cs_2_CO_3_ (2 mmol, 0.65 g) as the base and a reaction temperature of 110 °C.

Under the optimized reaction conditions, the versatility and generality of Fe_3_O_4_@SiO_2_-Pr-DEA-[NTA-Cu(ii)]_2_ as a new nanomagnetic nanocatalyst were investigated, and the obtained results are summarized in Table 3. As shown in Table 3, various kinds of nitrogen heterocycles, such as imidazole (Table 3, entries 1–6), 2-methyl-1*H*-imidazole (Table 3, entries 7, 8, and 9), benzimidazole (Table 3, entries 10, 11, and 12), indole (Table 3, entries 13, 14, 15 and 16) and 10*H*-phenothiazine (Table 3 entries 17, 18 and 19), were applied successfully, and the desired products were obtained with good to excellent yields. Moreover, some secondary aliphatic amines, such as piperidine (Table 3, entries 20, 21, and 22), piperazine (Table 3, entry 23), morpholine (Table 3, entries 24 and 25), dimethylamine (Table 3, entries 26 and 27) and diethylamine (Table 3, entry 28), were tested. And the desired products were formed with good yields. The effect of aryl halide on the efficiency of the developed method was also investigated. The above-mentioned NH-containing compounds were reacted with aryl iodide, aryl bromide, and aryl chloride in the presence of NTAC-Cu(ii)-SSCNM NPs under optimized reaction conditions, and the obtained results are summarized in Table 3. As seen in Table 3, the efficiency of the as-developed method is highly sensitive to C-X (X: halide) bond reactivity; a decrease in reaction yield and an increase in reaction time were observed with the change of halide from iodide to bromide and chloride. With aryl iodides, the reactions occurred in relatively shorter durations (1–8 h), and the desired products were obtained with good to excellent yields (80–95%). With aryl bromide, although the reaction time increased (3–15 h), the yield of desired products was still good (80–92%). However, with aryl chloride, the reaction yields were greatly reduced; even after a very long time (24 h), only small amounts of desired products were obtained (24–40%). Another factor that affects the reaction time and yield is the presence of electron-withdrawing and -donating substituents on the aromatic ring of the applied aryl halides. As seen from the results in Table 3, the presence of electron-withdrawing groups on the aromatic rings of aryl halides decreased the reaction time and increased the reaction yield (Table 3, entries 2, 8, 10 and 14), while aryl halides with electron donor groups presented increased reaction time and decreased reaction yields (Table 3, entries 3, 11, 12, 15, 16 and 19).

**Table 3:** The Buchwald–Hartwig C–N bond formation reaction of aryl halides and NH-containing compounds in the presence of Fe_3_O_4_@SiO_2_-Pr-DEA-[NTA-Cu(ii)]_2_ NPs^a^

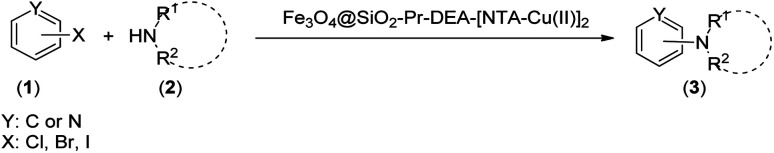					
Entry	X	Y	Product	Time (h)	Yield^d^ (%)
1	I	C	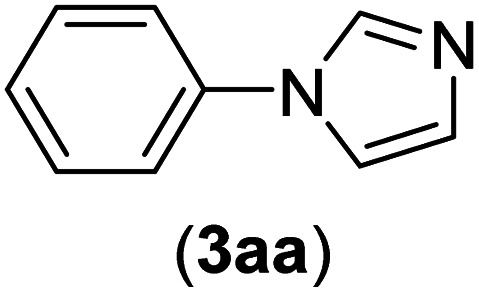	1.5	93
	Br			4	90
	Cl			24	35
2	I	C	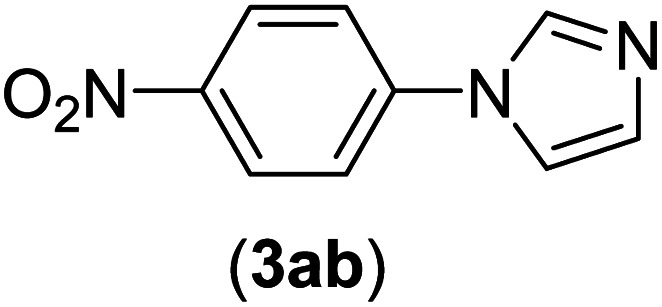	1	95
	Br			3	90
	Cl			24	40
3^b^	I	C	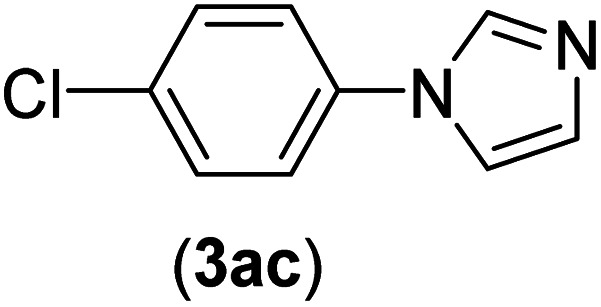	2.5	91
	Br			6	90
	Cl			24	30
4	I	C	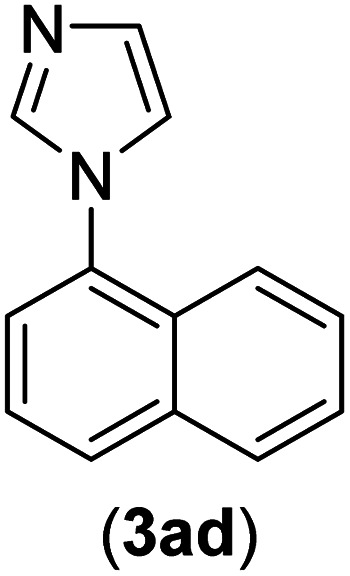	2	91
	Br			5	90
	Cl			24	30
5	I	N	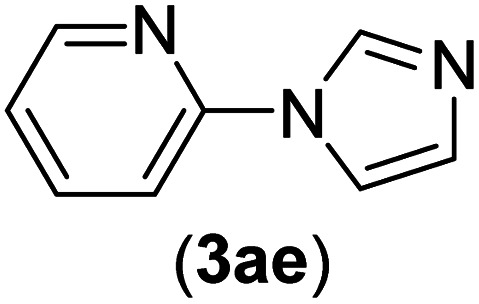	1	90
	Br			4	85
	Cl			24	30
6^b^	I	C	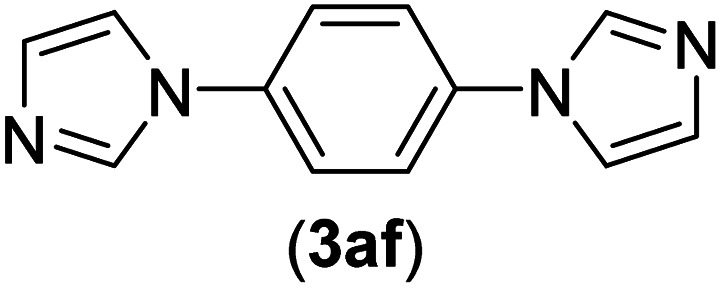	3	88
	Br			7	87
	Cl			24	—
7	I	C	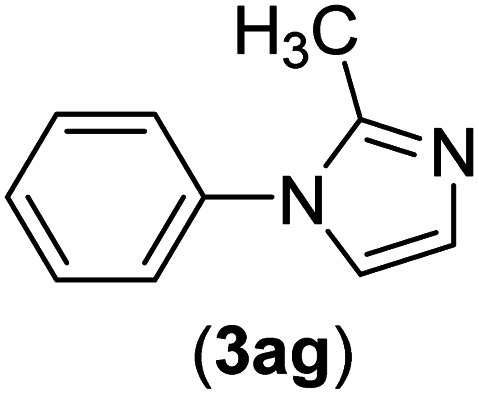	3	91
	Br			6	83
	Cl			24	30
8	I	C	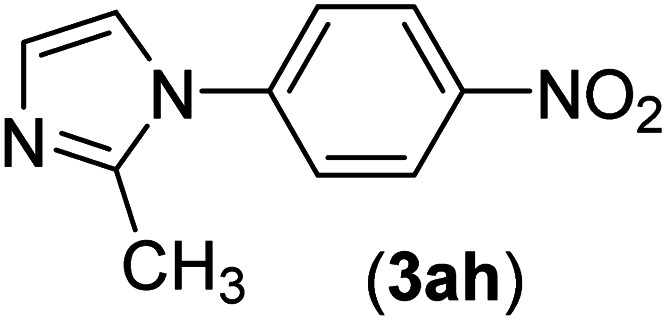	1	92
	Br			3	92
	Cl			24	35
9	I	N	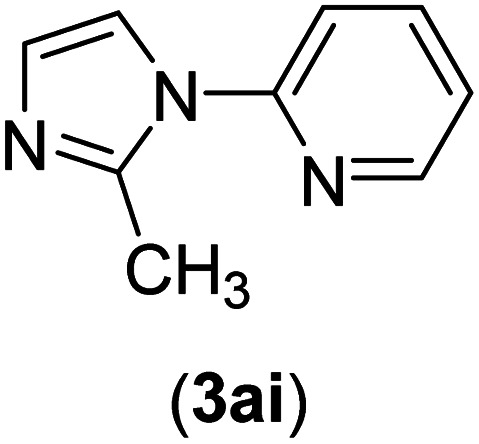	2	90
	Br			5	90
	Cl			24	33
10	I	C	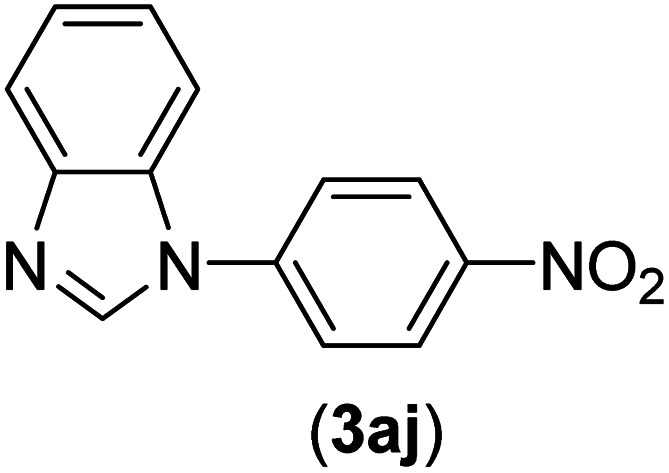	1.5	95
	Br			5	90
	Cl			24	35
11	I	C	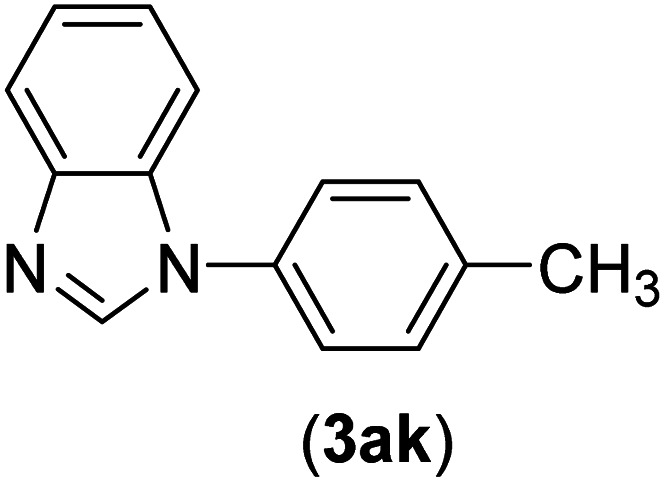	2.5	91
	Br			6	90
	Cl			24	27
12	I	C	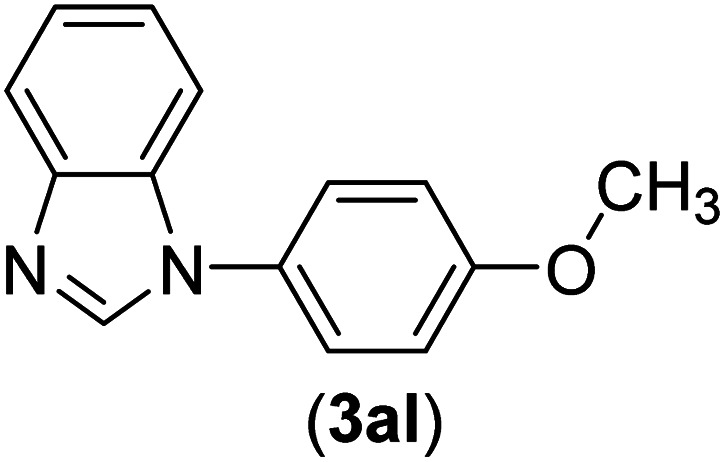	4	83
	Br			9	85
	Cl			24	—
13	I	C	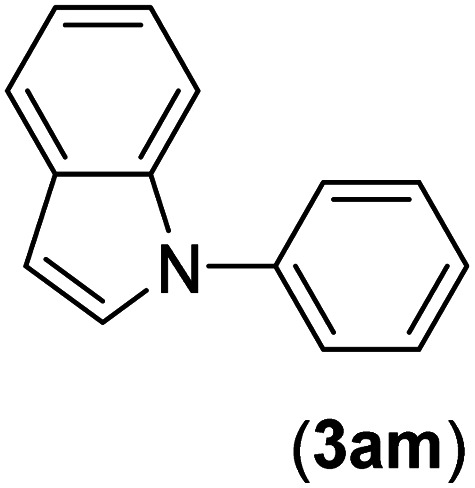	3	87
	Br			7	82
	Cl			24	31
14	I	C	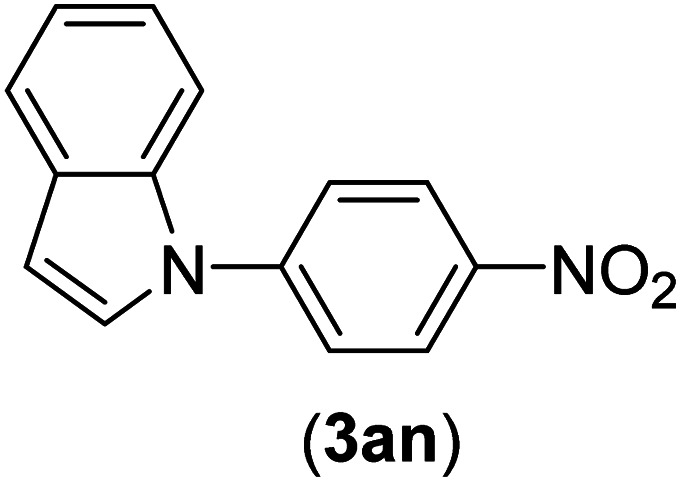	1.5	91
	Br			5	88
	Cl			24	38
15	I	C	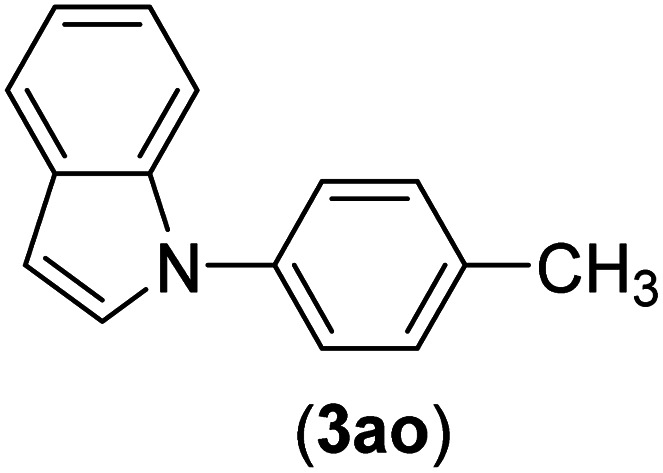	4	85
	Br			9	86
	Cl			24	—
16	I	C	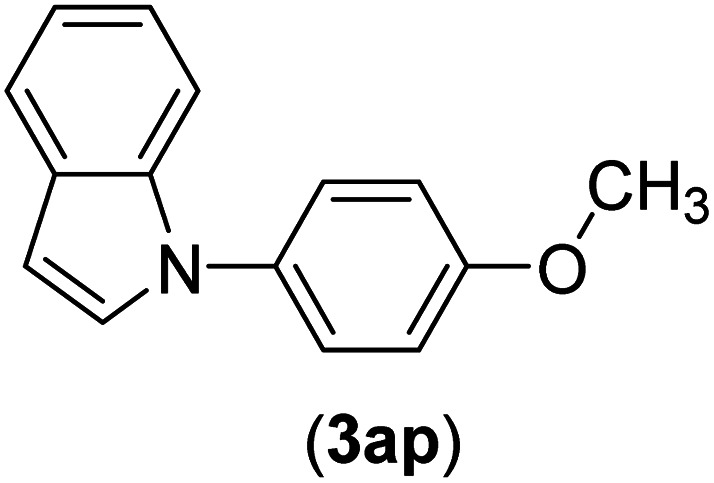	7	85
	Br			11	80
	Cl			24	—
17	I	C	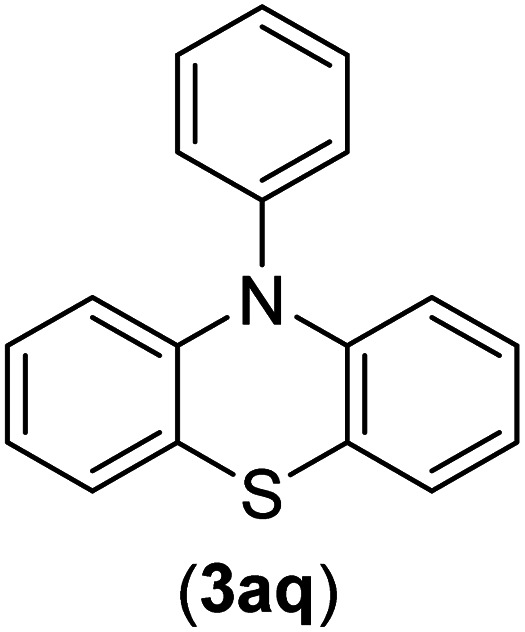	3	90
	Br			7	90
	Cl			24	29
18	I	C	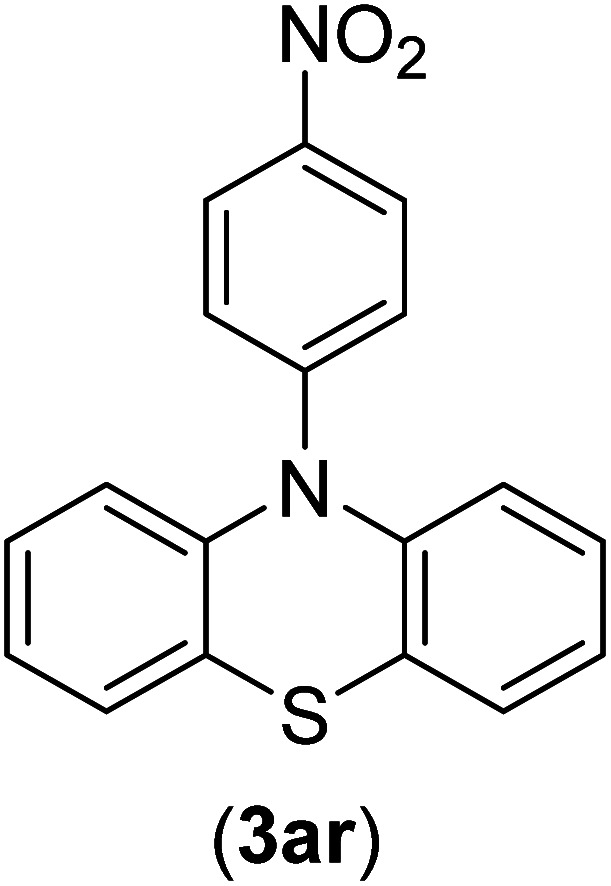	2	90
	Br			5	90
	Cl			24	37
19	I	C	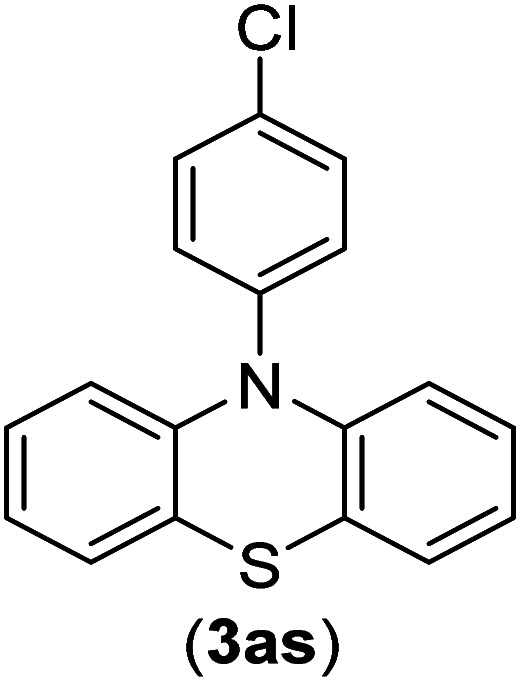	4	91
	Br			7	86
	Cl			—	—
20	I	C	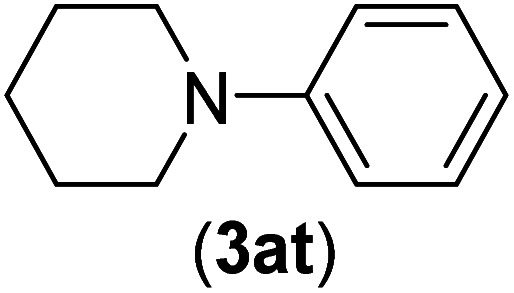	4	81
	Br			7	81
	Cl			24	—
21	I	C	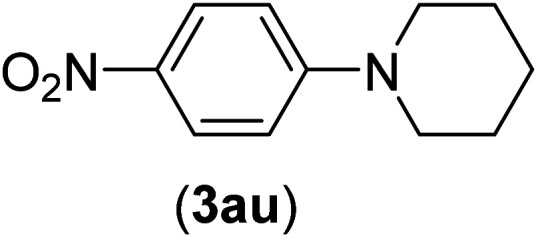	3	88
	Br			6	89
	Cl			24	25
22^b^	I	C	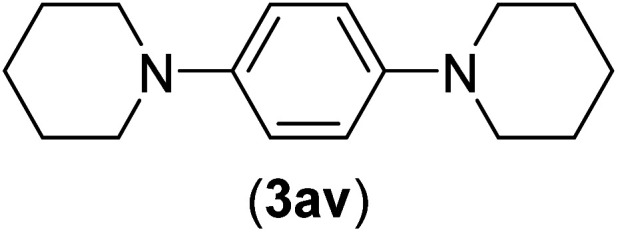	5	91
	Br			9	85
	Cl			24	—
23^c^	I	C	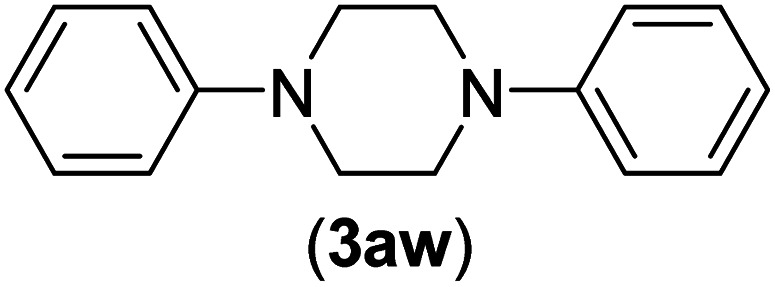	5	81
	Br			10	83
	Cl			24	—
24	I	C	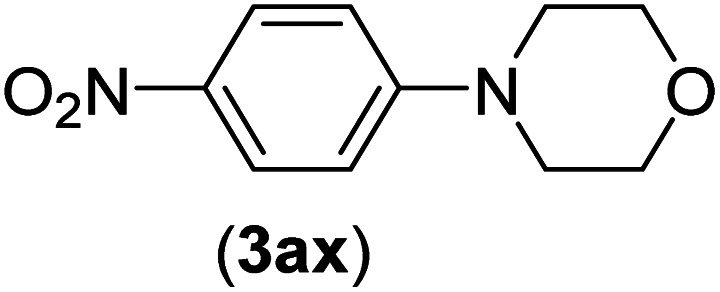	3	86
	Br			7	80
	Cl			24	28
25^b^	I	C	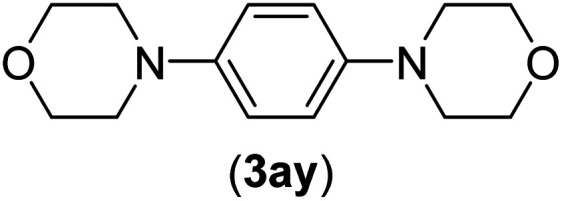	6	83
	Br			12	83
	Cl			24	—
26	I	C	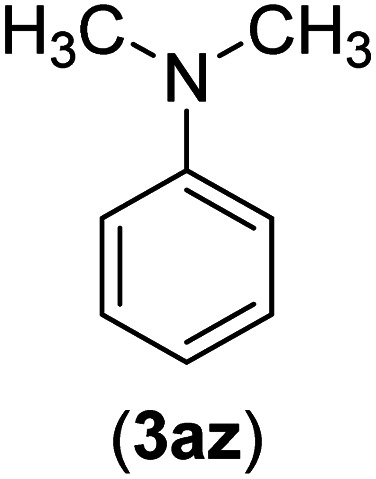	8	80
	Br			14	68
	Cl			24	—
27	I	C	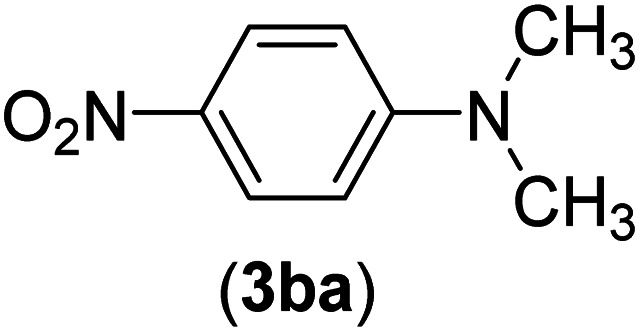	6	86
	Br			14	88
	Cl			24	—
28	I	C	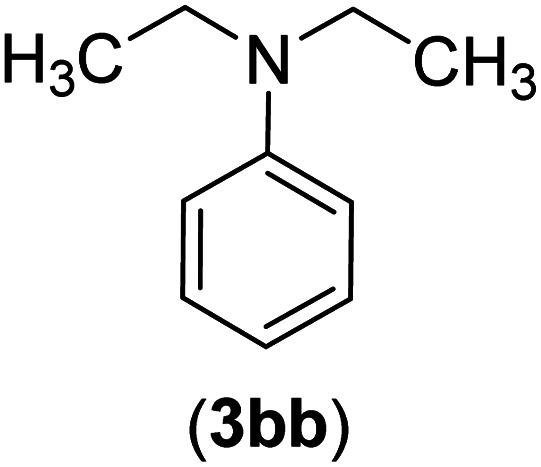	8	83
	Br			15	80
	Cl			24	—

a Reaction conditions: NH-containing compound (1.2 mmol), aryl halide (1 mmol), catalyst (0.05 g), DMF (3 mL), 110 °C.

b 1,4-Dihalobenzene (1 mmol) and secondary aliphatic amine (2.4 mmol).

c Aryl halide (2.1 mmol) and secondary aliphatic amine (1 mmol).

d Isolated yield.

**Scheme 3:**
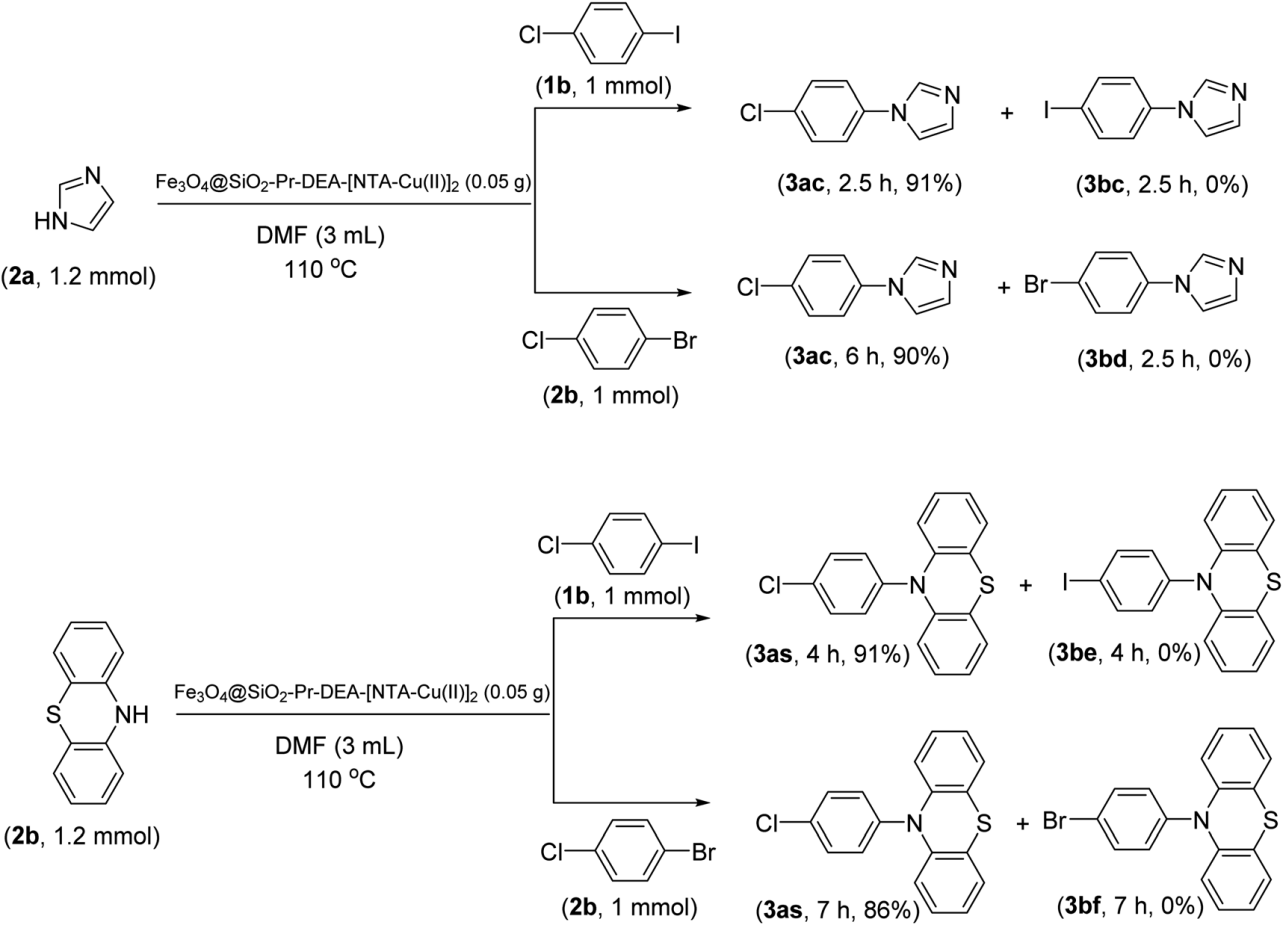
The chemoselectivity analysis of C–N bond formation in the presence of Fe_3_O_4_@SiO_2_-Pr-DEA-[NTA-Cu(ii)]_2_.

The chemoselectivity of the developed method was investigated. For this, imidazole (2a) and 10*H*-phenothiazine (2b) were reacted with 1-chloro-4-iodobenzene (1b) and 1-bromo-4-chloro benzene (2b) in the presence of Fe_3_O_4_@SiO_2_-Pr-DEA-[NTA-Cu(ii)]_2_ NPs under the optimized reaction conditions (Scheme 3). The products of both reactions of imidazole with 1-chloro-4-iodobenzene (1b) and 1-bromo-4-chloro benzene (2b) was 1-(4-chlorophenyl)-1*H*-imidazole (3ac) (Table 3, entry 3), while 1-(4-iodophenyl)-1*H*-imidazole (3bc) and 1-(4-bromophenyl)-1*H*-imidazole (3bd) were not detected at all. Similarly, the only product of both reactions of 10*H*-phenothiazine (2b) with 1-chloro-4-iodobenzene (1b) and 1-bromo-4-chloro benzene (2b) was 10-(4-chlorophenyl)-10*H*-phenothiazine (3as) (Table 3, entry 19), while 10-(4-iodophenyl)-10*H*-phenothiazine (3be) and 10-(4-bromophenyl)-10*H*-phenothiazine (3bf) were not detected.

**Scheme 4:**
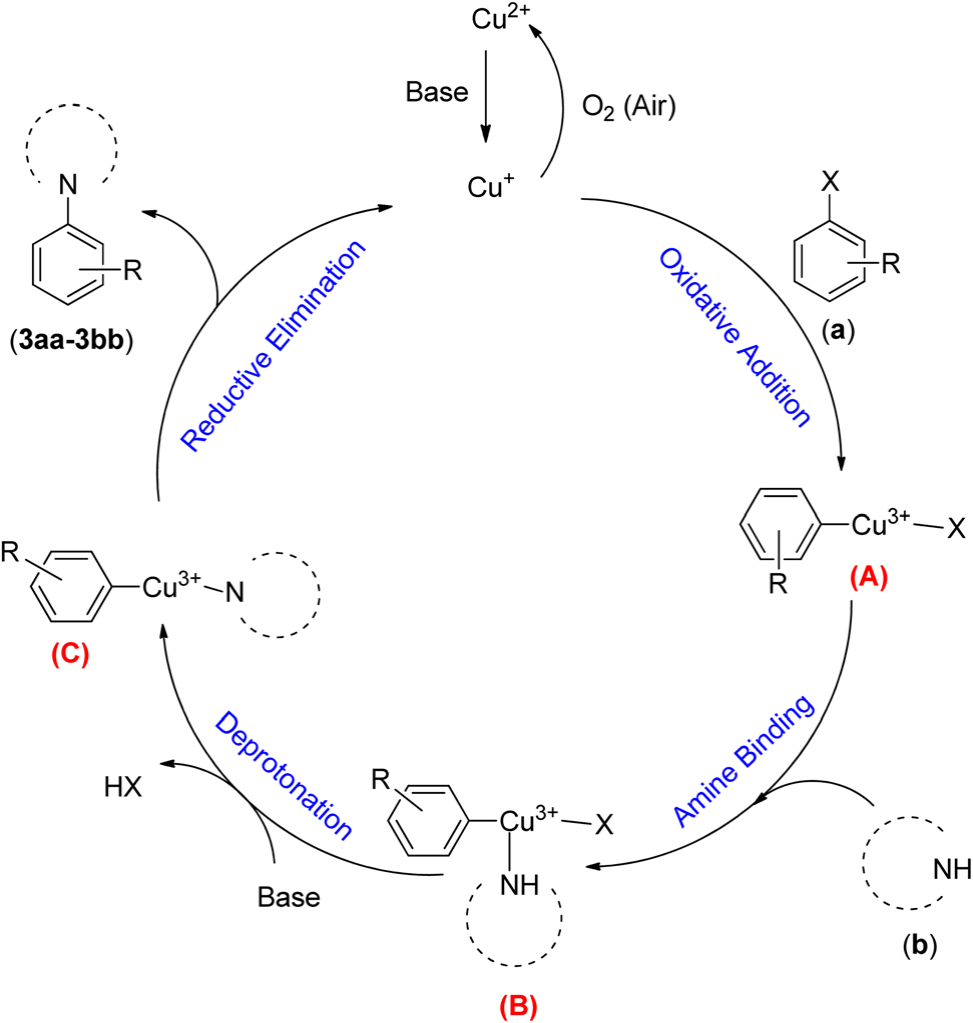
The possible mechanistic pathway of the Buchwald–Hartwig C–N bond formation reaction in the presence of Fe_3_O_4_@SiO_2_-Pr-DEA-[NTA-Cu(ii)]_2_ NPs.

A plausible mechanistic pathway is proposed for the Buchwald–Hartwig C–N bond formation reaction of aryl halides (a) and *s*-amines (b) in the presence of NTAC-Cu(ii)-SSCNM NPs, which serve as a new, highly efficient and magnetically separable nanocatalyst (Scheme 4).^29,30^ As shown in Scheme 4, the catalytic cycle starts with the *in situ* generation of Cu(i) from Fe_3_O_4_@SiO_2_-Pr-DEA-[NTA-Cu(ii)]_2_ NPs under the reaction conditions. After this, the transient Cu(iii) species (A) is produced through oxidative addition, followed by the addition of the NH compound, which leads to the formation of intermediate (B). Then, the reductive elimination process from (C) produces the C–N bond product. Finally, the Cu(i) species is reoxidized to Cu(ii) in the presence of air,^31^ and the catalytic cycle continues in the same way until the end of the reaction.

**Fig. 11:**
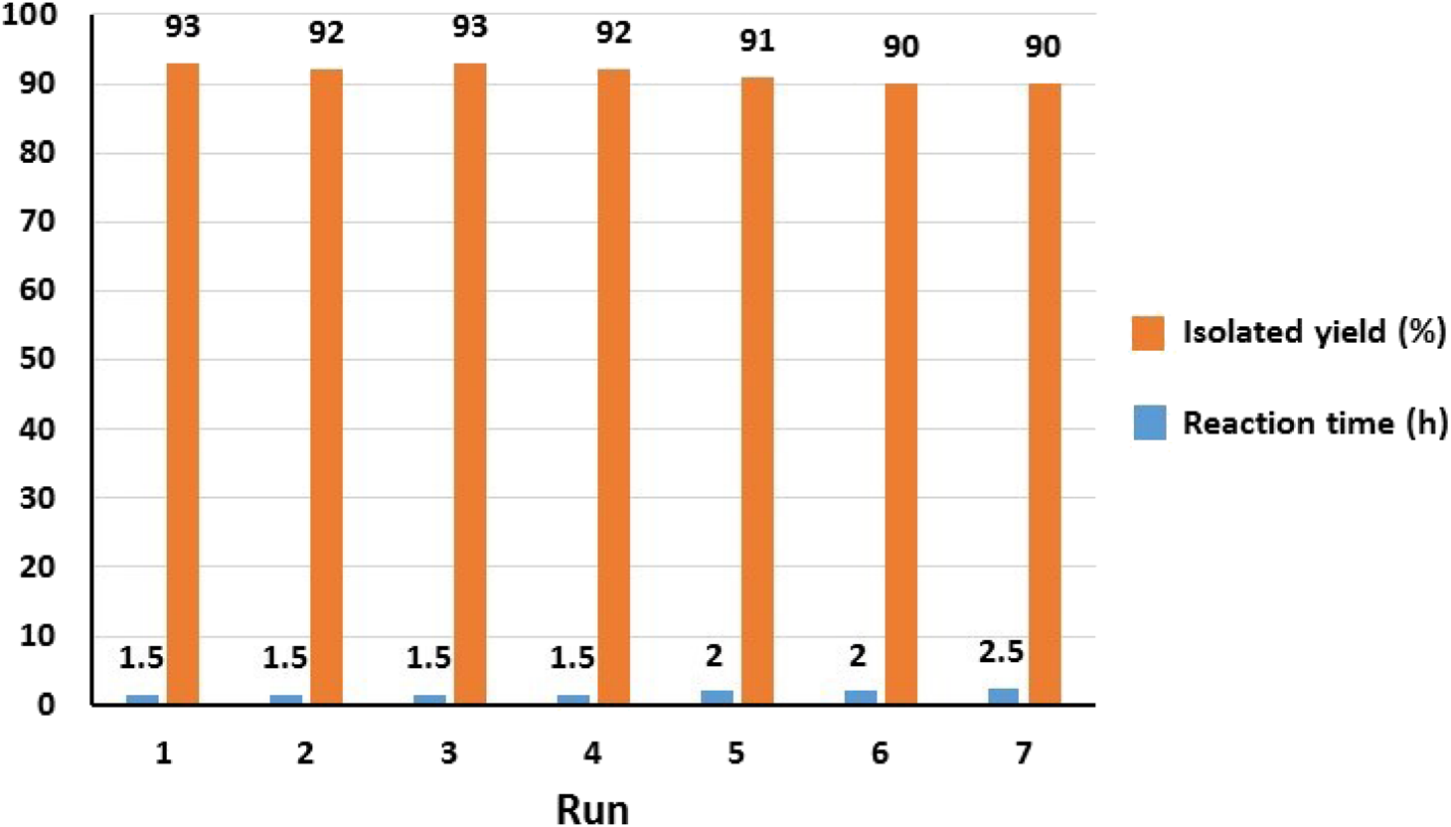
The reusability analysis of Fe_3_O_4_@SiO_2_-Pr-DEA-[NTA-Cu(ii)]_2_ NPs in the C–N bond formation reaction of imidazole and iodobenzene.

Considering the principles of green chemistry, facile separation and reusability are among the essential requirements of an efficient and environmentally friendly catalyst. The reusability of Fe_3_O_4_@SiO_2_-Pr-DEA-[NTA-Cu(ii)]_2_ NPs as a highly efficient and nanomagnetic catalyst was studied in the model reaction of imidazole (2a) and iodobenzene (1a) under the optimized reaction conditions. After the completion of the reaction, the catalyst was simply separated using an external magnet, washed with ethanol, and reused in the next run after drying at 70 °C for 12 h. The model reaction could be run seven times with the recovered Fe_3_O_4_@SiO_2_-Pr-DEA-[NTA-Cu(ii)]_2_ NTAC-Cu(ii)-SSCNM NPs in each run without any considerable loss of catalytic activity (Fig. 11).

**Fig. 12:**
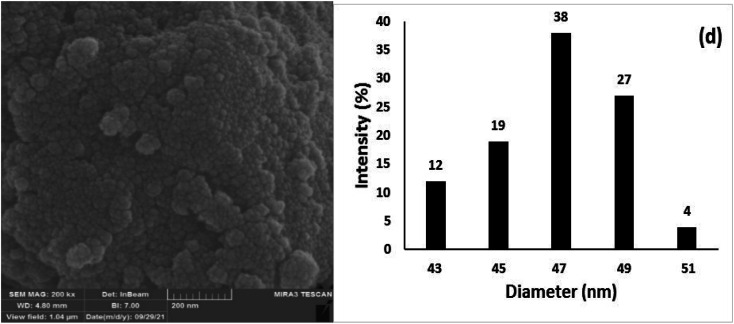
The (a) IR, (b) XRD, (c) FESEM, and (d) DLS of Fe_3_O_4_@SiO_2_-Pr-DEA-[NTA-Cu(ii)]_2_ recovered after the 7th reuse cycle.

The chemical and physical stabilities of the catalyst were investigated after the 7th reuse cycle by IR, XRD, FESEM, DLS, and ICP-OES analyses (Fig. 12). The IR of the recovered catalyst after the 7th reuse cycle (Fig. 12a) was completely the same as the freshly synthesized catalyst (Fig. 1f), indicating the stability of the chemical structure of the catalyst. Moreover, there was no difference between the XRD of the freshly synthesized (Fig. 2c) and recovered catalysts after the 7th catalytic cycle (Fig. 12b), and the mean size of the Fe_3_O_4_ nanocores was calculated using the Debye–Scherrer equation to be around 15 nm, which is approximately the same as that of the freshly synthesized catalyst.

The FESEM image and DLS results of the recovered catalyst after the 7th reuse cycle are presented in Fig. 12c and d, respectively. In the SEM image, significant changes were not observed in the surface morphology of the catalyst, and the recovered nanoparticles showed approximately spherical shapes (Fig. 12c). The DLS analysis results indicate that the size distribution was mostly between 47–49 nm, which is larger than the size of the freshly prepared nanocatalyst and may be the factor responsible for the smooth decrease in the catalytic activity of the recovered catalyst from the 5th reuse cycle. The Cu content of the recovered catalyst after the 7th reuse cycle was investigated by ICP-OES analysis and was determined to be 1.275 mmol g^−1^, indicating the stability of Cu in the structure of the catalyst and the lack of leaching.

**Fig. 13:**
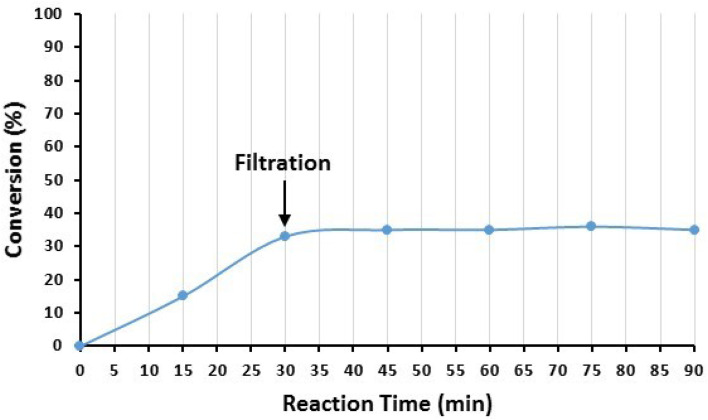
The hot filtration test of Fe_3_O_4_@SiO_2_-Pr-DEA-[NTA-Cu(ii)]_2_ NPs in the catalyzed Buchwald–Hartwig C–N bond formation reaction between iodobenzene (1a) and imidazole (2a).

Moreover, the possibility of Cu leaching from the surface of the nanocatalyst was also investigated by the hot filtration test after the model reaction of iodobenzene (1a) and imidazole (2a). After 30 min of the reaction, the catalyst NPs were removed from the reaction mixture, and the progress of the reaction was checked in the residue. The results are demonstrated in Fig. 13. As seen in Fig. 13, the reaction stopped after the removal of the catalyst NPs, indicating that no catalytically active copper species were present in the residue solution. In addition, the existence of Cu species in the reaction solution was checked after the completion of the reaction by ICP-OES. For this purpose, the model reaction was conducted under optimized conditions, and after the completion of the reaction, the catalyst was separated, and the solvent was evaporated under reduced pressure. The residue was dissolved in HNO_3_ and subjected to ICP-OES analysis. The results showed that copper did not exist in the analysed samples. Both findings indicate that copper species did not leach from the surface of Fe_3_O_4_@SiO_2_-Pr-DEA-[NTA-Cu(ii)]_2_ NPs into the solution, and the reactions were heterogeneously catalyzed.

Finally, to evaluate the efficiency of Fe_3_O_4_@SiO_2_-Pr-DEA-[NTA-Cu(ii)]_2_ NPs as a new, highly efficient, magnetically separable and reusable nanocatalyst, its activity in the Buchwald–Hartwig C–N bond formation reaction of iodobenzene (1a) and imidazole (2a) was compared with some other catalysts that have been reported previously. The data listed in Table 4 show that Fe_3_O_4_@SiO_2_-Pr-DEA-[NTA-Cu(ii)]_2_ performs the reaction in a shorter duration using a lower amount of copper and produces the desired product (3a) with excellent yield. Another important advantage of the as-prepared nanomagnetic catalyst is its facile separability from the reaction media and reusability.

**Table 4:** The comparison of the catalytic activity of Fe_3_O_4_@SiO_2_-Pr-DEA-[NTA-Cu(ii)]_2_ NPs with some catalysts reported for the Buchwald–Hartwig C–N bond formation reaction between iodobenzene and imidazole

Entry	Catalyst (mol%)	Reaction conditions	Time (h)	Yield^a^ (%)	TOF (h^−1^)	References
1	CuFAP (12.5 mol%)	DMSO, K_2_CO_3_, 110 °C	6	92	1.22	32
2	Cu–Y zeolite (10.8 mol%)	DMF, K_2_CO_3_, 120 °C	24	99	0.38	33
3	CuI/MNP-3 (10 mol%)	DMF, Cs_2_CO_3_, 110 °C	24	98	0.40	34
4	Silica immobilized copper catalyst (5 mol%)	Toluene, Cs_2_CO_3_, 100 °C	8	92	2.3	35
5	Cu(ii)-pyridine-based polydentate	DMSO, NaO^*t*^Bu, 120 °C	1.5	97	6.4	36
6	Cu(ii) complex of *para*-hydroxy substituted salen	NaOH, 120 °C	7.0	87	1.77	37
7	Fe_3_O_4_@SiO_2_-dendrimer-encapsulated Cu(ii) NPs (0.5mol%)	DMF, Cs_2_CO_3_, 110 °C	4	96	48	23
8	Fe_3_O_4_@SiO_2_-Pr-DEA-[NTA-Cu(ii)]_2_ (6.4 mol%)	DMF, Cs_2_CO_3_, 110 °C	1.5	93	9.68	This work

a Isolated yield.

## Conclusions

In summary, the NTA complex of Cu(ii) supported on silica-coated nanosized magnetite Fe_3_O_4_@SiO_2_-Pr-DEA-[NTA-Cu(ii)]2 was designed as a new magnetically separable and reusable nanocatalyst and successfully synthesized. Moreover, it was fully characterized by IR, XRD, FESEM, TEM, EDX, FESEM-EDX mapping, VSM, DLS, TGA, BET, solid-state UV-Vis spectroscopy and ICP-OES techniques. Afterward, the synthesized magnetic nanocatalyst was applied in the Buchwald–Hartwig C–N bond formation reaction of aryl halides and nitrogen heterocycles, such as imidazole, 2-methyl-1*H*-imidazole, benzimidazole, indole, and 10*H*-phenothiazine, as well as some aliphatic s-amines, such as piperidine, piperazine, morpholine, dimethylamine, and diethylamine. In all cases, the desired products were obtained with good to excellent yields in DMF at 110 °C. The efficiency of the developed method is highly sensitive to the C–X (X: halide) bond reactivity; a decrease in reaction yield and an increase in reaction time were observed with the change of aryl iodide to aryl bromide and aryl chloride. The catalyst could be separated easily with an external magnet and reused 7 times without any loss in catalytic activity. Further studies prove the chemical and physical stability of the applied catalyst after the 7th reuse cycle. Moreover, the results of the hot filtration studies show the lack of copper leaching from the catalyst surface and prove the ability of the heterogeneous copper catalyst, Fe_3_O_4_@SiO_2_-Pr-DEA-[NTA-Cu(ii)]_2_, in catalysing the reaction under optimized reaction conditions. High yields of products, short reaction times, ease of catalyst separation, reusability of the catalyst, stability of the chemical and physical structures of the catalyst, versatility, and generality of the method, and the simple workup procedure are the main advantages of the as-developed method for the Buchwald–Hartwig C–N bond formation reaction.

## Data availability

The data supporting this article have been included in ESI.†

## Author contributions

Kimia Rajabzadeh: methodology, writing – original Draft, validation, formal analysis, investigation, resources, data curation, visualization. Ali Reza Sardarian: conceptualization, project administration, supervision, resources, funding acquisition, writing – review & editing.

## Conflicts of interest

The authors declare no conflict of interest.

## Supplementary Material

RA-014-D4RA03675A-s001
