# Supplementary material for: Well-defined nanomagnetic nitrilotriacetic acid complex of Cu(ii) supported on silica-coated nanosized magnetite: a new highly efficient and magnetically separable catalyst for C–N bond formation
Source: RSC Adv. 2024 Jul 11;14(30):21954–70. doi: 10.1039/d4ra03675a (PMC11237964; doi:10.1039/d4ra03675a)
Supplement: RA-014-D4RA03675A-s001 [file RA-014-D4RA03675A-s001.pdf]

Electronic Supplementary Material (ESI) for RSC Advances.
This journal is © The Royal Society of Chemistry 2024

Supporting Information

Well-defined Nanomagnetic Nitrilotriacetic Acid Complex of Cu(II) Supported on the Silica-Coated Nanosized Magnetite: as A New Highly Efficient and Magnetically Separable Catalyst for C-N Bond Formation

Kimiya Rajabzadeh[a], Alir Reza Sardarian *[a]

[a] Chemistry Department, College of Sciences, Shiraz University, Shiraz, 71946-84795, Iran

Table of Contents

Characterization data of products (3aa-3bb) .... page S2

References .... page S6

^{1}H, ^{13}C, and IR spectra of products .... page S9

Characterization data of products (3aa-3bb)

1-Phenyl-1*H*-imidazole (3aa): Dark brown oil [1]; IR (KBr) ῡ (cm^{-1}): 3406 (NH), 1494 (C=C); ^1H NMR (250 MHz, DMSO-d_6): δ (ppm)= 8.26 (s, 1H), 7.74 (s, 1H), 7.66-7.62 (m, 2H), 7.54-7.47 (m, 2H), 7.37-7.31 (m, 1H), 7.10 (s, 1H); ^{13}C NMR (63 MHz, $CDCl_3$): δ= 118.2, 121.4, 127.5, 129.9, 130.4, 135.5, 137.4.

1-(4-Nitrophenyl)-1*H*-imidazole (3ab): Yellow solid; M.P.= 207-209 °C (207-209 °C [2]); IR (KBr) ῡ (cm^{-1}): 3443, 2923, 1628, 1518, 1462, 1301, 829; ^1H NMR (250 MHz, $CDCl_3$): δ (ppm) = 8.46-8.21 (m, 2H), 7.93 (s, 1H), 7.55-7.49 (m, 2H), 7.22-7.20 (m, 2H); ^{13}C NMR (63 MHz, $CDCl_3$): δ= 117.7, 121.7, 125.2, 130.9, 136.3, 142.9, 147.0; Anal. Calcd for $C_9H_7N_3O_2$: C, 57.14; H, 3.73; N, 22.21%. Found: C, 57.1; H, 3.69; N, 22.30%.

1-(4-Chlorophenyl)-1*H*-imidazole (3ac): Light brown solid; M.P.= 83-85 °C (85-87 °C [1]); IR (KBr) ῡ (cm^{-1}): 3409, 3112, 1713, 1604, 1504, 1303, 1054, 961, 754, 684; ^1H NMR (250 MHz, $CDCl_3$): δ (ppm) = 8.48-8.26 (m, 2H), 8.10-7.91 (m, 1H), 7.62-7.54 (m, 2H), 7.39-7.36 (m, 1H), 7.27-7.25 (m, 1H); Anal. Calcd for $C_9H_7ClN_2$: C, 60.52; H, 3.95; N, 15.68%. Found: C, 60.48; H, 4.2; N, 15.49%.

1-(Naphthalen-1-yl)-1*H*-imidazole (3ad): Brown oil [3]; IR (KBr) ῡ (cm^{-1}): 3056, 1631, 1577, 1442, 1136, 856, 801; ^1H NMR (300 MHz, $CDCl_3$): δ (ppm) = 7.95 (d, J = 6.0 Hz, 2H), 7.81 (s, 1H), 7.60-7.51 (m, 4H), 7.44 (d, J = 9.0 Hz, 1H), 7.32-7.18 (m, 2H); ^{13}C NMR (75 MHz, $CDCl_3$): δ (ppm) = 134.1, 133.9, 129.4, 129.2, 128.3, 127.7, 127.0, 125.2, 123.7, 122.2, 121.8.

2-(1*H*-Imidazol-1-yl) pyridine (3ae): Dark brown oil [1]; IR (KBr) ῡ (cm^{-1}): 33369, 3059, 1682, 1594, 1478, 1156, 710, 654; ^1H NMR (400 MHz, $CDCl_3$): δ (ppm) = 8.55-8.27 (m, 2H), 7.81-7.76 (m, 1H), 7.66 (s, 1H), 7.34 (d, J = 8.0 Hz, 1H), 7.30-7.18 (m, 2H); ^{13}C NMR (101 MHz, $CDCl_3$): δ (ppm)= 149.1, 139.0, 135.0, 130.5, 122.0, 116.4, 112.3.

1,4-Di(1*H*-imidazol-1-yl)benzene (3af): Yellow solid; M.P.= 208-209 °C (208-210 °C [4]); IR (KBr) ῡ (cm^{-1}): 3409, 1496; ^1H NMR (250 MHz, $CDCl_3$): δ (ppm) = 7.75 (s, 2H), 7.54-7.49 (m, 2H), 7.24-7.13 (m, 6H); Anal. Calcd for $C_{12}H_{10}N_4$: C, 68.56; H, 4.79; N, 26.65%. Found: C, 68.52; H, 4.52; N, 26.42%.

2-Methyl-1-phenyl-1*H*-imidazole (3ag): Yellow oil [5]; ^{1}H NMR (300 MHz, $CDCl_3$): δ (ppm) = 7.63-7.54(m, 2H), 7.46-7.37(m, 3H), 7.27-7.24 (m, 2H), 2.01 (s, 3H); ^{13}C NMR (63 MHz, $CDCl_3$): δ= 13.7, 120.6, 125.5, 127.5, 128.2, 129.5, 137.8, 144.6.

2-Methyl-1-(4-nitrophenyl)-1*H*-imidazole(3ah): Yellow solid; M.P.= 128-130 °C (128-130 °C [6]); IR (KBr) ῡ (cm^{-1}): 2916, 2848, 1695, 1501, 1417, 1339, 1175, 739, 696; ^{1}H NMR (300 MHz, $CDCl_3$): δ (ppm) = 8.37 (d, J = 9.0 Hz, 2H), 7.51 (d, J = 9.0 Hz, 2H), 7.09 (s, 2H), 2.45 (s, 3H); ^{13}C NMR (75 MHz, $CDCl_3$): δ (ppm) = 146.9, 144.5, 143.0, 128.5, 125.8, 125.2, 120.3, 14.1; Anal. Calcd for $C_{10}H_9N_3O_2$: C, 59.11; H, 4.46; N, 20.68%. Found: C, 589; H, 4.5; N, 20.72%.

2-(2-Methyl-1*H*-imidazol-1-yl)pyridine (3ai): Yellow oil; IR (KBr) ῡ (cm^{-1}): 3365, 3113, 2930, 1671, 1529, 1473, 1377, 1185, 740, 679; ^{1}H NMR (300 MHz, $CDCl_3$): δ (ppm) = 8.59-8.57 (m, 1H), 7.91-7.85 (m, 1H), 7.37-7.31 (m, 3H), 7.07 (d, J= 7.5 Hz,1H), 2.64 (s, 3H); ^{13}C NMR (75 MHz, $CDCl_3$): δ (ppm) = 150.3, 149.3, 144.9, 138.8, 127.0, 122.7, 119.1, 117.3, 15.1.

1-(4-Nitrophenyl)-1*H*-benzo[*d*]imidazole (3aj): Yellow solid, M.P. > 300 °C (> 300 °C [7]); IR (KBr) ῡ (cm^{-1}): 1503, 1352, 867, 858, 731; ^{1}H NMR (250 MHz, DMSO-d_6): δ (ppm) = 8.72 (s, 1H), 8.46-8.40 (m, 2H), 8.04-7.98 (m, 2H), 7.81-7.74 (m, 2H), 7.41-7.30 (m, 2H); Anal. Calcd for $C_{13}H_9N_3O_2$: C, 65.27; H, 3.79; N, 17.56%. Found: C, 65.33; H, 3.62; N, 17.08%.

1-(*p*-Tolyl)-1*H*-benzo[*d*]imidazole (3ak): Yellow solid, M.P.> 300 °C, IR (KBr) ῡ (cm^{-1}): 3390, 2919, 2858, 1609, 1516, 1453, 1375, 1155, 819; ^{1}H NMR (300 MHz, $CDCl_3$): δ (ppm) = 8.16 (s, 1H), 7.91 (s, 1H), 7.53-7.29 (m, 7H), 2.47 (s, 3H); ^{13}C NMR (75 MHz, $CDCl_3$): δ (ppm) = 143.7, 142.4, 138.2, 133.7, 130.6, 124.0, 123.7, 122.8, 120.4, 110.6, 21.2; Anal. Calcd for $C_{14}H_{12}N_2$: C, 80.74; H, 5.81; N, 13.45%. Found: C, 80.71; H, 5.82; N, 13.47%.

1-(4-Methoxyphenyl)-1*H*-benzo[*d*]imidazole (3al): Yellow solid; M.P.= 96-98 °C (96-97 °C [8]); IR (KBr) ῡ (cm^{-1}): 3448, 2927, 2800, 1613, 1515, 1458, 1334, 1247, 1177, 833; ^{1}H NMR (250 MHz, $CDCl_3$): δ (ppm) = 8.06 (s, 1H), 7.89-7.86 (m, 1H), 7.54-7.42 (m, 2H), 7.42-7.36 (m, 1H), 7.34-7.26 (m, 2H), 7.10-7.06 (m, 2H), 3.89 (s, 3H), ^{13}C NMR (101 MHz, $CDCl_3$): δ (ppm) = 158.3, 136.3, 132.9, 129.0, 128.3,

126.0, 122.2, 121.1, 120.1, 114.8, 110.4, 102.9, 55.6; Anal. Calcd for $C_{14}H_{12}N_2O$: C, 74.98; H, 5.39; N, 12.49%. Found: C, 74.89; H, 5.25; N, 12.68%.

1-Phenyl-1*H*-indole (3am): Colorless oil [9]; IR (KBr) ῡ (cm^{-1}): 694 , 748 , 1018 , 1027 , 1134 , 1227 , 1327 , 1458 , 1504 , 1596 , 2923 , 3055 ; ^1H NMR (250 MHz, $CDCl_3$): δ (ppm) = 7.77-7.68 (m, 1H), 7.62-7.58 (m, 1H), 7.56-7.50 (m, 4H), 7.43-7.34 (m, 2H), 7.26-7.22 (m, 2H), 6.74-6.69 (m, 1H); ^{13}C NMR (63 MHz, $CDCl_3$): δ= 103.6, 110.5, 120.4, 121.1, 122.4, 124.4, 126.4, 128.0, 129.3, 129.6, 135.8, 139.8.

1-(4-Nitrophenyl)-1*H*-indole (3an): Yellow solid; M.P.= 131-133 °C (131-132 °C [10]); IR (KBr) ῡ (cm^{-1}): 1641, 1594, 1566, 1505, 1456, 1326, 1137, 1069, 953, 854; ^1H NMR (250 MHz, $CDCl_3$): δ (ppm)= 7.61-7.58 (m, 1H), 7.44-7.31 (m, 5H), 7.20-7.06 (m, 3H), 6.61-6.59 (m, 1H); Anal. Calcd for $C_{14}H_{10}N_2O_2$: C, 70.58; H, 4.23; N, 11.76%. Found: C, 69.84; H, 4.22; N, 11.53%.

1-(*p*-Tolyl)-1*H*-indole (3ao): Yellow solid; M.P.= 38-40 °C (38-39 °C [11]); IR (KBr) ῡ (cm^{-1}): 2921, 1608, 1519, 1457, 1333, 1213, 821, 740 ; ^1H NMR (250 MHz, $CDCl_3$): δ (ppm) = 7.63-7.56 (m, 1H), 7.49-7.41 (m, 1H), 7.32-7.27 (m, 2H), 7.23-7.19 (m, 3H), 7.16-7.03 (m, 2H), 6.60-6.55 (m, 1H), 2.34 (s, 3H); ^{13}C NMR (63 MHz, $CDCl_3$): δ= 21.1, 103.2, 110.5, 120.1, 121.1, 122.2, 124.3, 128.1, 129.2, 130.1, 136.3, 137.3; Anal. Calcd for $C_{15}H_{13}N$: C, 86.92; H, 6.32; N, 6.76%. Found: C, 86.56; H, 6.54; N, 6.9%.

1-(4-Methoxyphenyl)-1*H*-indole (3ap): Yellow solid; M.P.= 56-59 °C (56-58 °C [12]); IR (KBr) ῡ (cm^{-1}): 3445, 2929, 2839, 1612, 1516, 1457, 1287, 1251, 1179, 835; ^1H NMR (250 MHz, $CDCl_3$): δ (ppm) = 8.06 (s, 1H), 7.95-7.81 (m, 1H), 7.54-7.38 (m, 3H), 7.36-7.26 (m, 2H), 7.15-7.03 (m, 2H), 3.90 (s, 3H); ^{13}C NMR (101 MHz, $CDCl_3$): δ (ppm) = 159.4, 143.8, 142.6, 134.2, 129.1, 125.7, 123.6, 122.6, 120.5, 115.1, 110.4, 55.7; Anal. Calcd for $C_{15}H_{13}NO$: C, 80.69; H, 5.87; N, 6.27%. Found: C, 80.35; H, 5.89; N, 6.3%.

10-Phenyl-10*H*-phenothiazine (3aq); Dark brown solid; M.P.= 90-92 °C (90-91°C [13]); IR (KBr) ῡ (cm^{-1}): 434, 529, 625, 707, 744 , 931, 1041, 1128, 1243, 1305, 1457, 1585, 2924, 3057; ^1H NMR (250 MHz, $CDCl_3$): δ (ppm) = 8.30-8.26 (m, 1H), 8.15-8.02 (m, 2H), 7.97-7.91 (m, 1H), 7.76-7.54 (m, 5H), 7.51-7.27 (m, 4H), 6.77-6.72 (m, 1H); Anal. Calcd for $C_{18}H_{13}NS$: C, 78.51; H, 4.76; N, 5.09%. Found: C, 78.62; H, 4.83; N, 4.73%. .

10-(4-Nitrophenyl)-10*H*-phenothiazine (3ar): Yellow solid; M.P= 156-158 °C (156-157 °C [14]); ^{1}H NMR (250 MHz, $CDCl_3$): δ (ppm) = 7.42 (d, J = 7.5 Hz, 1H), 7.31-7.26 (m, 1H), 7.05-6.99 (m, 1H), 6.91-6.74 (m, 2H), 6.22 (d, J = 7.5 Hz, 1H) Anal. Calcd for $C_{18}H_{12}N_2O_2S$: C, 67.48; H, 3.78; N, 8.74%. Found: C, 67.23; H, 3.11; N, 8.61%.

10-(4-Chlorophenyl)-10*H*-phenothiazine(7s): White solid; M.P.= 120-122 °C (120-121°C [14]); IR (KBr) ῡ (cm^{-1}): 3448, 2923, 1625, 1462, 1090, 913; ^{1}HNMR (250 MHz, $CDCl_3$): δ (ppm) = 7.60-7.47(m, 1H), 7.42-7.32 (m, 1H), 7.00-6.88 (m, 1H), 6.82-6.68 (m, 2H), 6.18-6.06 (m, 1H); ^{13}C NMR (101 MHz, $CDCl_3$): δ (ppm) = 143.9, 139.6, 133.8, 132.4, 131.0, 126.9, 126.3, 122.9, 120.9, 116.3; Anal. Calcd for $C_{18}H_{12}ClNS$: C, 69.78; H, 3.90; N, 4.52%. Found: C, 69.74; H, 3.92; N, 4.51%.

1-Phenylpiperidine (3at): Yellow oil[15]; IR (KBr) ῡ (cm^{-1}): 3448, 2923, 1625, 1462, 1090, 913; ^{1}HNMR (250 MHz, $CDCl_3$): δ (ppm) = 7.22-7.17 (m, 2H), 6.68-6.61 (m, 3H), 2.97 (t, J =5.0 Hz, 5H), 1.61-1.52 (m, 4H), 1.49-144 (m, 1H).

1-(4-Nitrophenyl)piperidine (3au): Yellow oil ([16]); IR (KBr) ῡ (cm^{-1}): 2939, 1601, 1514, 1477, 1326, 1114, 821; ^{1}H NMR (250 MHz, $CDCl_3$): δ (ppm) = 8.15-8.05 (m, 2H), 6.82-6.85 (m, 2H), 3.46 (s, 4H), 1.69 (s, 6H) ; ^{13}C NMR (101 MHz, $CDCl_3$): δ (ppm) = 154.9, 137.3, 126.1, 112.3, 48.4, 25.3, 24.2.

1,4-Di(piperidin-1-yl)benzene (3av): Yellow solid; M.P.= 104-106 °C (104-106 °C [16]); IR (KBr) ῡ (cm^{-1}): 3338, 2921, 1598, 1462, 1180, 699, 721; ^{1}H NMR (250 MHz, $CDCl_3$): δ (ppm) = 7.19-7.14 (m, 2H), 6.65-6.59 (m, 2H), 2.95 (t, 5Hz, 8H), 1.64-1.31 (m, 12H); Anal. Calcd for $C_{16}H_{24}N_2$: C, 78.64; H, 9.90; N, 11.46%. Found: C, 78.36; H, 10.4; N, 11.13%.

1,4-Diphenylpiperazine (3aw): Yellow solid; M.P. = 150-152 °C (150-153 °C [17]); IR (KBr) ῡ (cm^{-1}): 2829, 1597, 1429, 1454, 1389, 1324, 1223, 1154, 1031, 984, 924, 765, 691, 527; ^{1}H NMR (300 MHz, $CDCl_3$): δ (ppm) = 7.38-7.33 (m,4H), 7.19 (s,6H), 3.40-3.14 (m,8H); ^{13}C NMR (75 MHz, $CDCl_3$): δ (ppm) = 160.7, 129.7, 123.6, 118.2, 44.6; Anal. Calcd for $C_{16}H_{18}N_2$: C, 80.63; H, 7.61; N, 11.75%. Found: C, 80.59; H, 7.64; N, 11.78%.

4-(4-Nitrophenyl)morpholine (3ax): Yellow solid; M.P.= 147-150 °C (147-149 °C [18]); IR (KBr) ῡ(cm^{-1}): 2935, 1657, 1590, 1452, 1322, 1151, 843; ^{1}H NMR (250 MHz, $CDCl_3$): δ (ppm) = 8.28-8.05 (m, 2H), 6.87-6.80 (m, 2H), 3.91-3.83 (m, 4H), 3.37 (t, *J*= 5.0 Hz, 4H); ^{13}C NMR (101 MHz, $CDCl_3$): δ (ppm) = 154.9, 137.3, 126.1, 112.3, 48.4, 25.3; Anal. Calcd for $C_{10}H_{12}N_2O_3$: C, 57.69; H, 5.81; N, 13.45%. Found: C, 57.61; H, 5.93; N, 13.51%.

1,4-Dimorpholinobenzene (3ay): White solid; M.P.= 191-193 °C (191-192 °C [19]); IR (KBr) ῡ (cm^{-1}): 1583, 1511, 1296, 1114, 857; ^{1}H NMR (250 MHz, $CDCl_3$): δ (ppm) = 7.19-7.14 (m, 2H), 6.65-6.60 (m, 2H), 2.95 (t, *J*= 5.0 Hz, 8H), 1.56-1.42 (m, 8H); Anal. Calcd for $C_{14}H_{20}N_2O_2$: C, 67.72; H, 8.12; N, 11.28%. Found: C, 68.02; H, 7.83; N, 11.41%.

***N,N*-Dimethylaniline (3az):** Colorless liquid [20]; ^{1}H NMR (400 MHz, $CDCl_3$): δ (ppm) = 7.44 (dd, *J*= 8.0 Hz, 2H), 6.93 (t, *J*= 8.0, 3H), 3.11(s, 6H); ^{13}C NMR (101 MHz, $CDCl_3$): δ (ppm) = 150.8, 129.2, 116.7, 112.8, 40.7.

***N,N*-Dimethyl-4-nitroaniline (3ba):** Yellow solid; M.P.= 162-164 °C (162-163 °C [21]); IR (KBr) ῡ (cm^{-1}) : 3086, 2833, 1911, 1603, 1591, 1584, 1536, 1454, 823, 755 ; ^{1}H NMR (400 MHz, DMSO-d_6): δ (ppm) = 8.05 (d, *J* = 9.3 Hz, 1H), 6.76 (d, *J* = 9.5 Hz, 1H), 3.09 (s, 3H); ^{13}C NMR (101 MHz, DMSO-d_6): δ (ppm) = 154.3, 135.5, 125.7, 110.6, 30.6; Anal. Calcd for $C_8H_{10}N_2O_2$: C, 57.82; H, 6.07; N, 16.86%. Found: C, 57.82; H, 6.13; N, 16.54%.

***N,N*-Diethylaniline (3bb):** Yellow oil [1]; ^{1}H NMR (400 MHz, $CDCl_3$): δ (ppm) = 7.41-7.31 (m, 2H), 6.84- 6.77 (m, 3H), 3.48 (q, *J*= 8.0 Hz, 4H), 1.30 (t, *J*= 8.0, 6H) ; ^{13}C NMR (101 MHz, $CDCl_3$): δ (ppm) = 148.0, 129.4, 115.5, 112.0, 44.4, 12.7.

References

1. Sharghi, H.; Sepehri, S.; Aberi, M. Cu(II) Complex of Pyridine-Based Polydentate as a Novel, efficient, and Highly Reusable Catalyst in C–N Bond-Forming Reaction. *Mol. Divers.* **2017**, 21, 855-864.

2. Inaloo, I. D.; Majnooni, S.; Eslahi, H.; Esmaeilpour, M. *N*-Arylation of (Hetero)Arylamines Using Aryl Sulfamates and Carbamates Via C–O Bond Activation Enabled by a Reusable and Durable Nickel(0) Catalyst†. *New. J. Chem.* **2020**, 44, 13266-13278.

3. Altman, R. A.; Buchwald, S. L. 4,7-Dimethoxy-1,10-Phenanthroline: An Excellent Ligand for the Cu-Catalyzed N-Arylation of Imidazoles. *Org. Lett.* **2006**, 8, 2779-2782.

4. Tronnier, A.; Strassner, Th. (C^C*) Cyclometalated Binuclear *N*-Heterocyclic Biscarbene Platinum(Ii) Complexes – Highly Emissive Phosphorescent Emitters†. *Dalton. Trans.* **2013**, 42, 9847-9851.

5. Molaei, H.; Ghanbari, M. M. Practical Copper-Catalyzed *N*-Arylation of Amines with 20% Aqueous Solution of *N*-Bu_4NOH. *Chin. Chem. Lett.* **2012**, 23, 301-304.

6. Zhou, Q.; Du, F.; Chen, Y.; Fu, Y.; Sun, W.; Wu, Y.; Chen, G. l-(−)-Quebrachitol as a Ligand for Selective Copper(0)-Catalyzed N-Arylation of Nitrogen-Containing Heterocycles. *J. Org. Chem.* **2019**, 84, 8160-8167.

7. Wang, Y.; Wu, Z.; Wang, L.; Li, Z.; Zhou, X. A Simple and Efficient Catalytic System for N-Arylation of Imidazoles in Water. *Chem. Eur. J.* **2009**, 15, 18971-18974.

8. Liu, X.; Chang, S.; Chen, X.; Ge, X.; Qian, C. Efficient Ullmann C–X Coupling Reaction Catalyzed by a Recoverable Functionalized-Chitosan Supported Copper Complex. *New J. Chem.* **2018**, 42, 16013-16020.

9. Xiao, R.; Zhao, H.; Cai, M. MCM-41-Immobilized Bidentate Nitrogen Copper(I) Complex: A Highly Efficient and Recyclable Catalyst for Buchwald N-Arylation of Indoles. *Tetrahedron*. **2013**, 69, 5444-5450.

10. Nandi, D.; Siwal, S.; Mallick, K. A Carbon Nitride Supported Copper Nanoparticle Composite: A Heterogeneous Catalyst for the N-Arylation of Hetero-Aromatic Compounds†. New. J. Chem. **2017**, 41, 3082-3088.

11. Maligres, P. E.; Krska, S. W.; Dormer, P. G. A Soluble Copper(I) Source and Stable Salts of Volatile Ligands for Copper-Catalyzed C–X Couplings. *J. Org. Chem.* **2012**, 77, 7646-7651.

12. Ge, X.; Chen, X.; Qian, C.; Zhou, S. Efficient Ullmann C–N Coupling Catalyzed by a Recoverable Oligose-Supported Copper Complex†. *RSC. Adv.* **2016**, 6, 58898-58906.

13. Zhang, L.; Huang, X.; Zhen, S.; Zhao, J.; Li, H.; Yuan, B.; Yang, G. Pd-Catalyzed Double N-Arylation of Primary Amines to Synthesize Phenoxazines And Phenothiazines. *Org. Biomol. Chem.* **2017**, 15, 6306-6309.

14. Clarke, D.; Gilbert, B. C.; Hanson, P. Substituent Effects on the Distribution of the Spin Density in 10-Arylphenoxazine and 10-Arylphenothiazine Cation Radicals. *J. chem. soc Perkin Trans.* **1976**, 2, 114-124.

15. Hie, L.; Ramgren, S. D.; Mesganaw, T.; Garg, N. K.; Nickel-Catalyzed Amination of Aryl Sulfamates and Carbamates Using an Air-Stable Precatalyst. *Org. Lett.* **2012**. 14, 4182-4185.

16. Buch-Rasmussen, Th. Preparation and Properties of *N, N, N', N'*-Tetrasubstituted 1,4-Benzenediamines. *Acta. Chem. Scand.* **1996**, 50, 1013-1019.

17. Sharghi, H.; Shiri, P. 2-Phenyl-2-(4-phenyl-1H-1,2,3-triazol-1-yl)ethanol as an Efficient and Versatile Auxiliary Ligand in Copper(II)-Catalyzed Buchwald–Hartwig and Sharpless–Meldal C–N Bond-Forming Reactions. *Synthesis*. **2015**, 47, 1131-1146.

18. Pilkington, L. I.; Sparrow, K.; Rees, S. W.; Paulin, E. K.; van Rensburg, M.; Xu, C. S.; Barker, D. Development, Synthesis and Biological Investigation of a Novel Class of Potent PC-PLC Inhibitors, *Eur. J. Med. Chem.* **2020**, 191, 112162.

19. Chen, C.; Yang, L. M. Ni(II)−(σ-Aryl) Complex: A Facile, Efficient Catalyst for Nickel-Catalyzed Carbon−Nitrogen Coupling Reactions. *J. Org. Chem.* **2007**, 72, 6324-6327.

20. Dupuy, S.; Steven P. N. Gold(I)-Catalyzed Protodecarboxylation of (Hetero)Aromatic Carboxylic Acids. *Chem. Eur. J.* **2013**, 19, 14034-14038.

21. Dutov, M. D.; Serushkina, O. V. Interaction of Hexamethylphosphoric Triamide with M-Dinitrobenzenes. *Mendeleev. Commun.* **2013**, 23, 174-175.

[1] H, ^{13}C, and IR spectra of products

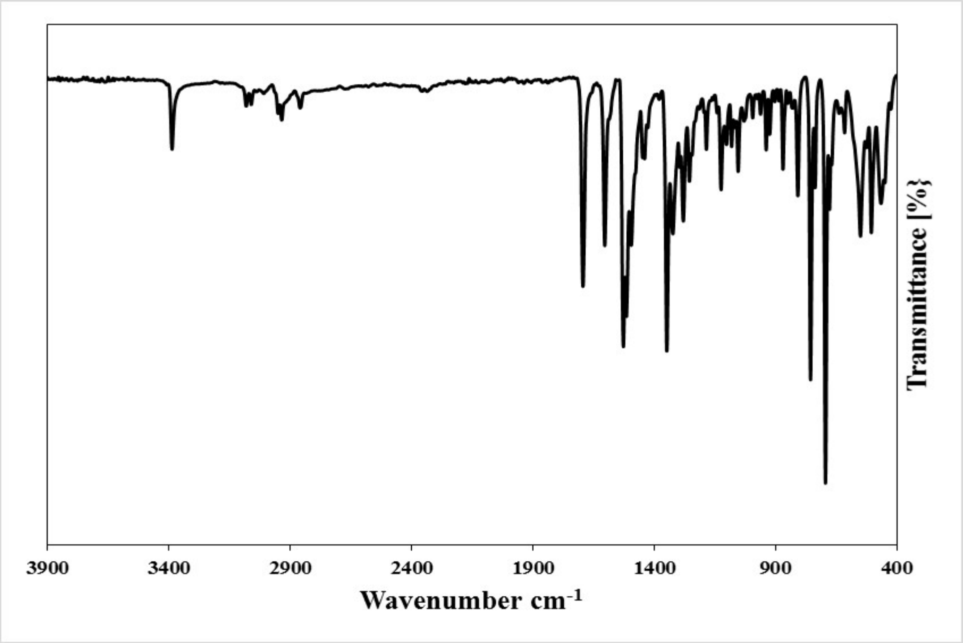

Figure 1. IR spectrum of *anti*-2-((3-nitrophenyl) (phenylamino) methyl) cyclohexan-1-one in KBr.

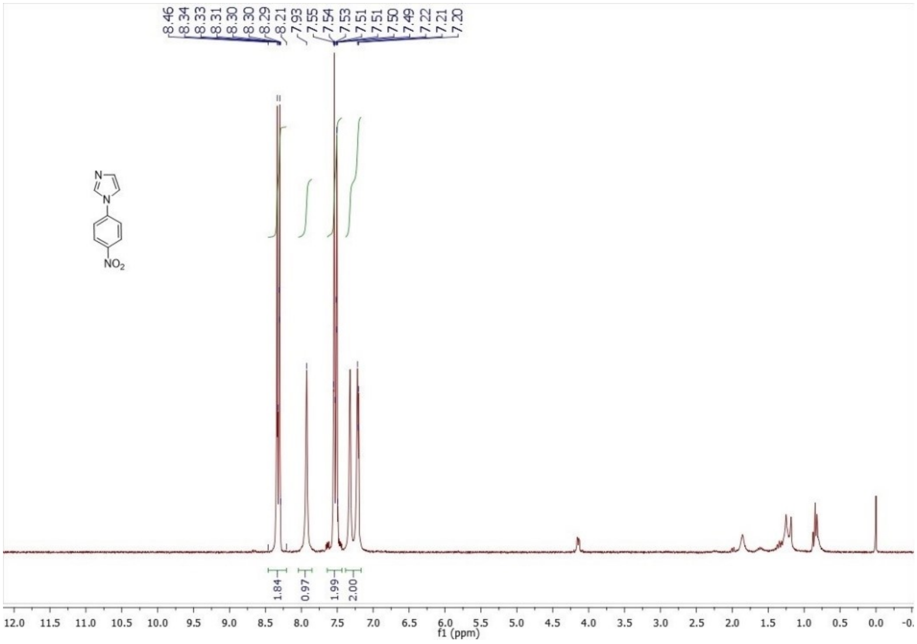

Figure 2. ^{1}H NMR spectrum (250 MHz) of 1-(4-nitrophenyl)-1*H*-imidazole in $CDCl_3$**.**

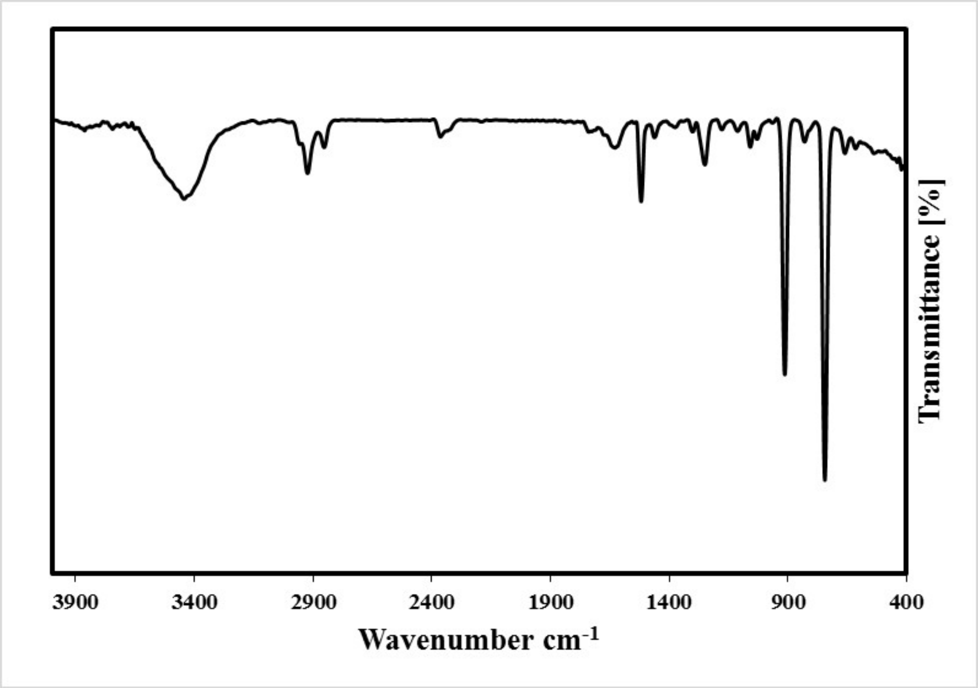

Figure 3. IR spectrum of 1-(4-nitrophenyl)-1*H*-imidazole in KBr.

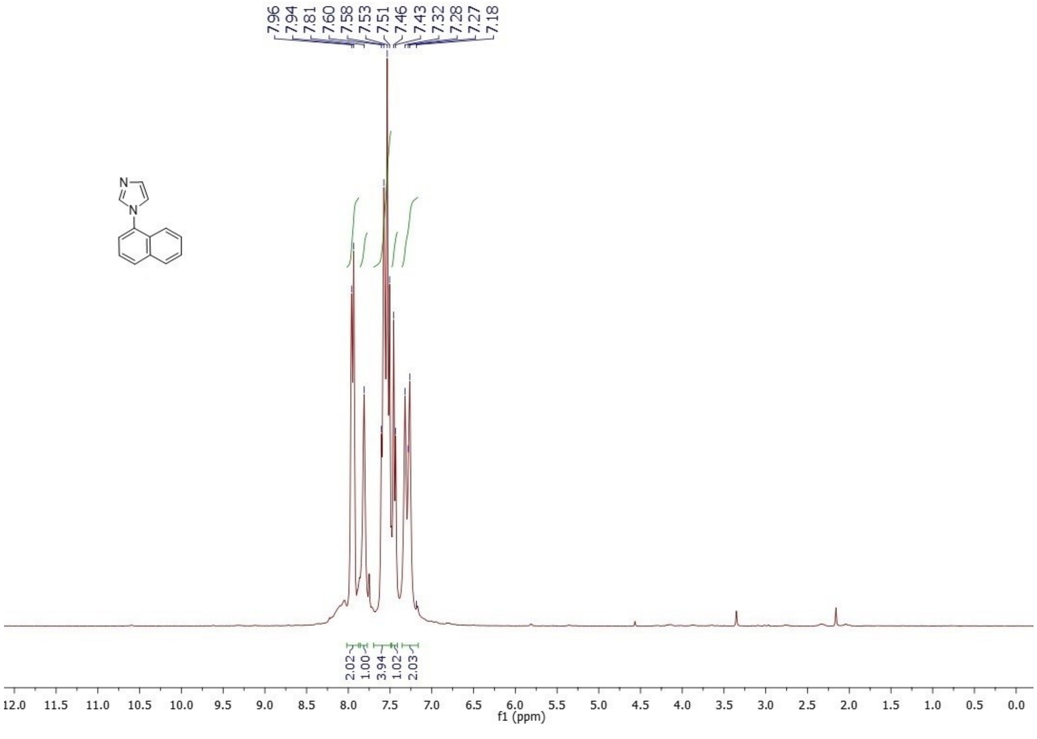

Figure 4. ^{1}H NMR spectrum (300 MHz) of 1-(naphthalen-1-yl)-1*H*-imidazole in $CDCl_3$**.**

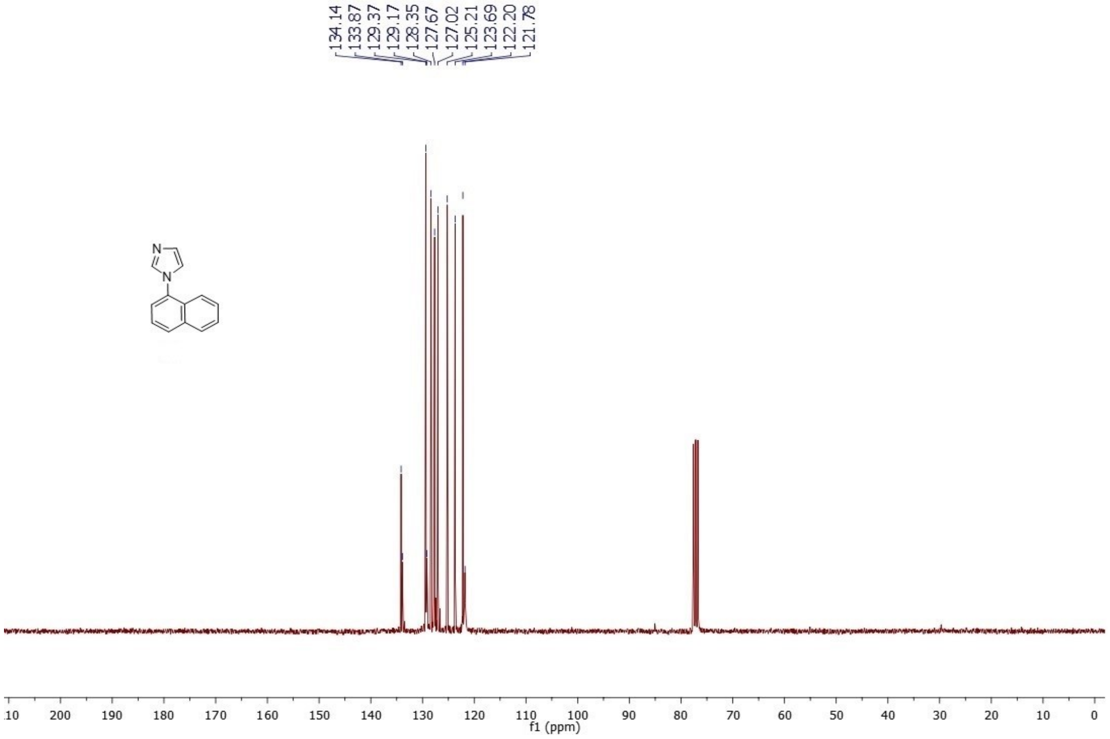

Figure 5. ^{13}C NMR spectrum (75 MHz) of 1-(naphthalen-1-yl)-1*H*-imidazole in DMSO-d_6.

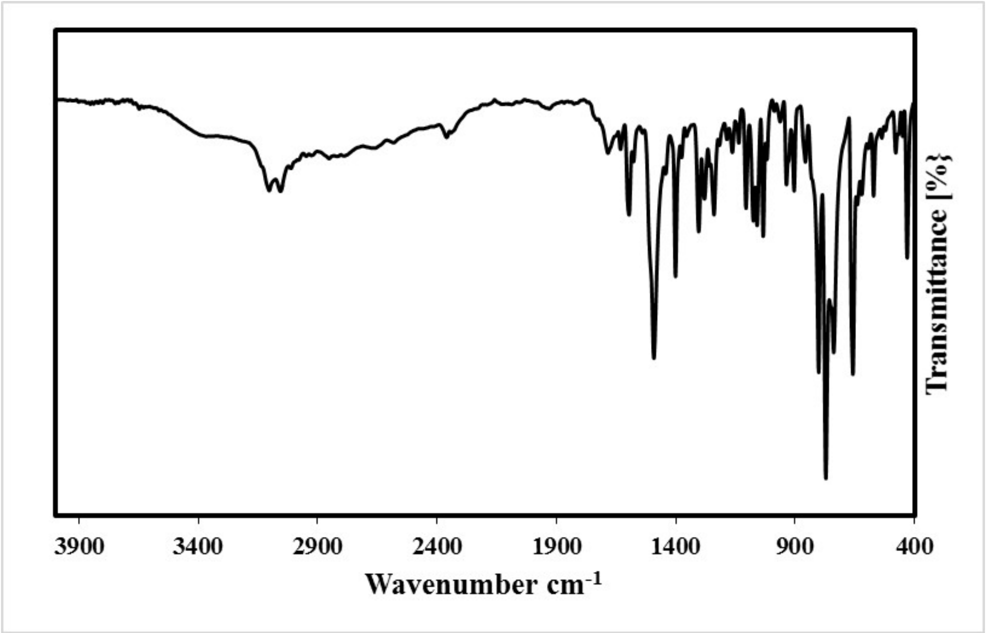

Figure 6. IR spectrum of 1-(naphthalen-1-yl)-1*H*-imidazole in KBr.

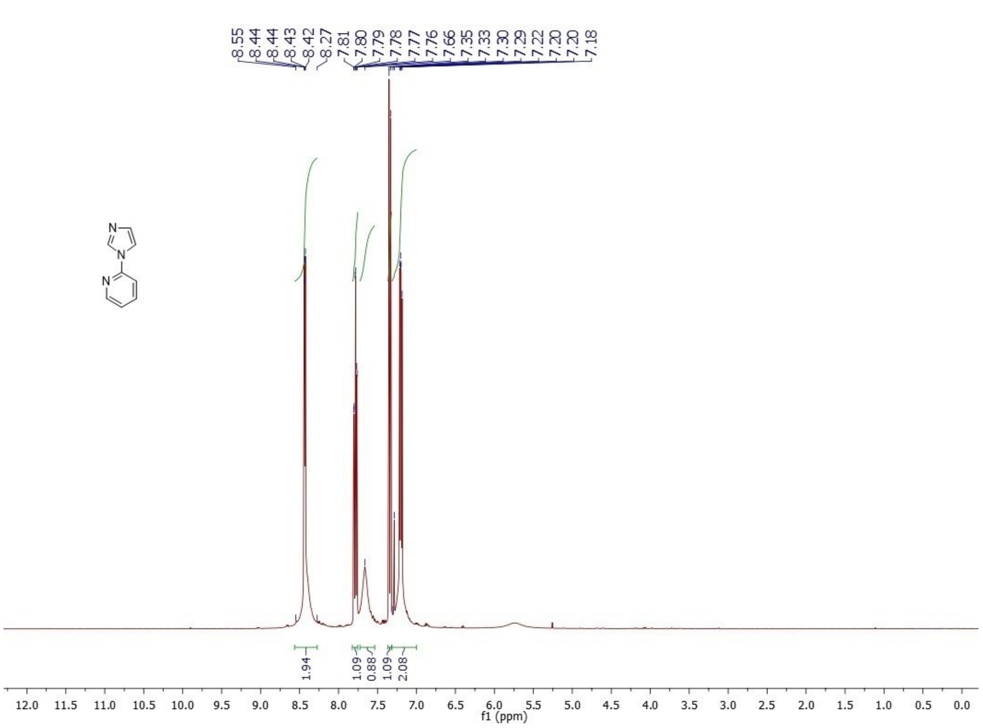

Figure 7. ^{1}H NMR spectrum (400 MHz) of 2-(1*H*-imidazol-1-yl)pyridine in $CDCl_3$**.**

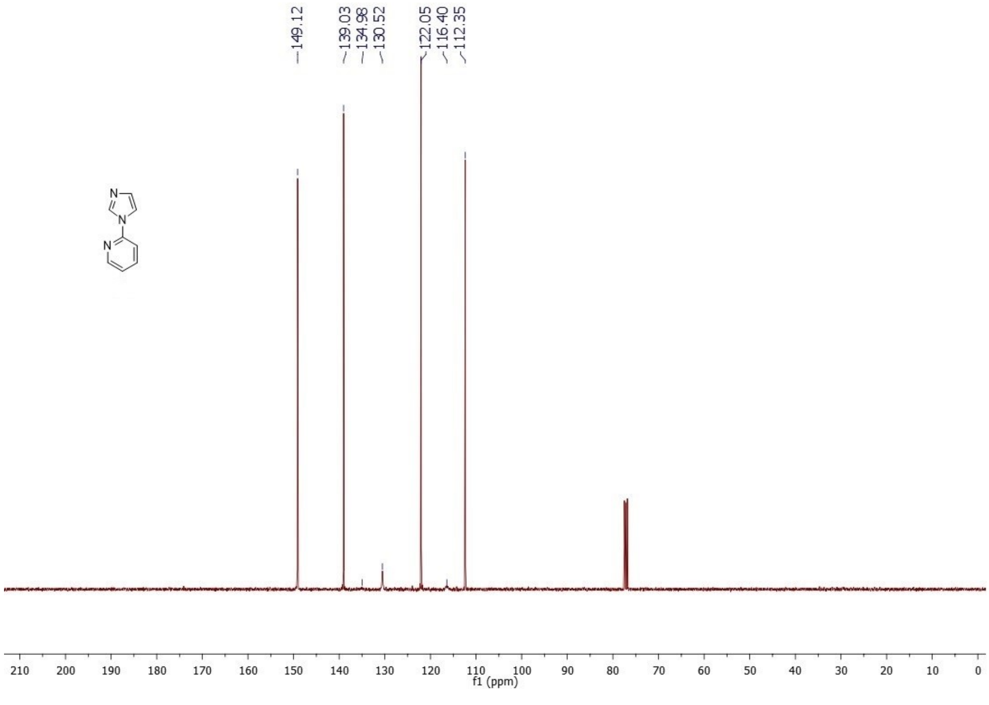

Figure 8. ^{13}C NMR spectrum (101 MHz) of 2-(1*H*-imidazol-1-yl) pyridine in $CDCl_3$.

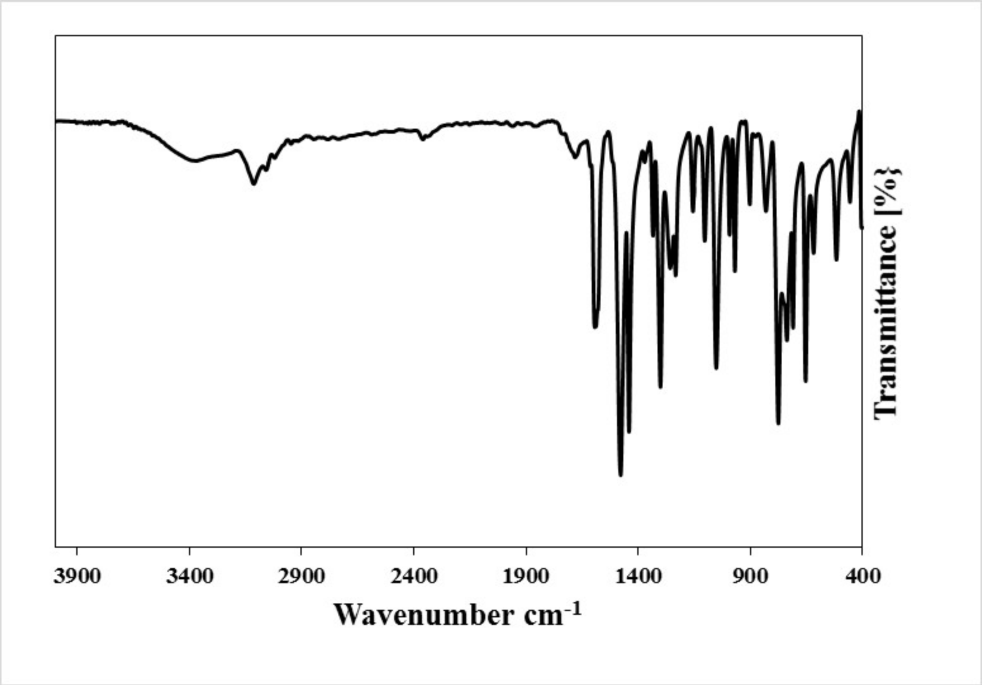

Figure 9. IR spectrum of 2-(1*H*-imidazol-1-yl)pyridine in KBr.

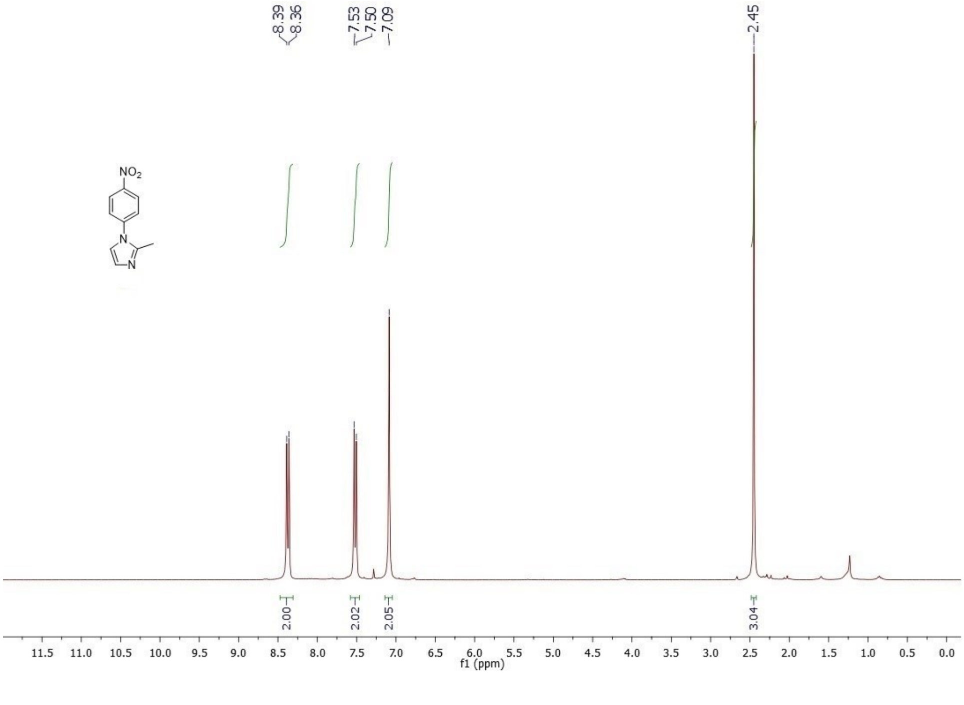

Figure 10. ^{1}H NMR spectrum (300 MHz) of 2-methyl-1-(4-nitrophenyl)-1*H*-imidazole in $CDCl_3$.

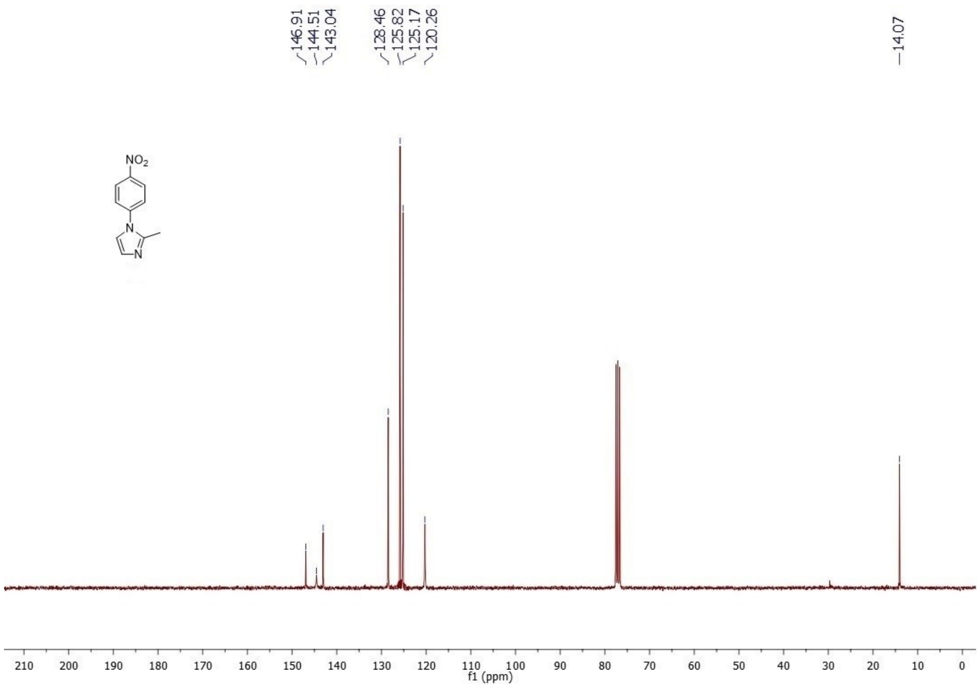

Figure 11. ^{13}C NMR spectrum (75 MHz) of 2-methyl-1-(4-nitrophenyl)-1*H*-imidazole in $CDCl_3$.

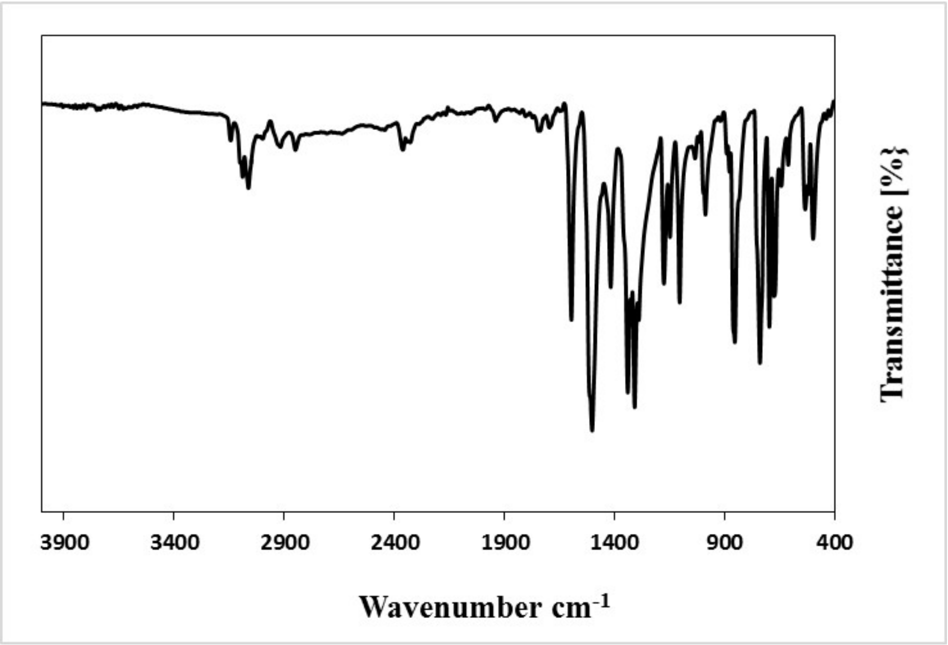

Figure 12. IR spectrum of 2-methyl-1-(4-nitrophenyl)-1*H*-imidazole in KBr.

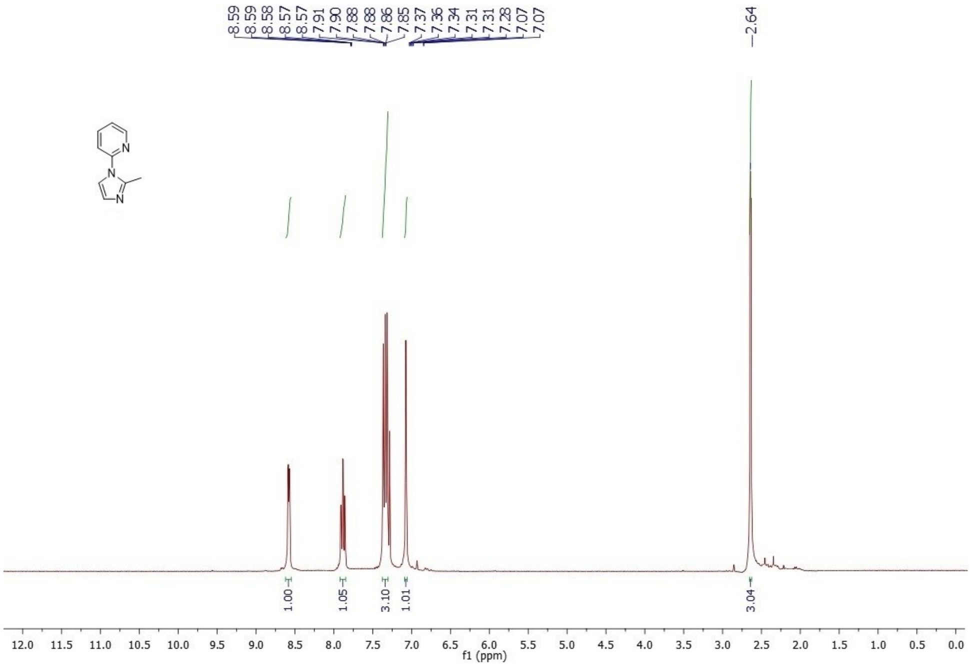

Figure 13. ^{1}H NMR spectrum (75 MHz) of 2-(2-methyl-1*H*-imidazol-1-yl)pyridine in $CDCl_3$.

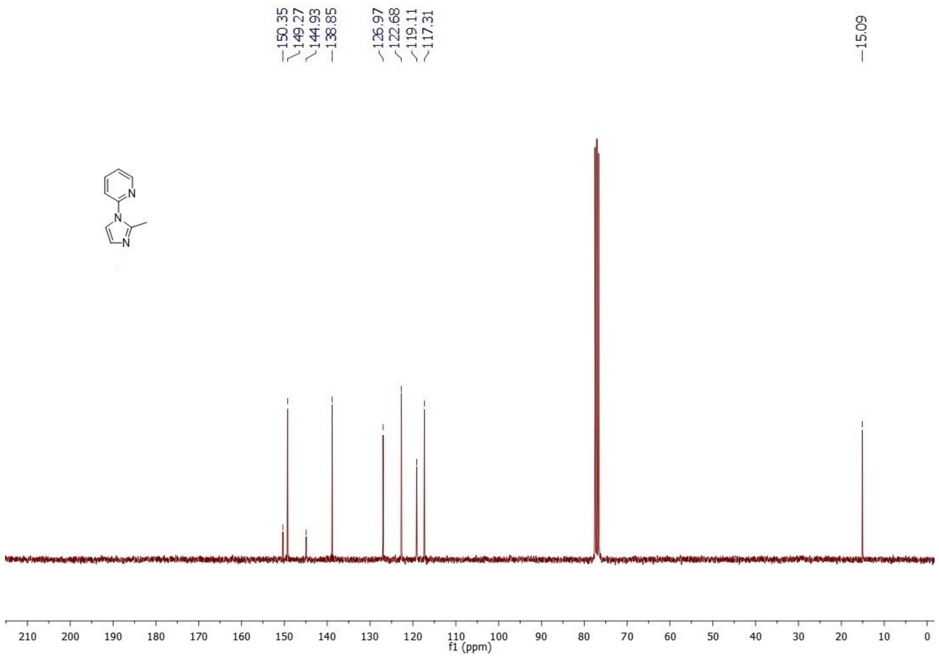

Figure 14. ^{13}C NMR spectrum (75 MHz) of 2-(2-methyl-1*H*-imidazol-1-yl)pyridine in $CDCl_3$.

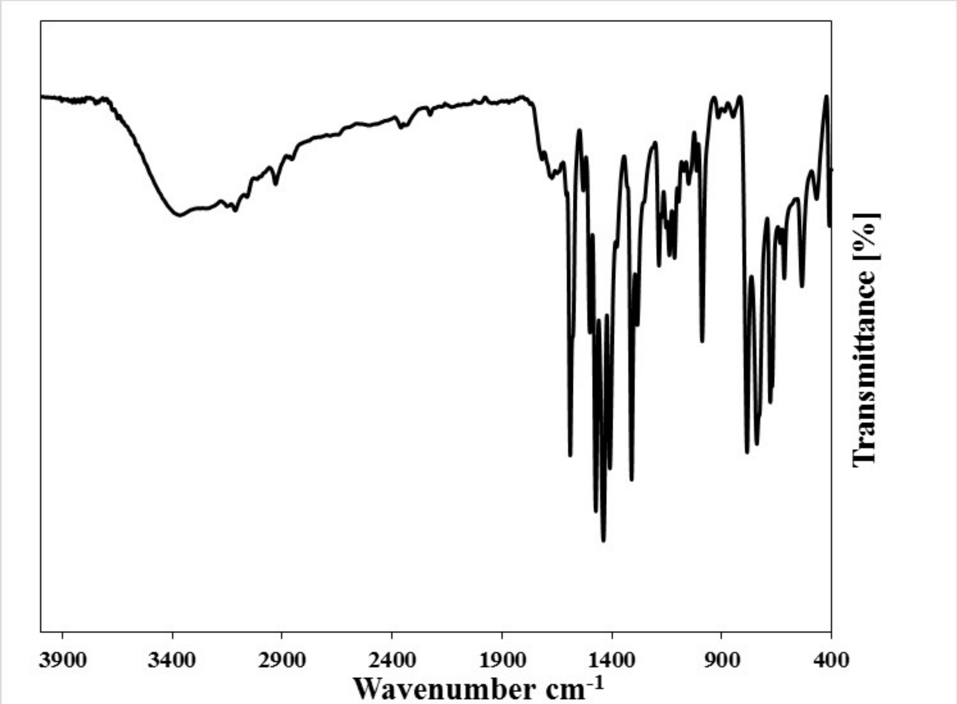

Figure 15. IR spectrum of 2-(2-methyl-1*H*-imidazol-1-yl)pyridine in KBr.

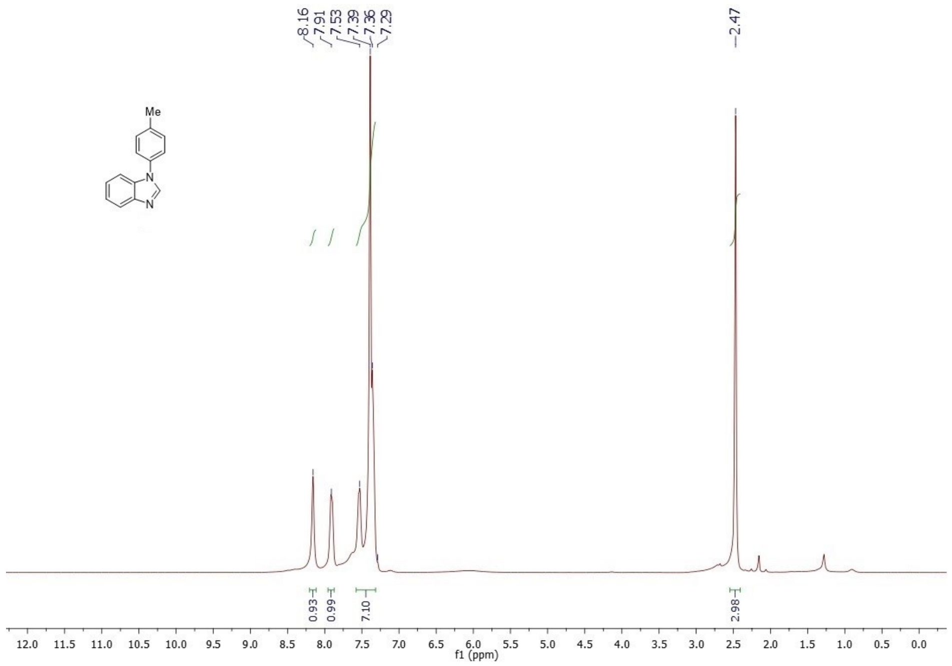

Figure 16. ^{1}H NMR spectrum (300 MHz) of 1-(*p*-tolyl)-1*H*-benzo[*d*]imidazole in $CDCl_3$**.**

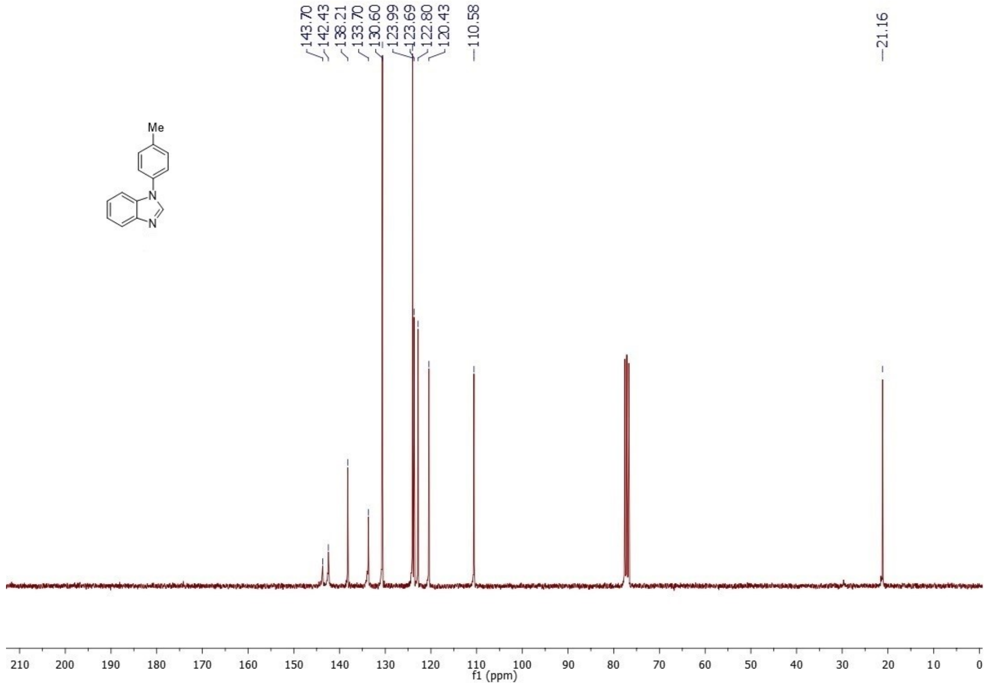

Figure 17. ^{13}C NMR spectrum (75 MHz) of 1-(*p*-tolyl)-1*H*-benzo[*d*] imidazole in $CDCl_3$.

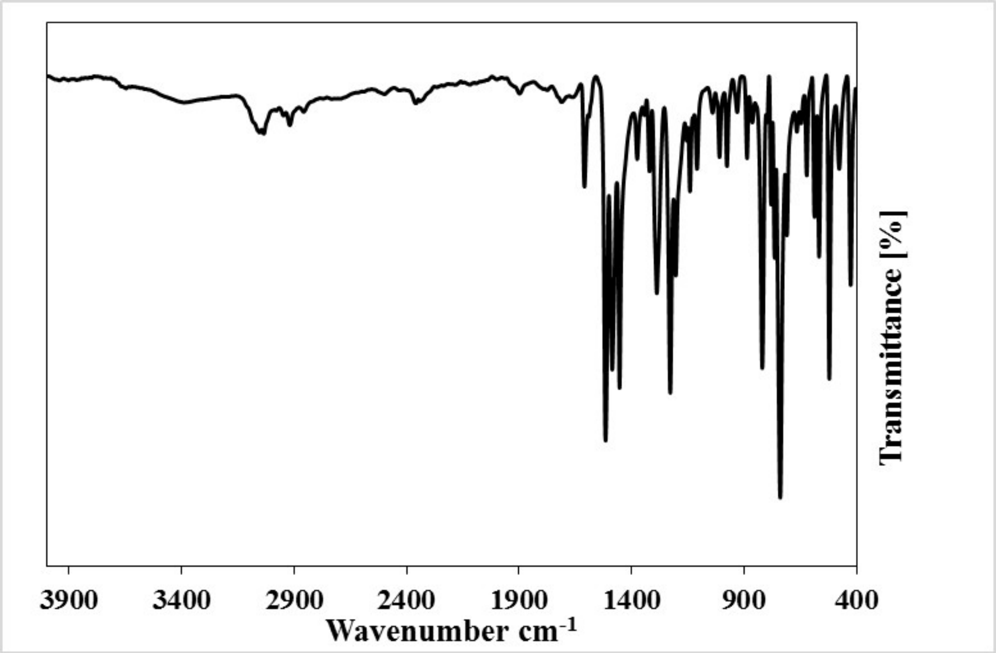

Figure 18. IR spectrum of 1-(*p*-tolyl)-1*H*-benzo[*d*]imidazole in KBr.

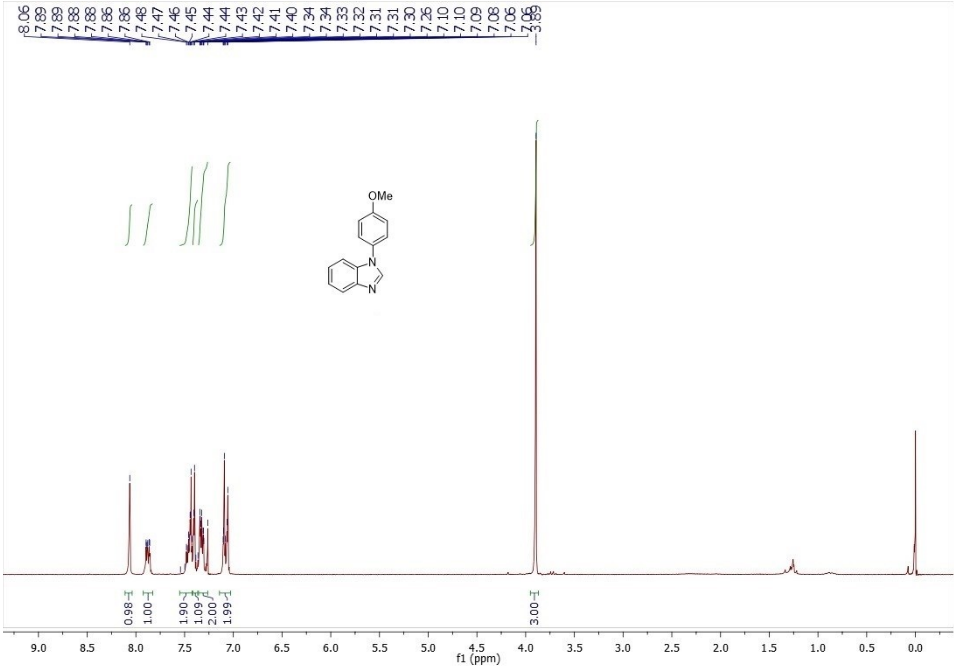

Figure 19. ^{1}H NMR spectrum (250 MHz) of 1-(4-methoxyphenyl)-1*H*-benzo[*d*]imidazole in $CDCl_3$.

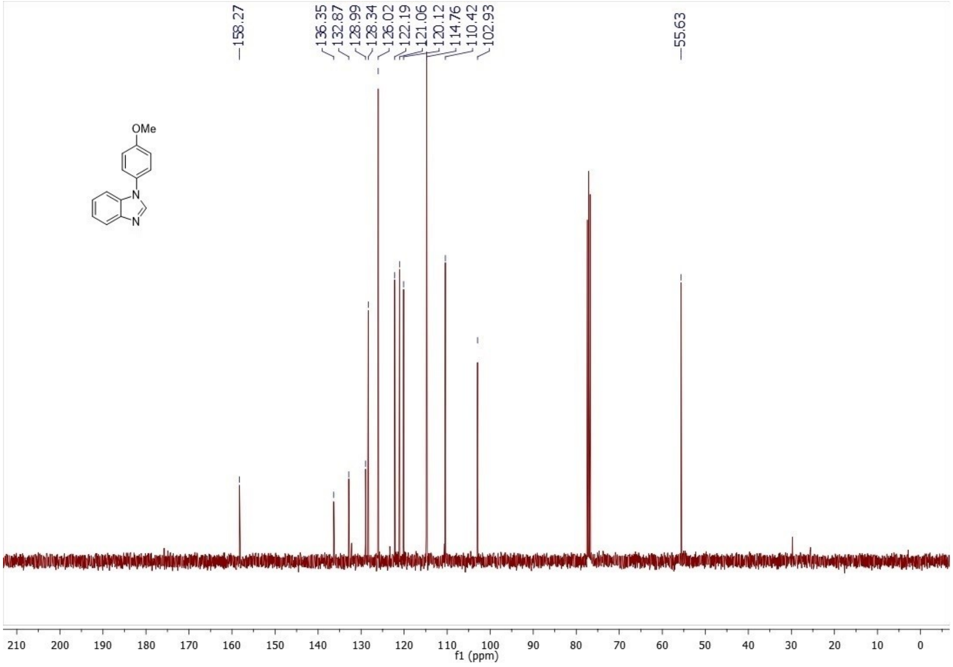

Figure 20. ^{13}C NMR spectrum (101 MHz) of 1-(4-methoxyphenyl)-1*H*-benzo[*d*]imidazole in $CDCl_3$.

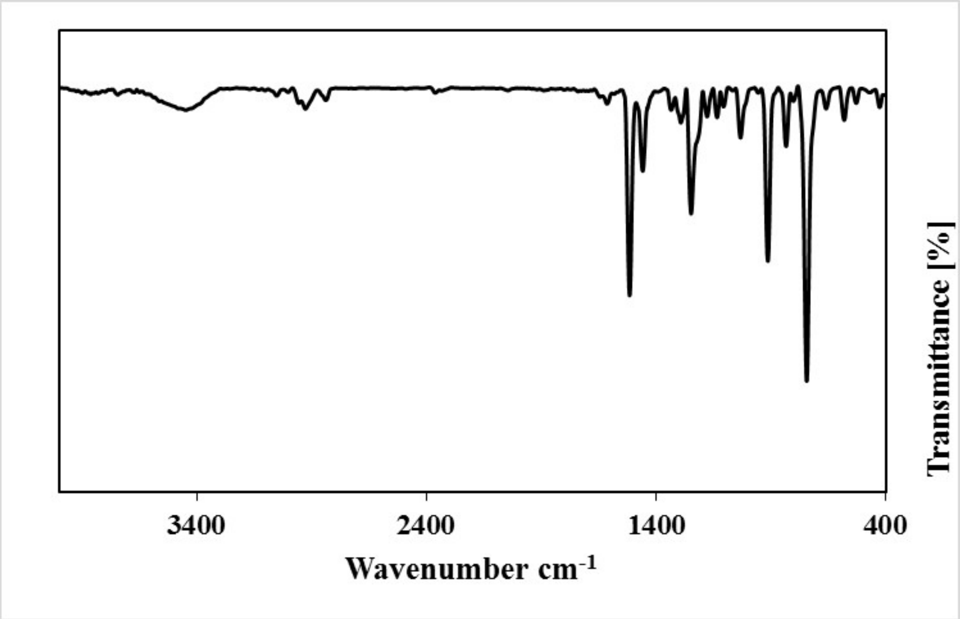

Figure 21. IR spectrum of 1-(4-methoxyphenyl)-1*H*-benzo[*d*]imidazole in KBr.

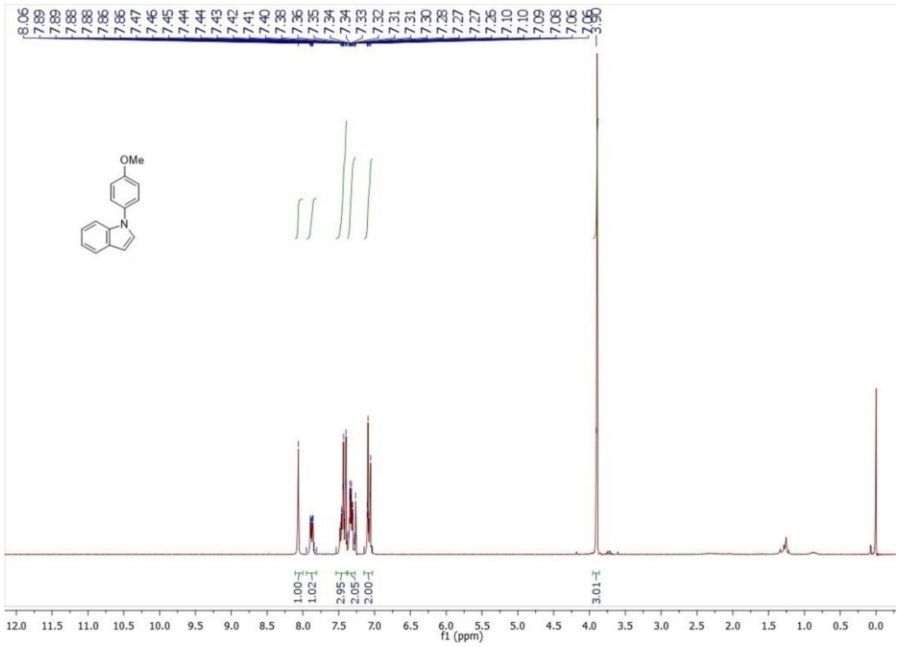

Figure 22. ^{1}H NMR spectrum (250 MHz) of 1-(4-methoxyphenyl)-1*H*-indole in $CDCl_3$**.**

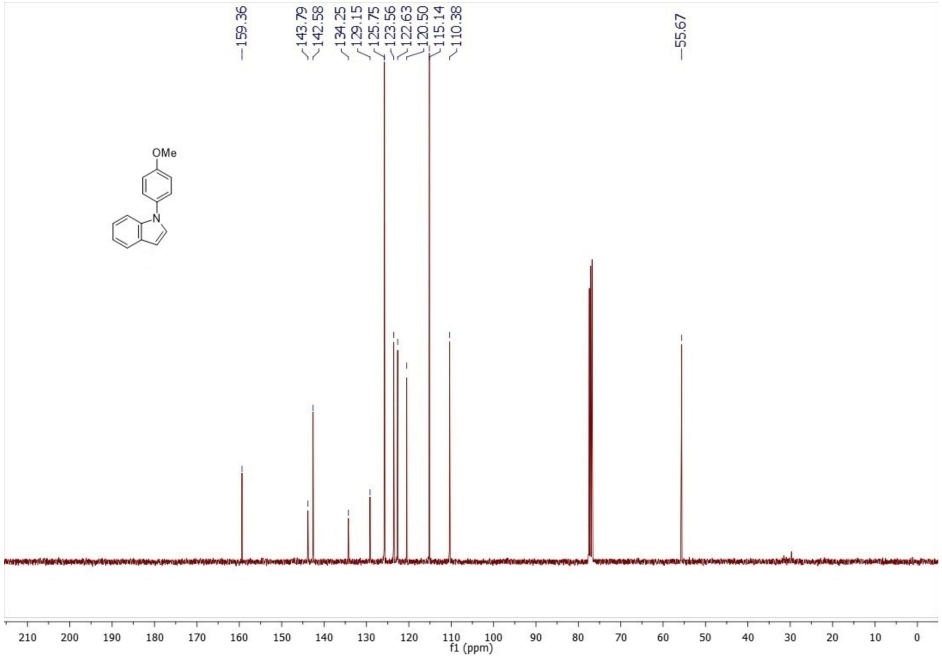

Figure 23. ^{13}C NMR spectrum (101 MHz) of 1-(4-methoxyphenyl)-1*H*-indole in $CDCl_3$.

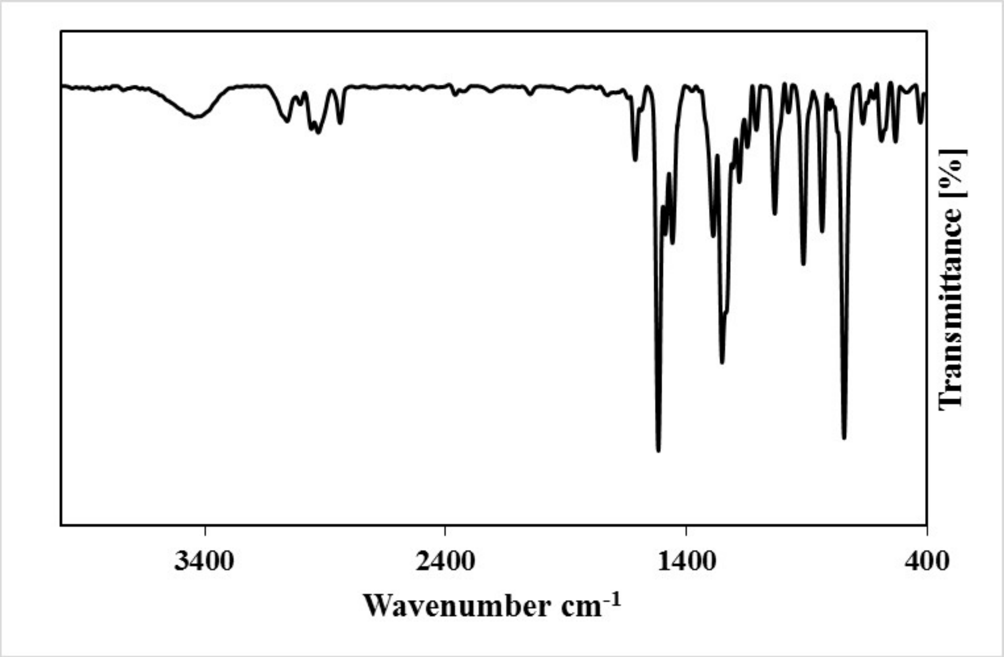

Figure 24. IR spectrum of 1-(4-methoxyphenyl)-1*H*-indole in KBr.

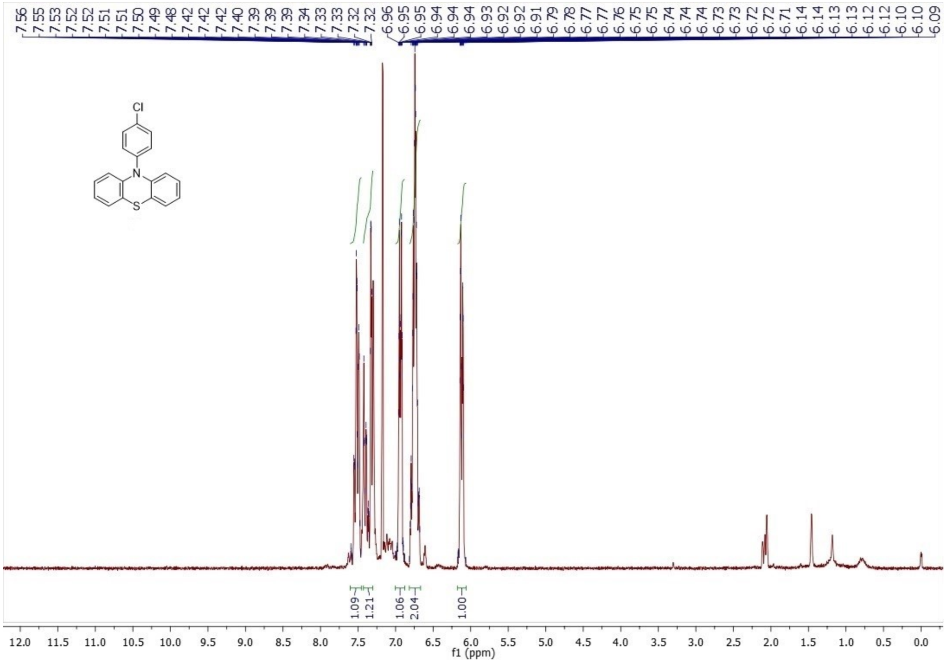

Figure 25. ^{1}H NMR spectrum (250 MHz) of 10-(4-chlorophenyl)-10*H*-phenothiazine in $CDCl_3$**.**

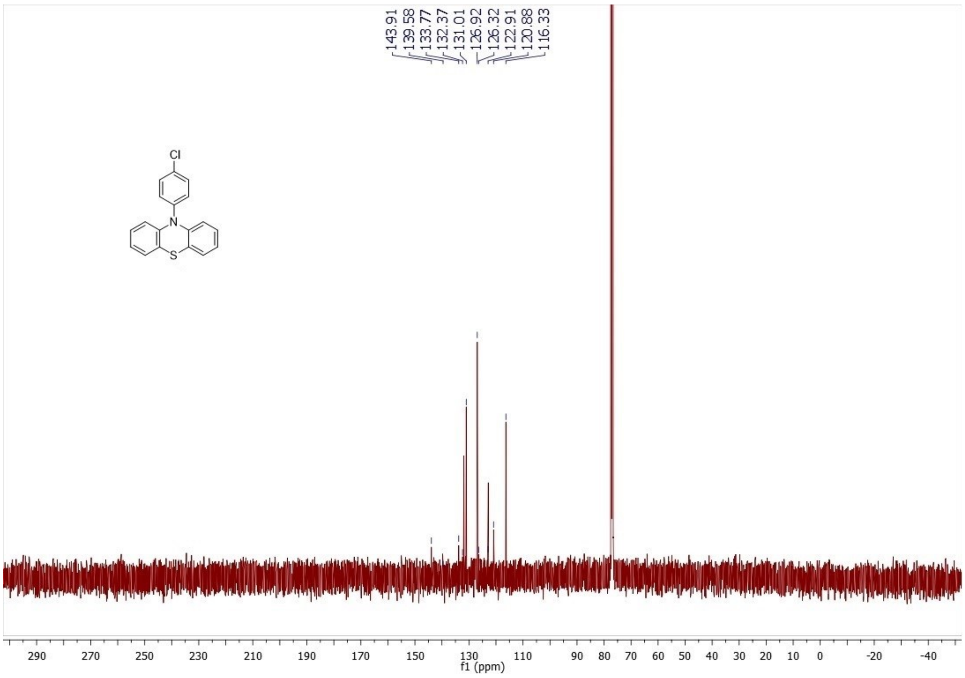

Figure 26. ^{13}C NMR spectrum (101 MHz) of 10-(4-chlorophenyl)-10*H*-phenothiazine in $CDCl_3$.

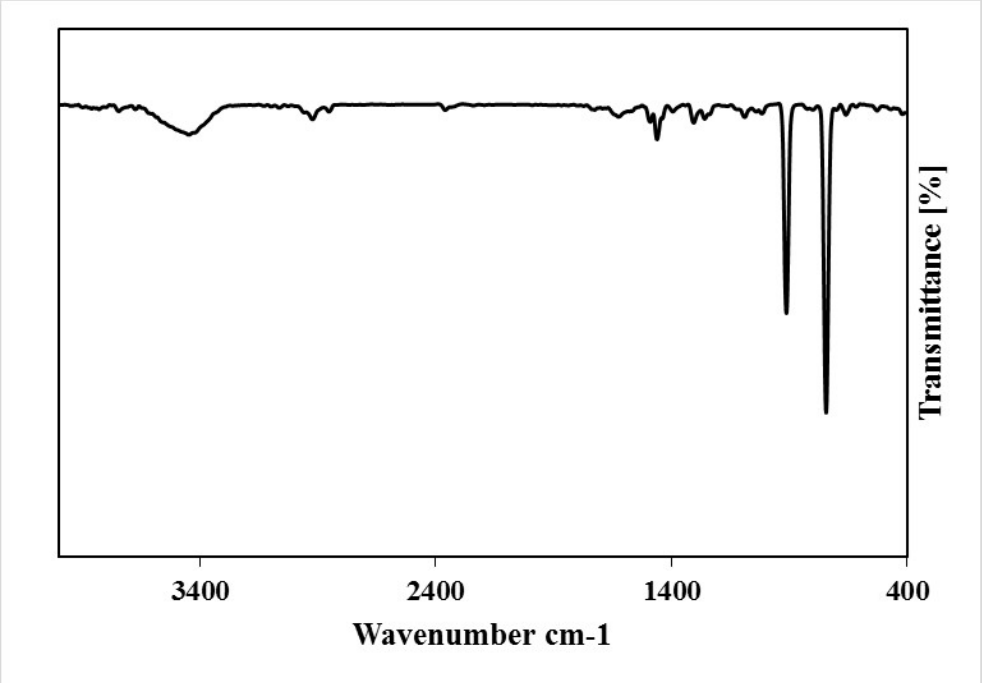

Figure 27. IR spectrum of 10-(4-chlorophenyl)-10*H*-phenothiazine in KBr.

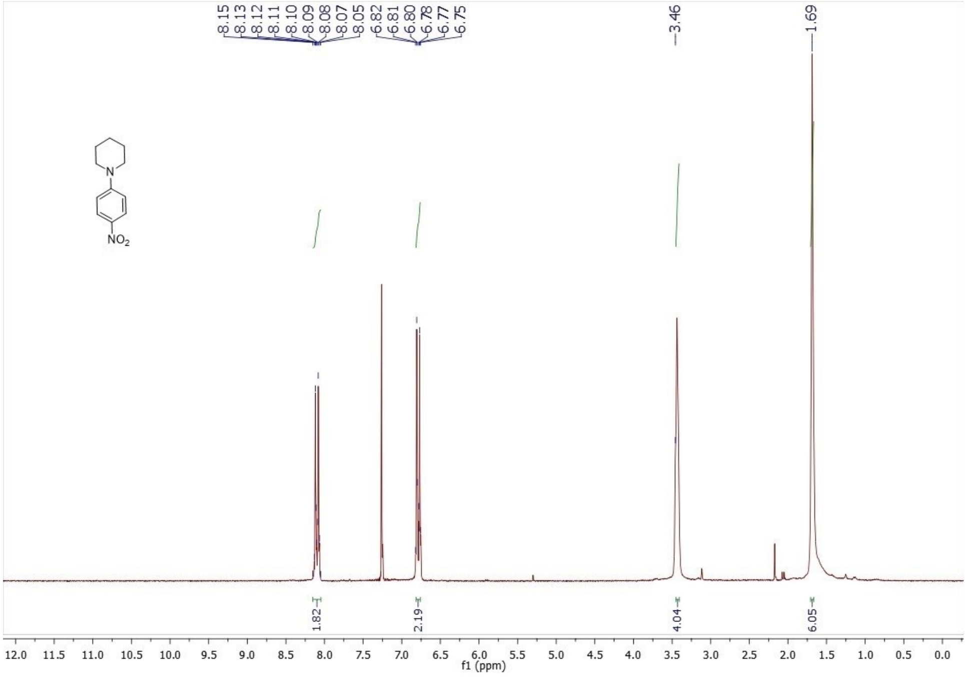

Figure 28. ^{1}H NMR spectrum (250 MHz) of 1-(4-nitrophenyl)piperidine in $CDCl_3$**.**

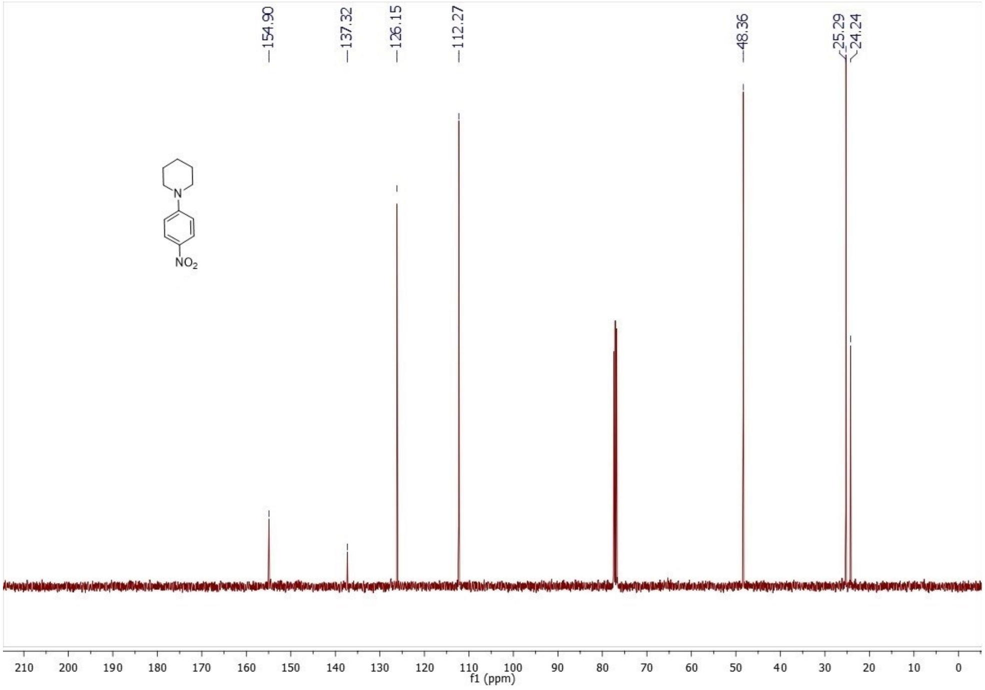

Figure 29. ^{13}C NMR spectrum (101 MHz) of 1-(4-nitrophenyl)piperidine in $CDCl_3$.

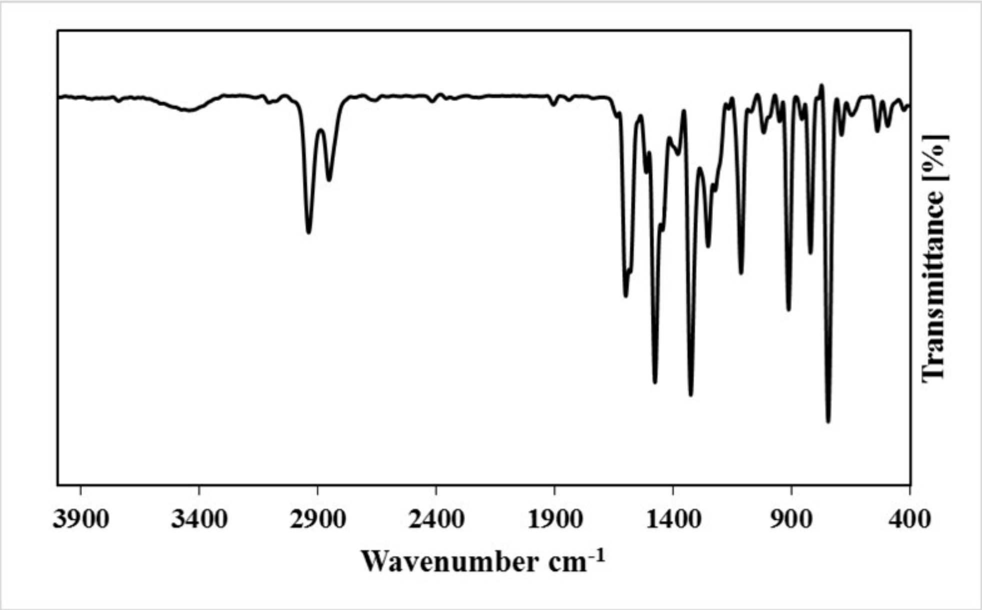

Figure 30. IR spectrum of 1-(4-nitrophenyl)piperidine in KBr.

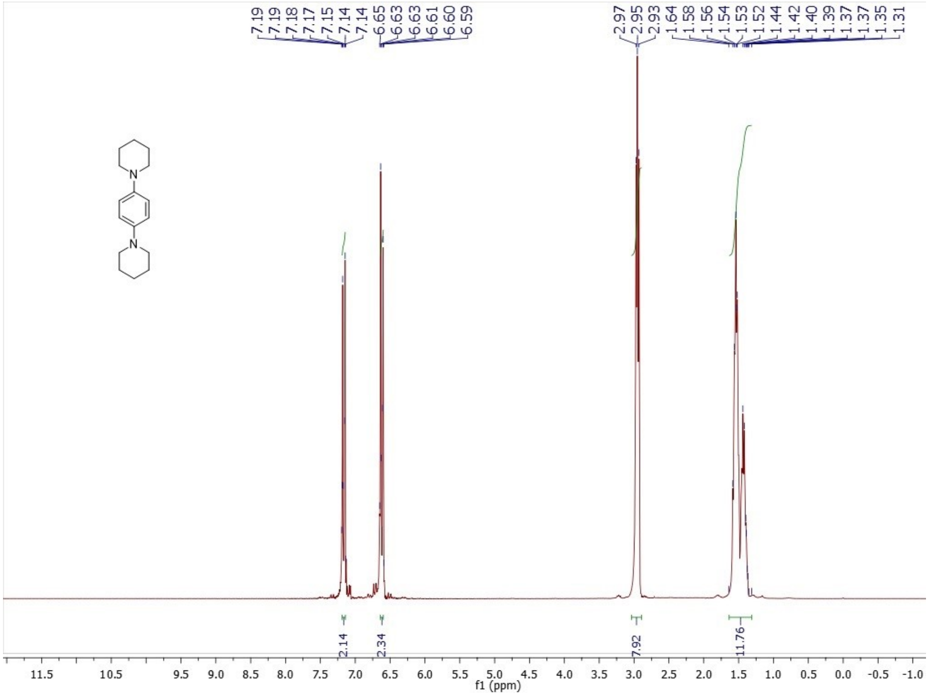

Figure 31. ^{1}H NMR spectrum (250 MHz) of 1,4-di(piperidin-1-yl)benzene in $CDCl_3$**.**

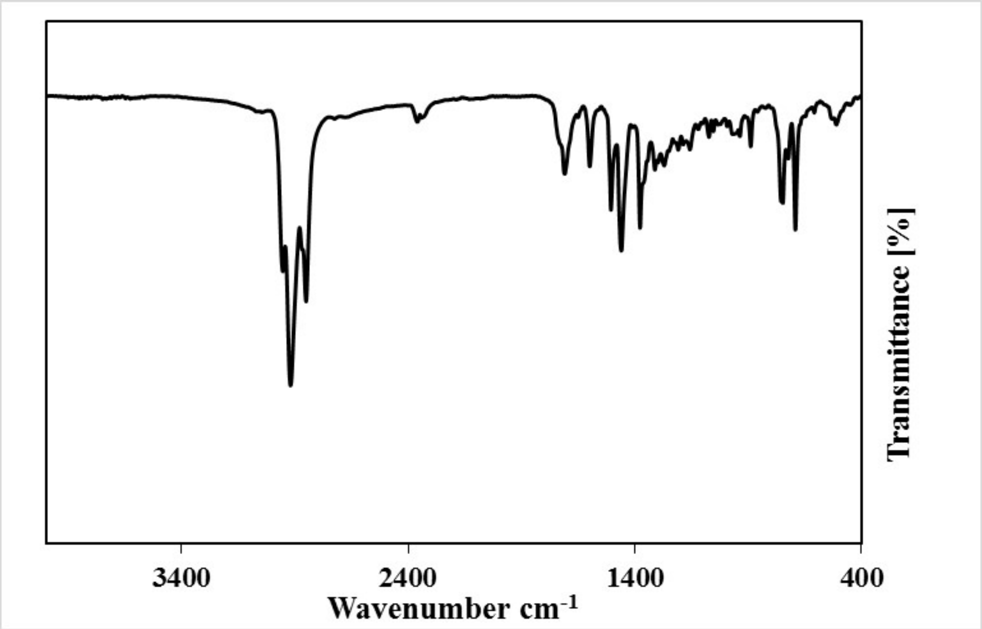

Figure 32. IR spectrum of 1,4-di(piperidin-1-yl)benzene in KBr.

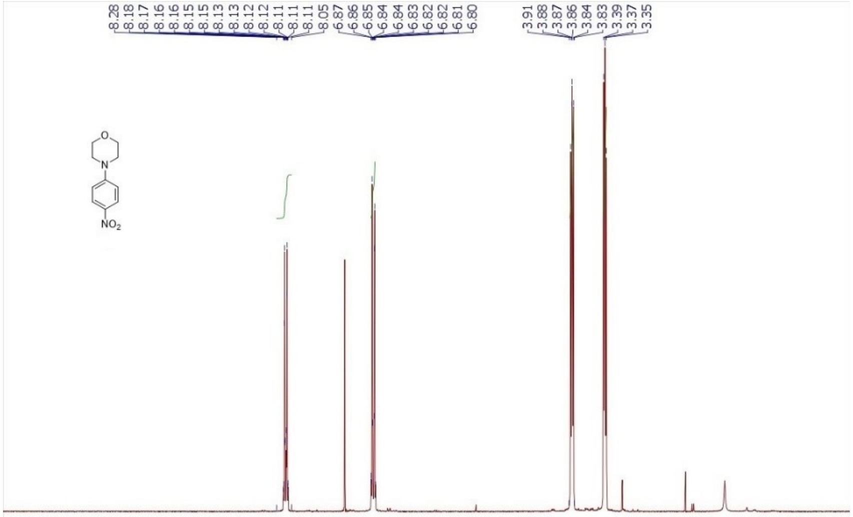

Figure 33. ^{1}H NMR spectrum (250 MHz) of 4-(4-nitrophenyl)morpholine in $CDCl_3$**.**

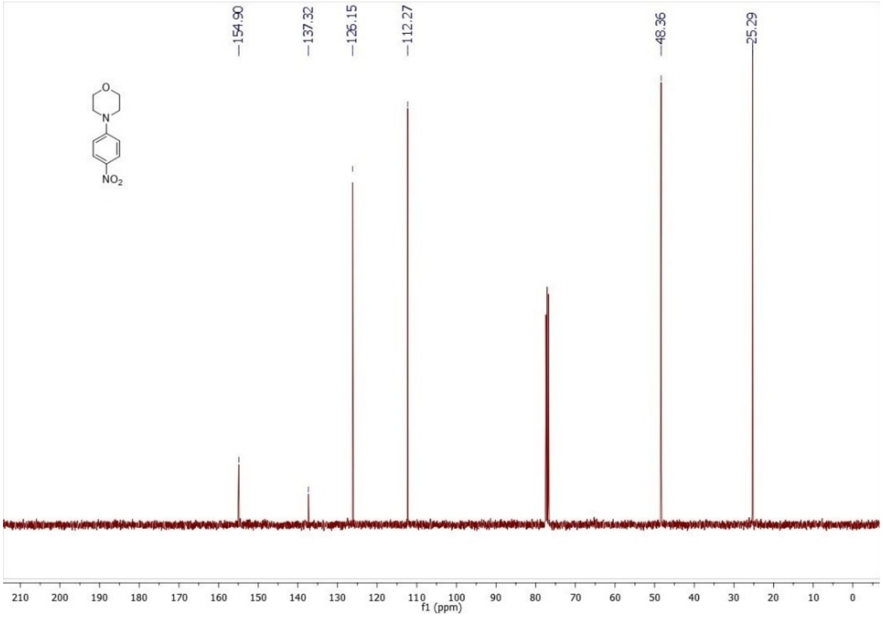

Figure 34. ${}^{13}C$ NMR spectrum (101 MHz) of 4-(4-nitrophenyl)morpholine in $CDCl_3$.

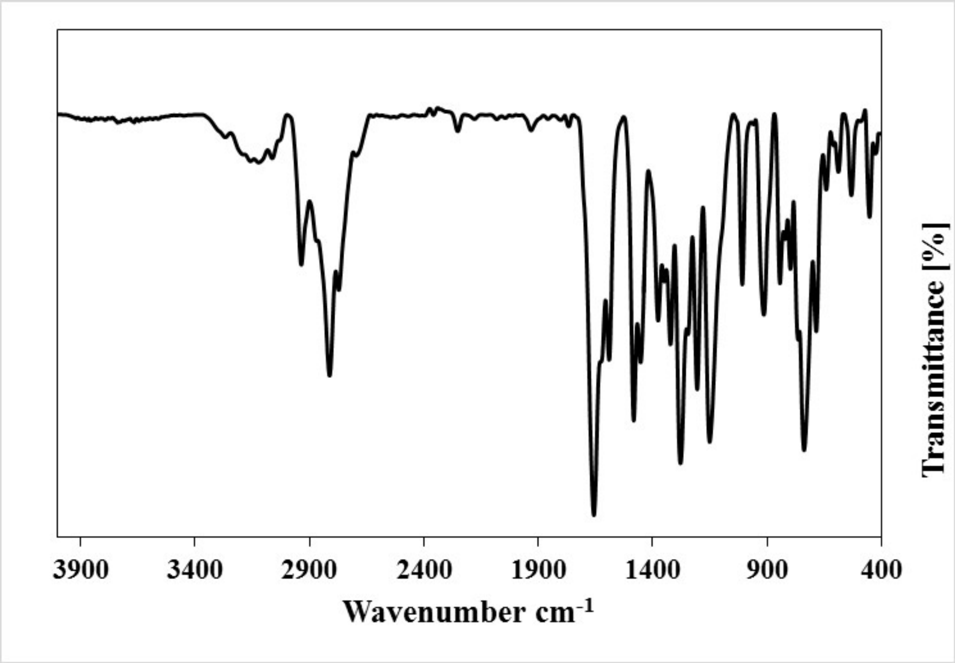

Figure 35. IR spectrum of 4-(4-nitrophenyl)morpholine in KBr.
